# Supplementary figures and images for: Morphological and molecular evidence for first records and range extension of the Japanese seahorse, Hippocampus mohnikei (Bleeker 1853) in a bay-estuarine system of Goa, central west coast of India
Source: PLoS One. 2020 Mar 24;15(3):e0220420. doi: 10.1371/journal.pone.0220420 (PMC7092974; doi:10.1371/journal.pone.0220420)

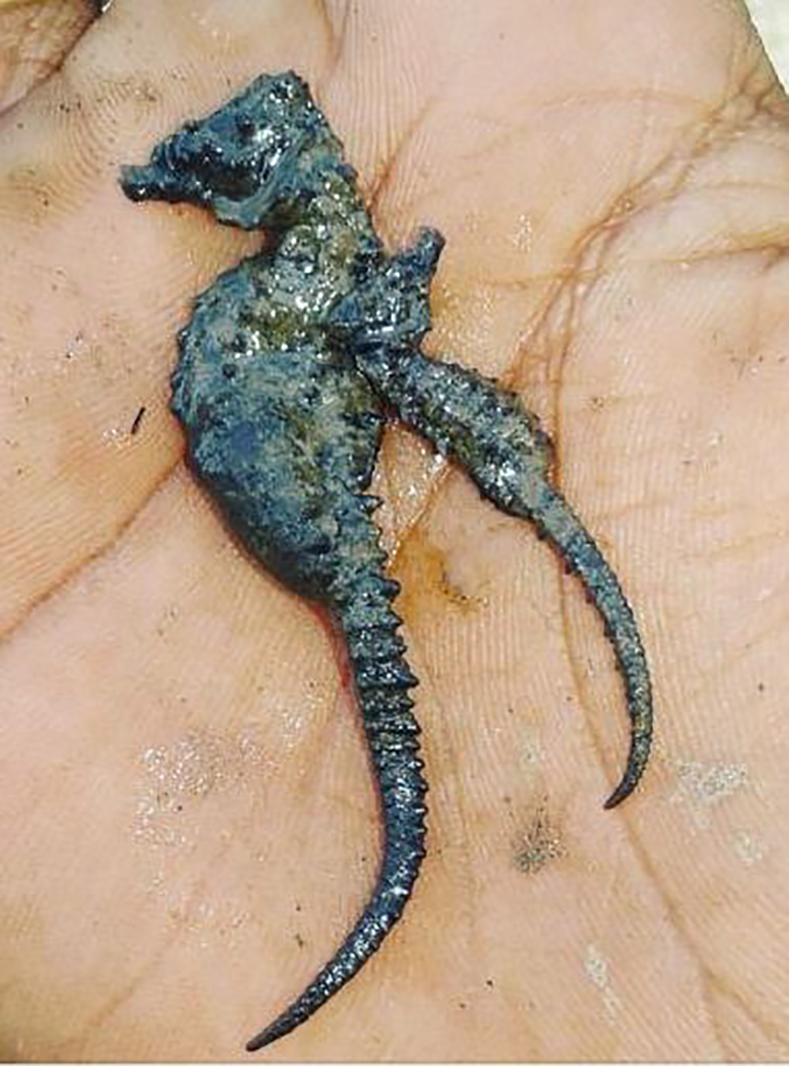

Supplement: S1 Fig — Male specimen of Hippocampus mohnikei caught by gill net in the Chapora estuary, Goa, India. (TIF) [file pone.0220420.s001.tif]
