# Supplementary material for: Morphological and molecular evidence for first records and range extension of the Japanese seahorse, Hippocampus mohnikei (Bleeker 1853) in a bay-estuarine system of Goa, central west coast of India
Source: PLoS One. 2020 Mar 24;15(3):e0220420. doi: 10.1371/journal.pone.0220420 (PMC7092974; doi:10.1371/journal.pone.0220420)
Supplement: Attachment — Submitted filename: PONE-D-19-19415_Review2Reviewer3.pdf [file pone.0220420.s006.pdf]

PLOS ONE

Morphological and molecular evidence for range extension and first occurrence of the Japanese seahorse, Hippocampus mohnikei (Bleeker 1853) in a bay-estuarine system of Goa, central west coast of India

--Manuscript Draft--

Manuscript Number:	PONE-D-19-19415R1
Article Type:	Research Article
Full Title:	Morphological and molecular evidence for range extension and first occurrence of the Japanese seahorse, Hippocampus mohnikei (Bleeker 1853) in a bay-estuarine system of Goa, central west coast of India
Short Title:	Range expansion of the Japanese seahorse
Corresponding Author:	Anantha Sreepada Rayadurga, M.Sc<br>National Institute of Oceanography CSIR<br>Panaji, Goa INDIA
Keywords:	Hippocampus mohnikei; cytb gene; COI gene; distribution; Indo-Pacific; genetic distance; habitat; west coast of India, ocean circulation, phylogenetic analysis
Abstract:	Accurate information of taxonomy and geographic range of seahorse species (genus Hippocampus) is the first step in preparing threat assessments and designing effective conservation measures. Here, we report the range expansion and first occurrence of the Japanese seahorse, Hippocampus mohnikei (Bleeker, 1853) from the Mandovi estuarine ecosystem of Goa, central west coast of India (CWCI) based on morpho-molecular analyses. The morphometric and meristic traits particularly, short snout (29–35% head length), double cheek spine, low coronet and long tail (51.2–57.9% of standard length), 11 trunk rings, 37–39 tail rings, 15–16 dorsal and 12–14 fin rays observed in four collected specimens matched with the reported key diagnostic morphological criteria of vouchered specimens of H . mohnikei . The seahorse mitochondrial cytochrome oxidase subunit I (COI) and cytochrome b (Cyt b) genes were partially sequenced for conclusive genetic identification of the species under study. Molecular analysis showed that all four individuals of seahorse species clustered together suggesting a monophyletic lineage. Using the maximum similarity with GenBank database, maximum likelihood network and subsequent morphological analysis, the identity of the collected seahorse species was reconfirmed as H. mohnikei . With this new report, the geographic range of H. mohnikei extended significantly to the westward side from its previously known range. This new sighting of H. mohnikei could indicate a long-distance dispersal facilitated by due to prevailing oceanic circulation in the Indo-Pacific region or increased habitat suitability in bay-estuarine systems of Goa, CWCI. Comparison of the pair-wise genetic distances (Kimura 2-parameter) based on COI and Cyt b sequences revealed that the specimens examined in this study are genetically closer to H. mohnikei populations from Vietnam and Thailand than they are to those in Japan and China. To test the hypothesis whether H. mohnikei are vagrants or previously unreported established population, long-term inter-annual sampling and analyses are warranted.
Order of Authors:	Sushant Vilas Sanaye
	Rakhee DS Khandeparker
	Anantha Sreepada Rayadurga, M.Sc
	Mamatha Singanahalli Shivaram
	Harshada Kankonkar
	Jayu Narvekar
	Mukund Gauthankar
Response to Reviewers:	Response to Comments/suggestions made by the Reviewers<br><br>Manuscript ID number: PONE-D-19-19415

Powered by Editorial Manager® and ProduXion Manager® from Aries Systems Corporation

Manuscript title: Morphological and molecular evidence for range extension and first occurrence of the Japanese seahorse, Hippocampus mohnikei (Bleeker 1853) in a bay-estuarine system of Goa, central west coast of India

Authors: Sushant Sanaye, Rakhee Khandeparker, Rayadurga Anantha Sreepada, Mamatha Singanahalli Shivaramu, Harshada Kankonkar, Jayu Narvekar, Mukund Gauthankar

We are thankful for the reviewers for their constructive criticisms/suggestions and their input has substantially improved the quality of the manuscript.

Below, we provide a point-by-point response as to how the feedback received from the Reviewers has been incorporated in the revised manuscript.

This manuscript is a resubmission of manuscript PONE-D-19-19415 with our response to the questions made by the reviewers. The Academic Editor handling this manuscript is Dr. Rui Rosa

Reviewer #1:

Morphological and molecular evidence for range extension and first occurrence of the Japanese seahorse, Hippocampus mohnikei (Bleeker 1853) in a bay-estuarine system of Goa, central west coast of India

The authors report of a seahorse, Hippocampus mohnikei usually found in the east Indian and Pacific ocean, recorded for the first time on the west coast of India. Overall, the study is interesting in its findings of range expansion of a small seahorse.

Response: We thank the Reviewer for the excellent review and the positive comments on the manuscript. Constructive suggestions provided by the Reviewer are appreciated.

General comments:

Comment: I think the Authors did not read the Guidelines for submission?. Some sections are messy with Table captions missing or wrongly placed, Fig captions interrupting main text of MS. It was hard to follow and I put it away many times and nearly did not review!. Someone needs to check if all uploaded files are correct before sending to Reviewers. Regarding the manuscript I would urge the authors to separate Tables and Figures with their captions from the main text of the MS. Figure legends/captions occurred within the text and disrupted the flow. Example: Table 2 starts on page 13 without Table legend/caption followed by text (new results). The actual table was (I assume) on page 14 without a header and table legend was below the table!

Response: At the outset, we apologise for the inconvenience caused due to the formatting issues. Here we state that the all the guidelines/instructions for online submission of the manuscript were meticulously adhered to. According to journal requirement, we have again checked our revised manuscript to meet PLOS ONE’s style requirements. We hope that the revised version will be the error-free readable format.

Comment: Another point for the morphological measurements; how about indicating the different measurements on one of the images of the seahorse with abbreviations (explained in Fig caption) e.g. see Forsgren & Lowe 2006 (seadragon morphology). Table2 would be more compact.

Response: Highly appreciate constructive suggestion of the Reviewer. The measurement of morphological characters and meristic counts of seahorse specimens were carried out as per the standard protocols/procedures of measuring seahorses as outlined in Lourie et al. (1999); Lourie et al (2003) and Lourie et al (2004). These are standard protocols that have been used widely for the purpose. measurement of morphological and meristic characters of seahorse species by many authors. These

Powered by Editorial Manager® and ProduXion Manager® from Aries Systems Corporation

references have been cited in sub-section, morphological analysis under Section, 'Materials & Methods' which we consider serve the purpose.

Comment: For the molecular work did you extract and sequence mitochondrial DNA (mtDNA) from all 4 individuals found or just from 2 individuals? I thought you caught 4? If you have 4 samples sequenced (COI and cytb) you should show all 4 in the tree not just the one. Where do the other 3 individuals occur in each tree (COI and cytb)? If all or some of the other 3 do not cluster with the voucher H. mohnikei then you have to consider other possibilities.

Response: We strongly appreciate the criticism provided by the Reviewer. As suggested by the reviewer, remaining 3 seahorse specimens (MK1, MK2 and MK3) were also sequenced in addition to originally sequenced seahorse specimen (MK4) for both COI and Cyt b genes. Sequences generated from 04 seahorse specimens with GenBank accession numbers, MN595216, MN595218, MN595217 and MK330041.1 for COI gene and MN595213, MN595214, MN595215 and MK112274.2 for Cyt b genes, respectively corresponding to 4 seahorse specimens (MK1, MK2, MK3 and MK4) were included in phylogenetic analysis (Fig. 6 and Fig. 7). Phylogenetic analysis revealed that all 4 seahorse individuals from Goa clustered together confirming a monophyletic lineage. Sequences generated of COI and Cyt b gene from 4 seahorse individuals have been used for phylogenetic analysis in the revised manuscript.

Comment: Some sections need to be deleted especially in Results! Result section is littered with sentences that end in eg '…. presented in Table x' = not in published papers (more like student assignment). Please explain your results and refer to your table/Fig in brackets! Eg. Morphological measurements revealed ….. state your results (Table 1). Check grammar, punctuation in main text.

Response: We have greatly modified the Results section as suggested by the Reviewer. The section of Results has been modified in the revised manuscript. We incorporated all grammatical and typographical edits into the revised text. The English language has been polished after incorporating the suggested grammatical and typographical edits into the text. Now, the English language of the manuscript has been checked by Grammarly software.

Specific comments:

Introduction

Query: Line 57: Sentence; Often overlooked by most fishermen, due to their cryptic nature, their ability to camouflage and the sparse distribution restrict biologists to identify their presence in thick coastal marine habitats.

Response: As suggested by the reviewer, the sentence has been corrected.

Query: Line 100: Sentence about using gene markers re-write … sequenced 2 mtDNA gene regions (loci) COI & cyt b ..

Response: The sentence corrected as suggested by the Reviewer.

Materials & Methods

Query: Line126: incidental catch = is that by catch - if so use correct term

Response: Correct terms used. In the revised manuscript, the sentence corrected as Dead seahorses landed as incidental catch in bag nets attached to the stakes operated by local fishermen ....

Query: Lines 132-141: Figure captions! Should be on separate sheet!

Response: The MS has been prepared as per journal's prescribed format. Accordingly, the tentative probable positioning of the Figures with captions has been shown in the manuscript

Powered by Editorial Manager® and ProduXion Manager® from Aries Systems Corporation

Query: Line 175: '.fin from fresh seahorse ..' I thought they were dead seahorses from by catch? Did I miss something?

Response: Actually, we wanted to infer freshly dead seahorses here. In the revised manuscript, this has been corrected as freshly landed dead seahorses' to bring clarity.

Query: Line 203: Seq of Goa specimens GB MK330041.1 and MK112274.2 = 2 seq's submitted - did you not seq 4 individuals? If the other 2 did not provide DNA- seq (were, they degraded?) you need to say this in your M&M! add a Table S1 (suppl) of the sequences (species name, GB accession # Ref) you downloaded for your phylogenetic tree from GB and cite their work.

Response: We are extremely grateful to the Reviewer for pointing out this mistake. As explained above, remaining 3 individual seahorses (MK1, MK2, MK3) were sequenced subsequently following the same protocols as that was followed for initially sequenced seahorse individual i.e. MK4. Sequences generated from all 4 seahorse individuals, MK1, MK2, MK3 and M were submitted at NCBI vide GenBank accession numbers, MN595216, MN595218, MN595217 and MK330041.1 for COI gene and MN595213, MN595214, MN595215 and MK112274.2 for Cyt b genes, respectively were included in phylogenetic analysis (Fig. 6 and Fig. 7).

As advised by the reviewer, all the downloaded sequences of H. mohnikei for COI and Cyt b genes available at GenBank along with their voucher Number and references used for constructing phylogenetic trees has been provided Supplementary S1 Table.

Results

Query: Line 236-237: delete sentence! Start explaining your main result and refer to your (Table/Fig) in brackets. 'Morphological measurements revealed …Goa specimens are consistent with … (Table1). I would move Table 1 into Suppl material (raw data). Then describe some of the more important measurements that support your work. Please don't repeat results in your text if they are already in Table 1! People can read tables. So explain the measurements eg. on average size of … (mean #) comparable to size of mohnikei samples from eg. east India or Thailand/Vietnam? Line 258-259: re-write sentence … we compared … commonly occurring seahorses, H. kuda … (Table 2).
Response: As advised by the reviewer, Line, 236-237 has been deleted. Now, the sub section on 'morphological analysis' under Results section has been modified to include only the salient findings. We have modified the Results section to a great extent and hope to have improved it by doing so.

Firstly, we have re-arranged the few paragraphs to better mirror the presentation sequence of the results. Additionally, we have introduced Table 3 "Comparison of diagnostic morphological characters of all vouchered specimens of Hippocampus mohnikei" for comprehensiveness of the morphological data. Since, Table 1 describes all the morphological characters of four seahorse specimens collected from Goa coast. Considering its significance to the manuscript, Table 1 has retained and not moved to Supplementary material. All other typos and corrections suggested by the Reviewer have been incorporated in the revised manuscript.

Query: Pages 11-12, and 13-14 totally messy with tables throughout text making it difficult to follow should have been on separate sheet.

Response: We apologise for the inconvenience caused due to formatting issues. The revised manuscript has been checked to meet PLOS ONE's style requirements.

Query : Page 13-14 = messy table is split I assume? No captions above table header - I assume Table 2? AND Table caption go ABOVE a table not below please read Plos One Author Guidelines!

Response: We apologise for the inconvenience caused due to formatting issues. Now, the revised manuscript has been checked to meet PLOS ONE's style requirements and hope the formatting issue is resolved.

Powered by Editorial Manager® and ProduXion Manager® from Aries Systems Corporation

Query : Table 3 - PCA – move to Suppl material it is already represented in Fig 4 – don't repeat results.

Response: As suggested by the Reviewer, Table 3 on PCA output has moved to Supplementary material (S2 Table)

Query: Phylogenetic analysis – needs overhaul. Both trees (Fig. 5 & 6) only have 1 goa individual in tree. Where are the other 3 sequences? You need to add all 4 indiv seq to each gene region (COI, cyt b) tree. If by chance other individuals did not fall into same clade as H mohnikei – this needs to be discussed! There are probably other reasons for it …(eg. hybrids?). When you aligned the seq to the other GB downloaded seq in MEGA it should be obvious especially COI (Barcode of Life – check out H mohnikei on BOLD systems http://www.boldsystems.org/index.php/TaxBrowser_TaxonPage? taxid =66243)

Response: The subsection of 'Phylogenetic analysis' has been modified to a great extent by incorporating the sequence data of all 4 individual seahorses from Goa. Maximum likelihood (ML) trees for both COI and Cyt b genes have been constructed by aligning them with the downloaded sequences for all vouchered H. mohnikei downloaded from the GenBank in the revised manuscript. Phylogenetic analysis revealed that all 4 seahorse individuals from Goa clustered together and formed a single clade thus confirming a monophyletic lineage. This has been now mentioned in the revised manuscript. As advised by the reviewer, availability of sequences of COI gene for H. mohnikei were also checked in Barcode of Life (BOLD Systems).

Query: Line 317 – delete 'depicted in Fig. 5 and Fig. 6… see above comments

Response: Deleted
Query: Line 325 – delete presented in Table

Response: Deleted

Query: Lines 330-338 = remove Fig captions from main MS text – refer to guidelines.

Response: Figure captions removed from the main MS text.

Query: Table 4 (don't just copy table from program) = Table Header replace 1 2 3 4 with actual locations: Goa Japan Same with Table 5.

Response: As suggested by the reviewer, the Tables of genetic distance (Tables 4 & 5) have been reformatted in the revised manuscript.

Query: Line 317-372 Fig caption see guidelines.

Response: Done

Discussion

Query: Line 390-394 Fig caption – see guidelines

Response: Done

Query: There are repeated results in the discussion – delete them – you already have these in your Result section. Discuss your results.

Response: As suggested by the Reviewer, results have been deleted from the Discussion Section.

Query: Line 405-415 remove results repeated – already in Result section Fig. 2 and Fig 3 = combine or place one in suppl material Figure 1 & 8 combine = Fig 1 could be inset to the bigger Map Fig 8.

Response: Following the comments made by the Reviewer, we have modified the discussion comprehensively. in the revised manuscript. Now, it is hoped the

Discussion has improved by removing the repetition of Results. We tried to combine the Fig. 2 and Fig. 3, the quality of the Figure was lost. Similarly, we tried to integrate Fig. 1 to bigger map of Figure 8, but it did not work properly in terms of picture quality and alignment.

Reviewer #2:

Comment: Really nice description of morphological and molecular characters for the range expansion of H. mohnikei in India. This is a really interesting seahorse that requires more studies on its distributional range within the Indo-Pacific region.

Response: We thank the Reviewer for the excellent review with positive comments and appreciation of our work. We agree with the Reviewer that further studies on the distributional range of H. mohnikei within Indo-Pacific region in collaboration with other researchers in Southeast Asia would help in better understanding its evolutionary and biogeography.

Reviewer #3:
General Comments

Comment: This paper represents a significant contribution to the global knowledge of seahorse diversity in the Arabian Sea, and represents novel information that is interesting from both evolutionary and conservation perspectives. I suggest that the authors revise the manuscript to have more of a focus on the biogeography of seahorses in Southeast Asia, which is outlined nicely by Lourie and Vincent (2004). [Lourie, S.A. and Vincent, A.C., 2004]. A marine fish follows Wallace's Line: the phylogeography of the three-spot seahorse (Hippocampus trimaculatus, Syngnathidae, Teleostei) in Southeast Asia. Journal of Biogeography, 31(12), pp.1975-1985]. The manuscript is important and should be published, but requires a thorough re-write to correct the grammar (see specific comments below). The discussion needs to more clearly outline that this is likely to be an established population, but with the possibility that these specimens are vagrants.

Response: We gratefully thank the Reviewer for excellent review with positive comments/suggestions on the manuscript. As suggested by the Reviewer, the biogeography of seahorse populations in southeast Asia has been focussed in the revised manuscript. The paper by Lourie & Vincent (2004) dealing with the phylogeography of three-spotted seahorse, H. trimaculatus has been referred in the manuscript. Accordingly, the discussion on biogeography of seahorse populations has now been included in the discussion. Additional minor modifications have been made to improve overall readability and to clarify certain statements. The grammatical errors have also been fixed as per the specific comments/edits provided by the Reviewer. The reviewer may agree that more samples of H. mohnikei would be needed to arrive at a definite conclusion for ascertaining whether the present sighting is a previously unreported established populations or vagrants. Based on this present reporting, our efforts for more specimens of H. mohnikei are still being pursued. With more number of specimens from Goa waters, the biogeography of this small seahorse species in southeast Asia could be addressed comprehensively. Of course this would help in better understanding from both evolutionary and conservation view points.

Comment: The information regarding ocean circulation should be provided in the methods section, and figures should be added to display other seasons as well. This should then be linked in the discussion to how the species got to Goa. An effort should be made to determine where this species originates biogeographically and to clarify in which direction it dispersed over time.

Response: As suggested by the reviewer, now the Figure describing the ocean circulation has been moved to Materials & Methods section (Fig. 4). The role of the ocean circulation in the Indo-Pacific region in facilitating the dispersal of H. mohnikei from its previously reported geographical range into the coastal waters of Goa has been now discussed in the revised manuscript.

Comment: Overall, this manuscript requires a substantial amount of work to be made publishable, but I do not consider this a 'major revision', as the overall structure and

Powered by Editorial Manager® and ProduXion Manager® from Aries Systems Corporation

content need to change very little. I suggest publishing this manuscript in PLoS ONE after these extensive minor revisions have been made.

Response: The authors are thankful to the Reviewer for positive feedback on the manuscript. Constructive suggestions provided by the Reviewer are appreciated. Now the manuscript has been revised following the minor revisions suggested by the Reviewer.

Specific comments

Query: Line 27: use 'geographic range' rather than 'distribution range'; do this throughout the manuscript

Response: As suggested, ‘distributional range’ changed to ‘geographical range’ in the revised manuscript.

Query: Line 40: "hitherto known range of Japan and Vietnam" is incorrect. Aylesworth et al (2016) have already reported this species from the Gulf of Thailand and western Thailand.

Response: We honestly agree with the Reviewer’s comment. Now, the statement has been modified to accurately represent the fact in the revised manuscript.

Query: Line 43: 'conspecificity' refers to individuals within the same species; it appears here that the authors are suggesting that the specimens from Japan are of a different species. I suggest rewording to state that the specimens examined in this study are closer genetically to those in Vietnam and Thailand than they are to those in Japan. Unless there is evidence to the contrary, they are all conspecifics.

Response: Yes, Agreed. As suggested by the Reviewer, the statement has been reworded to genetically closer in the revised manuscript.

Query: Lines 56-57: 'unnoticeable from coastal habitat' is unwieldy. I suggest deleting this sentence.

Response: Sentence deleted as suggested.

Query: Lines 57-59: This is an incomplete sentence that lacks a subject; 'thick coastal marine habitats' is ambiguous. I suggest deleting this sentence as well.

Response: As suggested by the Reviewer, the sentence as been modified to convey the intended meaning.

Query: Line 60: at least three species have been described since Lourie et al. (2016): Hippocampus casscio, H. haema, and H. japapigu

Response: We totally agree with the Reviewer and we are sorry for missing out this. With the reporting of additional 3 seahorse species since Lourie et al (2016), the statement has been modified to include this. i.e. 44 instead of 41. Appropriate reference (IUCN Seahorse, Pipefish and Stickleback Specialist Group (2019). https://www.iucn-seahorse.org (downloaded on 01/11/2019) has also been cited.

Query: Lines 63-64: the validity of H. montebelloensis and H. borboniensis is disputued by Lourie et al. (2016). Although it is not necessary to agree with this, it is necessary to make note in the manuscript.
Response: We have noted the point raised by the Reviewer. Accordingly, the sentence has been modified by including a caveat. Added a sentence ‘The validity of H. bornoinensis and H. montebelloensis as separate species however, is currently under revision due to their synonymity with H. kuda and H. zebra, respectively’ (Lourie et al., 2016).

Query: Lines 69-70: "(e.g. pelvic and caudal fins), high variability and overlapping of body proportions, colour (camouflage) and skin filaments" should read "(e.g. pelvic and caudal fins), and have high variability of and overlapping body proportions, colour

(camouflage) and skin filaments.

Response: As suggested by the Reviewer, the sentence has been modified.

Query: Line 73: 'relationships' rather than 'relationship'

Response: As suggested, ‘relationship’ changed to ‘relationships

Query: Lines 80-81: currently reads "However, instances of small migration of seahorses in search of proper habitat", should read "However, instances of limited migrations by seahorses in search of proper habitat

Response: As suggested, the statement has been modified.

Query: Lines 83-84: I would not consider oceanic current to be 'stepping stones'. Although currents facilitate dispersal, the real 'stepping stones' would be patches of habitat along the way.

Response: We agree with the Reviewer to a certain extent. Oceanic currents may facilitate long distance dispersal but indirectly facilitate stepping stone mode for short distance dispersal. Accordingly, the sentence has been altered.

Query: Lines 88-89: reference [35] (Aylesworth et al. 2016) report this species from western Thailand, not "from southeastern India to Korea and Japan"

Response: We are thankful to the Reviewer for pointing out this mistake. The statement has been now corrected in the revised manuscript.

Query: Lines 89-91: 'Previous reports' should include the Aylesworth et al (2016) observations from western Thailand. As stated currently, it appears the authors of this manuscript have extended the range from Vietnam to western India, when in fact the manuscript is only extending it from eastern India to western India. (see Figure 27 in Lourie et al. 2016).

Response: Agreed. As suggested by the Reviewer, the sentence has been corrected in the revised manuscript. Yes it is true that the reported new range in the study is from eastern India to western India.

Query: Lines 90-92: this species is not listed as Vulnerable because of 'recent exploitations'; it is listed as Vulnerable because of a suspected reduction in population size of >30% over the past 10 years due to fisheries exploitation.

Response: We totally agree with the Reviewer’s comment. As suggested, now the statement has been modified to convey the correct rationale for listing H. mohnikei as ‘VULNERABLE’

Query: Line 92: the word 'vulnerable' should be capitalized, as it is a formal IUCN Red List Category.

Response: In order to comply with the format of IUCN, the word ‘Vulnerable’ changed to ‘VULNERABLE’

Query: Line 98: there needs to be a bridge sentence: the current text indicates that surveys found no new specimens and then goes straight into the fact that more specimens were found. The fact that the specimens examined were caught as bycatch in bag fisheries needs to be explained here, prior to stating the expansion.

Response: We thank the Reviewer in pointing out this error. In order to have a smooth flow, a bridge sentence indicating the specimens examined were based on the incidental catch has been inserted in the revised manuscript (Lines, 111-114).
Query: Lines 101-102: how do we know that this is a recent expansion of the range? Presumably this species could have been present here all along without being detected.

Powered by Editorial Manager® and ProduXion Manager® from Aries Systems Corporation

Response: While it is true that this species could have been here without being detected all these years, but we are the first ones to report its occurrence along the west coast of India evidenced with both morphological and molecular data. Previous report of its occurrence from Indian waters by Thangaraj and Lipton (2007) is from southeastern coast of India and is solely based on the morphological data of a single specimen. As mentioned in the manuscript, intense sampling from coastal waters of west coast of India and comparison of H. mohnikei populations from Southeast Asian regions are required for comprehensive biogeography of this small seahorse species in the Indo-Pacific Region.

Query: Line 109: "The collection site of seahorses" - suggest rewording is "The locality at which the examined seahorse specimens were collected from"

Response: Corrected as suggested.

Query: Line 119: suggest deleting 'characterized by'

Response: Deleted

Query: Line 157: replace 'three time counting' with 'triplicate counting'

Response: As suggested, 'three time counting' changed to 'triplicate counting'

Query: Line 158: change 'were also taken in case of male specimens' to 'were measured in all male specimens'.

Response: Corrected as suggested by the Reviewer.

Query: Line 230: remove 'almost'

Response: Removed

Query: Lines 232-233: Are there two cheek spines and two eye spines in total, or on each side of the head (so four)?

Response: Two prominent cheek spines and double rounded spines below the eye (each on the either side of the head) were observed

Query: Table 1: header needs to be repeated at top of second page

Response: We are sorry for the formatting issue. The size of the Table 1 has now been adjusted so that all the data of the Table to appear on the singe page.

Query: Line 258: replace 'A comparative' with 'A comparison of' Line 260: change 'was' to 'were'

Query: Line 265: delete 'the'

Reply: Deleted.

Query: Line 266: it appears the heading for Table 2 became separated from the rest of the table.

Response: We are sorry for this. Table 2 has been reformatted and positioned to so that the heading and the data to appear on the single page.

Query: Line 266: replace 'distinguishes' with 'distinguish'

Query: Line 337: replace 'seahorses' with 'seahorse'

Response: ‘seahorses’ replaced with ‘seahorse

Query: Lines 359-370: Unless the authors actually collected field data regarding ocean circulation, this section should remain entirely in the methods section where the study

Powered by Editorial Manager® and ProduXion Manager® from Aries Systems Corporation

site is described.

Response: As suggested by the Reviewer, the Figure (Fig. 8) describing the ocean circulation has been moved to Materials & Methods section (now, Fig. 4). As mentioned in the manuscript, the basin-wide prevailing winter circulation (December–February) in the Indo-Pacific region based on the daily data for the 10 years (2007 to 2017) has been prepared and presented.

Query: Lines 386-388: should read "In northeast Asia, H. mohnikei is reported from China and South Korea".

Response: As suggested, the sentence has been corrected.

Query: Line 397: change 'morpho-molecular' to 'morphological and molecular'; delete 'is the most recent'

Response: As suggested, ‘morpho-molecular’ changed to ‘molecular and molecular’. Deleted ‘is the most recent’ in the revised manuscript.

Query: Line 398: delete 'distributional'

Response: Deleted as suggested

Query: Line 405: delete 'of' after 'constructed'

Query: Line 410: replace 'sequences' with 'sequence'

Response: Replaced ‘sequence’ with ‘sequences’

Query: Line 421: the term 'disjunct' is misused here. I don't believe there is any evidence to suggest any disjunctions in the population.

Response: We totally agree to the Reviewer’s view. In the revised manuscript, the term ‘disjunct’ has been removed.

Query: Lines 421-423: this statement needs some nuance. Genetic differences within a species do not necessarily mean that cryptic species exist. These difference can mean that there are cryptic species, but the authors should state what a defensible threshold for species delineation might be (i.e. at what % difference would we likely consider them separate species?).

Response:

Query: Line 425: replace 'on' with 'of' Line 431: replace 'report' with 'data'

Response: As suggested by the Reviewer, necessary edits have been incorporated in the revised manuscript

Query: Line 451: again I am not aware of any evidence to say that the population is disjunct

Response: We agree with the Reviewer and thank for pointing out this mistake. Now, the term ‘disjunct’ replaced with ‘geographically disconnected’

Query: Lines 474-475: replace 'attain a settlement phase (benthic)' with 'settle on the benthos'

Query: Line 476: the authors need to provide evidence that the population is disjunct

Response: Now, the term ‘disjunct’ replaced with ‘geographically disconnected’

Query: Line 484: delete 'a'; replace 'promoting' with 'permit'

Response: Necessary edits have been done.

Powered by Editorial Manager® and ProduXion Manager® from Aries Systems Corporation

Query: Line 490: delete 'as'

Response: Deleted

Query: Lines 488-500: Is there evidence that this species originated in China/Japan? This section reads as though the species dispersed here recently. How can we be sure it didn't originate in India and disperse to the east? The prevailing currents are only shown for one season.

Response: We thank the Reviewer for this interesting suggestion. There are no published evidence that this species originated in China/Japan but initial reported type specimens were from Japan/Vietnam and in recent years sighted/reported from other locations, as well. According to Teske et al possibility of originating H. mohnikei in India and dispersal to east is cannot be overruled but required supportive genetic data which we planning with more specimens and control region sequencing. We can also mention about this feature studies in conclusion

Query: Line 506: change 'entail seahorses to flow downstream' to 'force seahorses downstream'

Response: As suggested, necessary corrections have been incorporated in the revised manuscript

Query: Line 518: delete 'inhabiting'

Response: Deleted ‘ínhabiting'

Query: Line 520: it's not clear what the authors mean by 'translocated populations'.

Response: We are sorry for the usage of incorrect term here. Now, 'translocated' replaced with ‘geographically disconnected’

Query: Line 528: Translocation suggests that humans collected the seahorses elsewhere and relocated them to Goa. I don't think that is what this paper suggests - the evidence is clear that this is probably an extension of their natural range based on new information. I suggest deleting point (i). This sentence should state that these are either vagrants or a previously unreported established population.

Response: We are grateful to the Reviewer for pointing out this mistake and incorrect usage of the term, ‘translocation’ here. Concurring to the Reviewer’s suggestion, the point (i) has been deleted in the revised manuscript.

Reviewer #4:

General Comments

Comment: Mr. Rayadurga and colleagues are to be commended for their efforts to understand the taxonomic identity and biogeography of a rare and previously poorly documented seahorse species occurring on the central west coast of India. As the authors state, seahorses globally are in decline, driven by their demand as an ingredient in traditional Chinese medicine as well as by degradation of their coastal marine habitat by human activities. The taxonomy and distribution of seahorses must be further clarified if seahorse populations are to persist in the face of these pressures.

Response: We thank the Reviewer for positive feedback on the manuscript.

Comment: While indeed it is intriguing to find and identify several specimens of H. mohnikei in the Goa region, unfortunately the methods presented in this manuscript do not support the conclusions the authors draw, as explained below. In addition to the disconnect between the analyses and their conclusions, the findings of this paper are more appropriate for a more specialized journal, such as Marine Biodiversity Records or Zootaxa, than for PLoS ONE. The manuscript also needs a thorough editing by a

native English speaker for grammar and clarity of prose. For all of these reasons, as explained in more detail, I cannot recommend this manuscript be published in PLoS ONE.

Response: We appreciate the Reviewer for providing the constructive criticisms. Here, we provide the point by point rebuttal/clarifications sought by the reviewer. While we agree that one of the shortcomings could be small sample size, nevertheless, the limited dataset was robust enough to provide interesting insights into H. mohnikei—small and highly migratory seahorse species. As the Reviewer agree that it is quite hard to obtain sufficient number of seahorse specimens for the study due to their cryptic nature and low population density. The revised version of the manuscript is more organised and focussed after incorporating the suggestions and corrections made by all four reviewers and performing additional analysis. We chose PLoS ONE as a medium of publishing our results, firstly because of the multidisciplinary nature of the journal and secondly the 'Open Access Policy' of the journal—which would allow wider dissemination of the results, quickly. In addition, we have seen few similar papers of this nature published

Comment: While indeed it is likely that the 4 analyzed seahorse specimens are H. mohnikei, neither the morphological or the genetic analyses have been conducted in a way that support the authors conclusions.

Response: We thank the reviewer for constructive feedback. Following the Reviewer's comment, we have made two major changes to the manuscript which are explained point by point below.

Comment: For the morphometric analysis, it is essential to include one or preferably more vouchered specimens of H. mohnikei in the analysis to conclusively determine that the morphometric cluster analysis supports the identification of the specimens in question. By examining only the 4 specimens in question against specimens from two other distinct seahorse species, the authors have only demonstrated that the unidentified seahorse specimens are NOT H. kuda or H. trimaculatus. But this analysis cannot be used to conclude these unidentified specimens ARE H. mohnikei. For that, you would need to also measure one or more vouchered specimens of H. mohnikei and show that the Goa specimens cluster together with known H. mohnikei morphometric data.

Response: As suggested by the Reviewer, now a rigorous morphological and morphometric analysis has been conducted A comparative assessment of diagnostic morphological features and morphometric characters of Goa specimens with all vouchered specimens of Hippocampus mohnikei has been conducted. Results of comparative analysis has been tabulated under Table 3. This comparison demonstrated that the unidentified specimens from Goa are H. mohnikei as the most of the diagnostic morphological characters of Goa specimens matched with those of vouchered specimens.

Comment: The case with the genetic data is the same. The correct analysis would be to download ALL VOUCHERED H. mohnikei COI and cytb sequences, align them, conduct an analysis of the correct model of molecular evolution for this dataset (there is no justification for why the authors used K2P - how was it determined that this was indeed the correct evolutionary model for these data?), and conduct a rigorous phylogenetic analysis that assesses the position of the 4 Goa specimens among all other known H. mohnikei sequences for both genes.

By choosing only a few sequences from other H. mohnikei specimens to compare to, the authors are depriving this analysis of the dataset required to more fully understand the identity and phylogeographic relationships of the Goa specimens.

Response: We thank the Reviewer for pointing out this mistake. As suggested by the Reviewer, we have downloaded all vouchered H. mohnikei COI and Cyt b sequences from GenBank (S1 Table), aligned them and conducted a rigorous phylogenetic analysis. This rigorous analysis allowed to assess the phylogeographic analysis of 4 specimens for Goa among all other known H. mohnikei sequences for both COI and Cyt b genes.

Powered by Editorial Manager® and ProduXion Manager® from Aries Systems Corporation

	Comment: The results of the genetic analysis do not support the conclusions the authors make, but potentially there is something quite interesting to be investigated here. The authors claim that the taxonomic identity of the Goa specimens in confirmed because the sequences of the putative H. mohnikei from Goa are nearly identical to just 3 other H. mohnikei sequences from southeast Asia. And here is where it gets even more interesting, while also pointing to the lack of support for the authors conclusions. The pairwise genetic distances reported between the Goa specimens and the 3 SE Asia sequences for both Cytb and COI are ENORMOUS, and absolutely cannot reflect intraspecific genetic variation. The authors are reporting 3-10% distance in COI, where most valid species can be separated at the 3% level. The cytb differences are so large as to reflect saturation in mutations - you basically cannot get larger genetic distances than 18% or so, and levels that high almost always reflect completely different species. These results only validate the need to include all known vouchered specimens of H. mohnikei in the analysis, to better understand the clustering among the 100 or so available specimens in GenBank and the Barcode of Life databases.<br><br>There are several possible reasons for these findings: an inaccurate sequence alignment, the need for a more sophisticated model of molecular evolution that can account for the possibility of multiple mutations at a given site, or that the specimens from Goa really are so genetically distinct they have diverged significantly on their evolutionary pathway. A more rigorous genetic analysis is the only way to understand what these 4 mystery specimens really represent.<br><br>Response: Post-sequencing of remaining 3 Goa specimens for COI and Cyt b genes (now, total four) and aligning them with all vouchered downloaded H. mohnikei COI and Cyt b sequences from GenBank, the results of phylogenetic analysis has improved greatly. The revised manuscript has addressed most of the the apprehensions, particularly on the genetic distance raised by the Reviewer. The manuscript has been now checked and verified by Grammarly software for English language.<br><br>Comment: I recommend the authors shorten their paper to a scientific note format and publish this work in a more specialized journal such as those listed above. By publishing the sequences and placing them in the public record, they will have done a great service to adding important information regarding the continuing mystery of seahorse taxonomy and distributions, for which they will be acknowledged.<br><br>Response: We thank the Reviewer for this suggestion. Considering the sighting of H. mohnikei along the west coast of India has biogeographic and conservation significance, the manuscript attempts to address the probable reasons for its occurrence and range expansion. We wanted the manuscript to be quite different from routine Scientific Notes merely reporting the occurrence of this highly migratory small seahorse species. We hope this manuscript would provide new information and deeper insights which may be helpful in addressing the continuing mystery of seahorse taxonomy and distributions.
**Additional Information:**	
**Question**	**Response**
**Financial Disclosure**<br><br>Enter a financial disclosure statement that describes the sources of funding for the work included in this submission. Review the submission guidelines for detailed requirements. View published research articles from *PLOS ONE* for specific examples.<br><br>This statement is required for submission and **will appear in the published article** if	The author(s) received no specific funding for this work

Powered by Editorial Manager® and ProduXion Manager® from Aries Systems Corporation

the submission is accepted. Please make sure it is accurate.<br><br>**Unfunded studies**<br>Enter: *The author(s) received no specific funding for this work.*<br><br>**Funded studies**<br>Enter a statement with the following details:<br>• Initials of the authors who received each award<br>• Grant numbers awarded to each author<br>• The full name of each funder<br>• URL of each funder website<br>• Did the sponsors or funders play any role in the study design, data collection and analysis, decision to publish, or preparation of the manuscript?<br>• **NO** - Include this sentence at the end of your statement: *The funders had no role in study design, data collection and analysis, decision to publish, or preparation of the manuscript.*<br>• **YES** - Specify the role(s) played.<br><br>* typeset	
**Competing Interests**<br><br>Use the instructions below to enter a competing interest statement for this submission. On behalf of all authors, disclose any competing interests that could be perceived to bias this work—acknowledging all financial support and any other relevant financial or non-financial competing interests.<br><br>This statement **will appear in the published article** if the submission is accepted. Please make sure it is accurate. View published research articles from *PLOS ONE* for specific examples.	The authors have declared that no competing interests exist.

**NO authors have competing interests**<br><br>Enter: *The authors have declared that no competing interests exist.*<br><br>**Authors with competing interests**<br><br>Enter competing interest details beginning with this statement:<br><br>*I have read the journal's policy and the authors of this manuscript have the following competing interests: [insert competing interests here]*<br><br>* typeset	
**Ethics Statement**<br><br>Enter an ethics statement for this submission. This statement is required if the study involved:<br><br>• Human participants<br>• Human specimens or tissue<br>• Vertebrate animals or cephalopods<br>• Vertebrate embryos or tissues<br>• Field research<br><br>Write "N/A" if the submission does not require an ethics statement.<br><br>General guidance is provided below. Consult the submission guidelines for detailed instructions. **Make sure that all information entered here is included in the Methods section of the manuscript.**	Dead fishes landed as incidental catch in of bag nets by local fishermen formed the study material.

Powered by Editorial Manager® and ProduXion Manager® from Aries Systems Corporation

**Format for specific study types**<br><br>**Human Subject Research (involving human participants and/or tissue)**<br>• Give the name of the institutional review board or ethics committee that approved the study<br>• Include the approval number and/or a statement indicating approval of this research<br>• Indicate the form of consent obtained (written/oral) or the reason that consent was not obtained (e.g. the data were analyzed anonymously)<br><br>**Animal Research (involving vertebrate animals, embryos or tissues)**<br>• Provide the name of the Institutional Animal Care and Use Committee (IACUC) or other relevant ethics board that reviewed the study protocol, and indicate whether they approved this research or granted a formal waiver of ethical approval<br>• Include an approval number if one was obtained<br>• If the study involved *non-human primates*, add *additional details* about animal welfare and steps taken to ameliorate suffering<br>• If anesthesia, euthanasia, or any kind of animal sacrifice is part of the study, include briefly which substances and/or methods were applied<br><br>**Field Research**<br><br>Include the following details if this study involves the collection of plant, animal, or other materials from a natural setting:<br>• Field permit number<br>• Name of the institution or relevant body that granted permission	
**Data Availability**<br><br>Authors are required to make all data underlying the findings described fully available, without restriction, and from the time of publication. PLOS allows rare exceptions to address legal and ethical concerns. See the PLOS Data Policy and FAQ for detailed information.	Yes - all data are fully available without restriction

A Data Availability Statement describing where the data can be found is required at submission. Your answers to this question constitute the Data Availability Statement and **will be published in the article**, if accepted.<br><br>**Important:** Stating 'data available on request from the author' is not sufficient. If your data are only available upon request, select 'No' for the first question and explain your exceptional situation in the text box.<br><br>Do the authors confirm that all data underlying the findings described in their manuscript are fully available without restriction?	
**Describe where the data may be found in full sentences. If you are copying our sample text, replace any instances of XXX with the appropriate details.**<br><br>• If the data are **held or will be held in a public repository**, include URLs, accession numbers or DOIs. If this information will only be available after acceptance, indicate this by ticking the box below. For example: *All XXX files are available from the XXX database (accession number(s) XXX, XXX.*).<br>• If the data are all contained **within the manuscript and/or Supporting Information files**, enter the following: *All relevant data are within the manuscript and its Supporting Information files.*<br>• If neither of these applies but you are able to provide **details of access elsewhere**, with or without limitations, please do so. For example:<br><br>*Data cannot be shared publicly because of [XXX]. Data are available from the XXX Institutional Data Access / Ethics Committee (contact via XXX) for researchers who meet the criteria for access to confidential data.*<br><br>*The data underlying the results presented in the study are available from (include the name of the third party*	All relevant data are within the manuscript and its Supporting Information files.

and contact information or URL).<br>• This text is appropriate if the data are owned by a third party and authors do not have permission to share the data.<br><br>* typeset	
Additional data availability information:	

Powered by Editorial Manager® and ProduXion Manager® from Aries Systems Corporation

Manuscript Click here to access/download;Manuscript;Manuscript.docx

Morphological and molecular evidence for ~~range extension and first occurrence~~ of the Japanese seahorse, *Hippocampus mohnikei* (Bleeker 1853) in a bay-estuarine system of Goa, central west coast of India

Sushant Sanaye[1], Rakhee Khandeparker[1], Rayadurga Anantha Sreepada[1*], Mamatha Singanahalli Shivaramu[1], Harshada Kankonkar[1], Jayu Narvekar[2], Mukund Gauthankar[1]

[1] Aquaculture Laboratory, Biological Oceanography Division, CSIR-National Institute of Oceanography, Dona Paula, Goa–403 004, India

[2] Physical Oceanography Division, CSIR-National Institute of Oceanography, Dona Paula, Goa–403 004, India

* Corresponding author

E-mail: sreepada@nio.org (RAS)

Abstract

Accurate information of taxonomy and geographic range of seahorse species (genus *Hippocampus*) is the first step in preparing threat assessments and designing effective conservation measures. Here, we report ~~the range expansion and first occurrence~~ of the Japanese seahorse, *Hippocampus mohnikei* (Bleeker, 1853) from the Mandovi estuarine ecosystem of Goa, central west coast of India (CWCI) based on ~~morpho-~~molecular analyses. The morphometric and meristic traits particularly~~,~~ short snout (29–35% head length), double cheek spine, low coronet ~~and~~ long tail (51.2–57.9% of standard length), 11 trunk rings, 37–39 tail rings, 15–16 dorsal and 12–14 fin rays observed in four collected specimens matched with the reported key diagnostic morphological criteria of vouchered specimens of *H. mohnikei*. The seahorse mitochondrial cytochrome oxidase subunit I (COI) and cytochrome *b* (Cyt *b*) genes were partially sequenced for conclusive genetic identification of the species under study. Molecular analysis showed that all four individuals ~~of seahorse species~~ clustered together suggesting a monophyletic lineage. Using the maximum similarity with GenBank database, maximum likelihood network and subsequent morphological analysis, the identity of the collected seahorse species was reconfirmed as *H. mohnikei*. With this new report, the geographic range of *H. mohnikei* extended significantly to the west~~ward side~~ from its previously known range. This new sighting of *H. mohnikei* could indicate a long-distance dispersal facilitated by ~~due to~~ prevailing oceanic circulation in the Indo-Pacific region or increased habitat suitability in bay-estuarine systems of Goa, CWCI. Comparison of the pair-wise genetic distances (Kimura 2-parameter) based on COI and Cyt *b* sequences revealed that the specimens examined in this study are genetically closer to *H. mohnikei* populations from Vietnam and Thailand than they are to those in Japan and China. To test the hypothesis

whether *H. mohnikei* are vagrants or previously unreported established population, long-term inter-annual sampling and analyses are warranted.

Introduction

Seahorses (Syngnathiformes; Syngnathidae) belonging to the single genus, *Hippocampus* (Rafinesque, 1810) are a fascinating and remarkable group of fishes with their unusual body shape and their biology, with males incubating the fertilized eggs in a brood pouch [1]. They are small, cryptic, and sedentary marine fishes that occur worldwide in shallow temperate to tropical waters in a wide variety of habitats, including seagrass beds, estuaries, coral and rocky reefs and mangroves [2−4]. Their remarkable ability to camouflage with structurally complex habitats further reduces the risk of predation [5], while providing stealth for prey capture [6]. However, these biological traits often also make them ~~unnoticeable from coastal habitat. Due to their cryptic nature, their ability to camouflage and the sparse distribution, seahorses become almost unnoticeable and thus restricting the biologists for their identification in dense coastal marine habitats.~~

The primary step in preparing threat assessments and designing effective conservation of seahorse populations is highly dependent on the precise identification of individual species in and beyond known geographic distributions [7]. Out of 44 recognized seahorse species (Genus *Hippocampus*) throughout the world [8], *H. kelloggi*, *H. kuda*, *H. spinosissimus* and *H. trimaculatus* are common in Indian coastal marine waters [9,10]. In addition, sightings based on morphological identification of single specimens of *H. mohnikei* from ~~the~~ Palk Bay, southeastern India [11], *H. borboniensis* [12] and *H. montebelloensis* [13] from Gulf of Mannar, southeast coast of India and *H. camelopardalis* from Gulf of Kachchh, northwest coast of India [14] have been reported. Another seahorse species, *H. histrix* is also suspected to occur in Indian coastal waters [15]. The validity of *H.* ~~*bornoinensis*~~ and *H. montebelloensis*

as separate species however, is currently under revision due to their synonymity with *H. kuda* and *H. zebra*, respectively [16].

The utility of morphological traits commonly used in diagnosis of *Hippocampus* spp. is quite challenging as they lack certain key physical features (e.g. pelvic and caudal fins), and have high variability ~~of~~ ~~overlapping~~ body proportions, color (camouflage) and skin filaments, fin-ray and trunk-ring numbers ~~may vary greatly within any given-species and between species~~ [3,17]. On the other hand, molecular methods have proved helpful in resolving morphologically challenging seahorse taxonomy [16,18], phylogenetic relationships within genus~~,~~ *Hippocampus* [19,20], natural species boundaries [21−23] and genetic variability [22,24] of many seahorse species. An integrated approach ~~of combination of~~ morphological and genetic analyses [25] would aid in the management of demographically separate populations as independent units and allow international legal mechanisms and international agreements such as the convention on international trade in endangered species (CITES) to work effectively [26].

Generally, seahorses are sedentary ~~fish species~~ with small home range and patchy distribution in sheltered areas such as seagrasses and seaweeds [2,27,28]. However, instances of limited migrations by seahorses in search of proper habitat, food or holdfasts have been reported [28,29]. Evidences of long-distance dispersal of seahorse species via rafting (floating debris and seaweeds) [2,12,30] and subsequent colonization [21] have also been documented. The role of oceanic currents facilitating ~~in~~ long-distance dispersal and range expansion of many seahorse species has also been highlighted [31−36].

The Japanese ~~seahorse~~ or Lemur-tail seahorse, *Hippocampus mohnikei* (Bleeker, 1853) is a small coastal seahorse ~~species~~ (5–8 cm) inhabiting seagrasses, mangroves, oyster beds and mussel farms [37]. Original records indicated that the distribution of *H. mohnikei* is restricted or native to the coastal waters around Japan and Vietnam [17,38−40]. However, new records

of *H. mohnikei* from Cambodia, Malaysia, Thailand and Vietnam [37], along with recently published studies from southeastern India [11] and Singapore [41], have greatly expanded the known geographical range of *H. mohnikei* within Southeast Asia. As a consequence of suspected reduction in population size to the tune of >30% over the past 10 years due to fisheries exploitation [37,42−44], the status of *H. mohnikei* in the IUCN Red List of Threatened Species has been listed as 'VULNERABLE' [45].

A probable ~~occurrence~~ of *H. mohnikei* along the central west coast of India (CWCI) ~~began~~ in February 2017 when a fisherman posted a picture of seahorse incidentally caught in gill net operated in the Chapora estuary, Goa (India) (15.6120° N, 73.7506° E) on social media [S1 Fig] which was suspected to be an adult male of *H. mohnikei*. Sustained follow-up surveys for its occurrence in the surrounding environs of the Chapora estuary did not yield in any further ~~reporting~~. Subsequently, four sub-adult specimens of *H. mohnikei*—hitherto sighted only from southeast coast of India [11], caught as an incidental catch in bag net fisheries during July, 2018 in the Mandovi estuarine system, Goa,CWCI have been examined here.

This contribution describes the ~~range expansion and first occurrence~~ of *H. mohnikei* from the western coastal India. To test further the hypothesis that the morphologically identified seahorse species is *H. mohnikei*, two mitochondrial DNA gene regions (loci), cytochrome oxidase subunit I (COI) and cytochrome *b* (Cyt *b*) were partially sequenced for reconfirmation. Because of its rapid evolutionary rate and almost complete maternal inheritance, mitochondrial DNA (mtDNA) has been widely used to identify both population structure and genetic variability [46]. Within mtDNA, the COI and Cyt *b* genes are widely used for constructing phylogeny of many taxa including inter-specific and intra-specific variation in seahorse species owing to their slowest mutation and lower substitution rates compared to other mtDNA genes [16,19,25]. Potential role of ocean currents on the range

expansion of *H. mohnikei* to north-western Asia have also been discussed. In the wake of the vulnerability of seahorse populations to the threats such as habitat alteration/destruction and fishing pressure globally, the present sighting of *H. mohnikei* in a bay-estuarine system of CWCI is of considerable conservation and biogeographic significance.

Materials and Methods

Collection site and seahorses

The locality (Brittona, Goa, India, 15.3059°N, 73.5073°E, Fig 1) at which the examined seahorse specimens were collected is a shallow water littoral environment in the proximity to the mangrove-dominated Chorao Island in the Mandovi estuary, CWCI. The currents are largely tide dominated, the tides being semi-diurnal with a mean amplitude of 1.3 m. Currents are also influenced by the large seasonal freshwater influx [47] during the summer monsoon season (June to September). Temporal variations in abiotic and biotic factors influenced by monsoonal precipitation and seasonal upwelling render the collection location as one of the most ecologically complex ecosystems [48,49]. Significant lowering of salinity occurs when the freshwater discharge is at its maximum during monsoon. During the post- and pre-monsoon periods, the salinity at the surface and bottom remains high (~25 ppt). The bottom topography at the collection site is muddy and comprises mainly of silt and clay with rich organic matter content [50]. The collection site is reported to harbor rich and diverse fishery resources and its importance in supporting the life history stages of several marine teleosts has also been well-documented [51].

Present report is based on four specimens of *H. mohnikei* (females, MK1 and MK2; males, MK3 and MK4; Fig. 2. and Fig. 3) that were incidentally caught in bag nets attached to the fixed stakes (total length, 25 m; mouth length, 5 m; cod end mesh size, 10 mm) operated at the water depth of ~6 m in the Mandovi estuary during July 2018. Dead seahorses

landed as incidental catch in bag nets attached to the stakes operated by local fishermen in the estuarine system formed the study material. No live seahorses were specifically caught for the present study. Fin clips from these dead seahorses were used for molecular analysis. Since, the present study did not involve any experimentation with live specimens, the approval of the ethics committee for the usage of live animals for experiments is therefore inconsequential.

Fig 1. Sampling localities of *H. mohnikei*. A map showing major rivers and estuarine systems of Goa (central west coast of India). Location of a suspected adult male *H. mohnikei* in Chapora estuary (●); collection site of confirmed report of *H. mohnikei* in the Mandovi estuary (★).

Fig 2. Collected seahorses from Goa. Sub-adult specimens of *Hippocampus mohnikei* (Bleeker 1853) (females, MK1 and MK2; males, MK3 and MK4; NIO1015/19) from the Mandovi estuary, Goa, central west coast of India.

Fig 3. Close-up view of *H. mohnikei*. *Hippocampus mohnikei* (Bleeker 1853) (A-male; B-female) from the Mandovi estuary, Goa, central west coast of India (NIO1015/19).

Morphological analysis

Before subjecting them to a detailed analysis of morphological characteristics, seahorse specimens were carefully photographed (Nikon D7200, Japan) and sexed via observation of presence or absence of brood pouch. Morphometric measurements and meristic counts were carried out following standardized protocols [15,17,52]. Morphometric characters such as height (Ht, linear distance from the top of the head to the tip of the stretched tail), head length

(HL), trunk length (TrL), tail length (TaL) and standard length (SL = HL+TrL+TaL), were measured. Measurements of snout length (SnL), ratio of head/snout lengths (HL/SnL), snout depth (SnD), head depth (HD), pectoral fin base length (PL) and dorsal fin base length (DL) were also conducted. All measurements were taken with the help of digital caliper to 0.1 mm and repeated to ensure accuracy. Trunk depths on 4th (TD4) and 9th (TD9) trunk rings were measured. The presence/absence of spines and dermal appendages was also noted. Meristic characters such as the number of trunk rings (TrR), tail rings (TaR), dorsal fin rays (DF), pectoral fin rays (PF) and anal fin rays (AF) were counted under stereo zoom microscope (Olympus SZX7, Japan) and the values were confirmed by triplicate counting. Brood pouch dimensions were measured in all male specimens.

Morphological identification of the collected seahorse specimens from Goa was based on comparison of the salient morphometric and meristic characters with the reported diagnostic features for different seahorse species [15,17,25]. For further confirmation, the morphological data of Goa specimens was compared with all vouchered specimens of *H. mohnikei* [11,15,25,53,54]. The voucher specimen collected from Goa is deposited in the national marine biodiversity repository at CSIR-National Institute of Oceanography, Goa, India (deposition ID: NIO1015/19).

Statistical analysis

Morphological data of *H. mohnikei* and two other commonly occurring seahorse species (*H. kuda* and *H. trimaculatus*) in the coastal waters of India was evaluated by one-way analysis of variance (ANOVA) followed by the Duncans multiple-range test [55]. The level of significance was tested at 5.0%, represented as $P < 0.05$. A comparison of morphological characters and meristic features of *H. mohnikei* (n = 4) with *H. kuda* (n = 10) and *H. trimaculatus* (n = 10) was made through principal component analysis (PCA). PCA was

employed in which factor loadings based on components were used to determine the morphometric factors using PRIMER software (version 6) after square-root transformation.

Molecular analysis: DNA extraction, PCR amplification and sequencing

For gene-based phylogenetic analysis, fragments of dorsal fin from freshly landed dead seahorses were removed and preserved in 95% ethanol at –20°C until subjected to DNA extraction. DNA extraction was carried out as described by Kumar et al. with a few modifications [56]. In brief, lysis buffer and sodium dodecyl sulfate were added to the fin tissue and vortexed. Further, RNase and Proteinase K were added to the mixture and incubated for 15–20 minutes at room temperature. After the incubation period, the DNA was extracted using phenol:chloroform:isoamyl alcohol (25:24:1) mixture. Precipitated DNA was washed with 70% cold ethanol. The gel electrophoresis was performed to confirm the quality and integrity of DNA.

Seahorse mitochondrial cytochrome oxidase subunit I (COI) gene was amplified using primers, forward (5'TCAACTAATCACAAAGACATCGGCAC3′) and reverse (5'ACTTCGGGGTGCCCAAAGAATC3′) [57]. Similarly, mitochondrial cytochrome *b* (Cyt *b*) gene fragment was also amplified using PCR with seahorse-specific primers, forward (5'AACYAGGACYAATGRCTTGA3') and reverse (5′GCASWAGGGAGGRKTTTAAC3′) [43]. Reactions were carried out in a thermal cycler (Veriti® 96-well Thermal Cycler, Applied Biosystems, CA, USA). The amplification of COI and Cyt *b* genes was carried out by following cycling conditions as described by Lourie et al. with few modifications; an initial denaturation step of 95°C for 2 minutes followed by 39 cycles of 94°C for 30s, 55°C for 30s 72°C for 1 minute and a final extension at 72°C for 2 minutes [58]. Amplified PCR products of both COI and Cyt *b* genes were verified on agarose gel (1.5%) and purified for sequencing using PCR purification kit (Sigma Aldrich, USA). Gene sequence of the PCR

products was determined using a taq dye deoxy terminator cycle sequencing kit (PerkinElmer, CA, USA) following supplier's protocols. Sequencing reaction products were analyzed on 373 automated DNA Sequencer (Model 313 xl; Applied Biosystems, CA, USA). Amplification of the mitochondrial COI and Cyt *b* genes resulted in fragments of 609 bp and 639 bp, respectively.

Phylogenetic analysis

Nucleotide sequences generated for four specimens collected from Goa (MK1, MK2, MK3 and MK4) were deposited in GenBank under accession numbers MN595216, MN595218, MN595217 and MK330041.1 for COI and MN595213, MN595214, MN595215 and MK112274.2 for Cyt *b* genes, respectively). Sequence integrity and genetic affinity with known species was compared using the basic local alignment search tool (BLAST) of NCBI [59] (http://www.ncbi.nih.gov/BLAST). To complete the analysis, sequences of Goa specimens were aligned with 47 and 85 sequences of COI and and Cyt *b* genes, respectively of all vouchered specimens of *H. mohnikei* retrieved from the GenBank database (accession numbers shown in S1 Table). The mitochondrial COI and Cyt *b* gene sequences were aligned using the ClustalW algorithm implemented in MEGA 7 [60]. To ascertain stable and reliable relationships among the seahorse species, phylogenetic trees, separately for COI and Cyt *b* genes were constructed through maximum likelihood (ML) method using K2P distances. The analyses were performed in MEGA 7, and confidence levels were assessed using 1000 bootstrap replications. The alligator pipefish, *Syngnathoides biaculeatus* was used as an outgroup species. Genetic distance values are used to estimate intraspecies and interspecies kinships and also as a basis for phylogeny analysis. The degree of genetic divergence within *H. mohnikei* from different geographical locations (S1 Table) was assessed using Kimura 2-parameter (K2P) distance model [61] implemented in MEGA 7 [60]. Codon positions

included were 1st+2nd+3rd+noncoding. All ambiguous positions with alignment gaps and missing data were removed from the sequences (pair-wise deletion option).

Prevailing ocean circulation

In order to understand the basin-wide circulation in the Indo-Pacific region (encompassing South China Sea, East China Sea, and southeastern Bay of Bengal), monthly mean climatology was prepared based on daily data on U (zonal) and V (meridional) components of geostrophic current velocities derived from Aviso merged product for the years, 2007 – 2017. Altimeter products by Ssalto/Duacs and distributed by Aviso+, with support from Cnes (https://www.aviso.altimetry.fr) were used. The climatological currents generated for the winter season (December to February) from 2007 to 2017 for the Indo-Pacific region are depicted in Fig. 4.

Fig. 4. Oceanic circulation in Indo-Pacific region. Climatological surface currents during winter season (December–February) for the Indo-Pacific region. Red dots indicate the geographic locations of distribution of *Hippocampus mohnikei*. (WICC = West Indian Coastal Current, EICC = East India Coastal Current, NEC= North Equatorial Current and SCSWC = South China Sea Warm Current).

Results

Specimens examined

Colour of the head and body of freshly collected seahorse specimens (females, MK1 and MK2; males, MK3 and MK4; NIO1015/19) was dark brown to dark black. The entire body was covered with low spines and the coronet was observed to be short with five tiny

projections. Two prominent cheek spines and double rounded spines below the eye (each on the either side of the head) were discernible in all four specimens. (Fig. 2 and Fig. 3.).

Morphological taxonomy

Measured height (Ht) and standard length (SL) of specimens varied between 39.2 and 54.2 mm (48.2 ± 6.5 mm) and 47.3 and 60.1 mm (54.95 ± 5.48 mm), respectively. TaL was observed to be relatively long compared to the TrL of the body. The ratio of the head length (HL) to snout length (SnL) of all four specimens varied between 3.2 and 3.5 (Table 1). Number of trunk rings (TrR) in all specimens were 11 whereas, the number of tail rings (TaR) varied narrowly between 37 and 39. The number of TrR supporting dorsal fin was two and the number of TaR supporting dorsal fin was counted to be one in all specimens. Number of dorsal fin pectoral fin and anal fin rays recorded were 15–16, 12–14 and 4, respectively. Slight enlargements, respectively at 1st, 4th, 7th and $10/11^{th}$TrR and 4th, $7/8^{th}$, $9/10^{th}$, $13/14^{th}$TaR were prominently discernible (Table 1).

Comparative morphology

We compared the morphological features of *H. mohnikei* with other two commonly occurring seahorse species, *H. kuda* and *H. trimaculatus* occurring in coastal waters of India (Table 2). Fresh specimens of *H. kuda* was orange to dark brown with reddish spots over the body with single eye and cheek spine, rounded and backward curled coronet and deeper head and rounded or less developed body spines [15,16]. *H. trimaculatus* has three prominent black spots on the dorso-lateral surface of the first, fourth and seventh trunk rings [15,16] which are clearly absent in *H. mohnikei*. Single eye and hooked like cheek spine also observed in *H. trimaculatus*. Snout length (SnL) of *H. mohnikei* was shorter compared to the *H. kuda* and *H.*

trimaculatus. These morphological characters clearly distinguish *H. mohnikei* from *H. kuda* and *H. trimaculatus* (Table 2).

Table 1. Detailed morphology of *H. mohnikei*. Morphological characteristics of four specimens of *Hippocampus mohnikei* collected from the Mandovi estuary, Goa, central west coast of India (MK1 and MK2 = Female; MK3 and MK4 = Male).

	**MK1**	**MK2**	**MK3**	**MK4**
**Morphometric characters (mm)**				
Height (Ht)	39.2	54.2	48.1	51.3
Standard length (SL)	47.3	60.1	55.2	57.2
Trunk length (TrL)	16.1	19.4	15.2	15.1
Tail length (TaL)	24.2	32.1	31.2	33.1
Head length (HL)	8.5	9.1	9.2	8.7
Snout length (SnL)	2.5	3.1	3.3	2.5
HL/SnL ratio	3.4	3.2	3.3	3.5
Snout depth (SnD)	1.5	2.1	2.1	2.1
Head depth (HD)	5.5	7.1	6.9	7.2
Eye orbit	1.5	2.1	2.2	2.1
Trunk depth between 4th and 5th trunk ring (TD4)	3.1	4.2	3.9	4.1
Trunk depth between 9th and 10th trunk ring (TD9)	4.7	6.1	5.8	5.9
Pectoral fin base length	2.1	2.1	2.1	2.2
Dorsal fin base length	3.5	4.1	4.1	3.9
Anal fin base length	0.5	0.5	0.5	0.5
Pouch length	-	-	6.2	6.6
Pouch depth	-	-	3.1	2.5
Pouch width	-	-	3.2	3.3
**Meristic features (numbers)**				
Trunk Rings (TrR)	11	11	11	11
Tail Rings (TaR)	37	38	37	39
TrR supporting dorsal fin	2	2	2	2
TaR supporting dorsal fin	1	1	1	1
Cheek spines	2	2	2	2
Eye spines	2	2	2	2
Enlargement of trunk Rings	1, 4, 7, 11	1, 7, 10	1, 4, 7, 11	1, 4, 7, 10
Enlargement of tail Rings	4, 7, 10, 13	4, 8, 10, 13	4, 7, 9, 13	4, 7, 10, 14
Dorsal fin rays	15	16	16	16
Pectoral fin rays	12	14	14	14
Anal fin rays	4	4	4	4

Table 2. Morphological comparison of seahorses. Comparison of morphological characteristics of *H. mohnikei* with other two seahorse species, (*H. trimaculatus* and *H. kuda)*. Sample size for each species shown in parentheses. Mean±SD denoted with different superscripts significantly differ from each other ($P < 0.05$).

**Morphometrics (mm)**	***H. mohnikei* (*n* = 4)**	***H. kuda* (*n* = 10)**	***H. trimaculatus* (*n* = 10)**
Height (Ht)	48.2 ± 6.50^{a}	157.73 ± 19.24^{b}	93.06 ± 14.71^{c}
Standard Length (SL)	54.95 ± 5.48^{a}	182.65 ± 11.64^{b}	109.87 ± 6.45^{c}
Trunk Length (TrL)	16.45 ± 2.02^{a}	47.83 ± 2.54^{b}	26.40 ± 2.12^{c}
Tail Length (TaL)	30.15 ± 4.04^{a}	104.43 ± 9.07^{b}	61.70 ± 3.40^{c}
Head Length (HL)	8.83 ± 0.41^{a}	30.39 ± 1.88^{b}	21.70 ± 1.77^{c}
Snout Length (SnL)	2.67 ± 0.21^{a}	12.06 ± 1.04^{b}	8.90 ± 0.99^{c}
HL/SnL ratio	3.33 ± 0.14^{a}	2.54 ± 0.16^{b}	2.45 ± 0.19^{bc}
Snout Depth	1.95 ± 0.30^{a}	4.25 ± 0.41^{b}	3.45 ± 0.16^{c}
Head Depth	6.68 ± 0.79^{a}	14.80 ± 1.84^{b}	9.50 ± 0.53^{c}
TD4	3.83 ± 0.50^{a}	12.16 ± 1.19^{b}	6.75 ± 0.86^{c}
TD9	5.63 ± 0.63^{a}	18.68 ± 1.51^{b}	9.55 ± 0.90^{c}
Pectoral fin base length	2.03 ± 0.1^{a}	7.07 ± 1.2^{b}	4.75 ± 0.6^{c}
Dorsal fin base length	3.88 ± 0.3^{a}	15.73 ± 1.8^{b}	9.80 ± 0.8^{c}
**Meristic counts**			
Trunk Rings (TrR)	11 ± 0.00	11 ± 0.00	11 ± 0.00
Tail Rings (TaR)	38 ± 1.00^{a}	36 ± 1.00^{b}	41 ± 1.00^{c}
TrR supporting Dorsal Fin	2 ± 0.00	2 ± 0.00	2 ± 0.00
TaR supporting Dorsal Fin	1 ± 0.00	1 ± 0.00	1 ± 0.00
Cheek Spines	2 ± 0.00^{a}	1 ± 0.00^{b}	1 ± 0.00^{c}
Eye spines	2 ± 0.00^{a}	1 ± 0.00^{b}	1 ± 0.00^{c}
Dorsal fin rays	16 ± 1.00^{a}	18 ± 1.00^{b}	20 ± 1.00^{c}
Pectoral fin rays	14 ± 1.00^{a}	16 ± 1.00^{b}	17 ± 1.00^{c}

PCA used to elucidate the factors contributing to morphological differences in *H. mohnikei*, *H. kuda* and *H. trimaculatus* showed that many morphometric and meristic characters play an important role in differentiating ~~the seahorse~~ species. Results of PCA

analysis revealed that 11 morphometric and meristic characters integrated into two principal components (PC1 and PC2) which cumulatively contributed to 97% of total variance (S2 Table). The PC1 which explained 81% of total variation consisted of morphometric characters such as SL, TaL, TD4, HL, TD9, TrL, HD, SnL and SnD while, the PC2 which explained 16% of total variation consisted of the TaR and DF. The derived ordination diagram after plugging standardized morphological data of all individuals of three seahorse species into PCA is depicted in Fig. 5. PCA ordination diagram showed that three seahorse species were clustered out distinctly in orientation space. Therefore, based on comparison of morphological traits [15,16,25] and the results of PCA, the identity of seahorse specimens collected from Goa was confirmed as *H. mohnikei*.

Fig. 5. PCA analysis scores. Scores of factor loadings plot of principal component analysis of three species, *H. mohnikei*, *H. kuda* and *H. trimaculatus* based on morphological data.

Morphological measurements revealed that all the four Goa specimens are consistent and congruent with the salient morphological descriptions for type specimens of *H. mohnikei* reported by Lourie et al. [15,17,25] for specimens collected from Vietnam and Japan. Furthermore, meristic, morphometric, and key diagnostic morphological character comparisons from vouchered specimens of *H. mohnikei* [11,15,25,53,54], reconfirmed the identity of seahorse specimens collected from Goa as *H. mohnikei* (Table 3).

Table 3. Comparison of morphological characters. Comparison of diagnostic morphological characters (range and mean) of vouchered specimens of *Hippocampus mohnikei.* Values in parentheses are mean.

**Data source**	**Present Study**	**Lourie et al. [15,25]**	**Thangaraj and Lipton [11]**	**Zhang et al. [53]**	**Wibwo et al. [54]**
Voucher/field Number	NIO1015/19	RMNH7259	CMFRI No. 146	SCSMBC00741 8−19	KAUM-1. 17724
Locality	Goa, west coast of India	Vietnam	Southeast coast of India	Yellow Sea, China	Kagoshima, Southern Japan
No. of samples (N)	04	04	01	16	01
**Morphometrics (mm)**					
Standard Length (SL)	47.3–60.1 (54.95)	54.0–57.0	70.0	52.5–83.0 (66.4)	-
Trunk Length (TrL)	15.1–19.4 (16.45)		22.0	15.0–23.0 (19.2)	-
Tail Length (TaL)	24.2–33.1 (30.15)		38.0	28.5–46.0 (36.7)	-
Head Length (HL)	8.5–9.2 (8.83)		10.0	9.0–14.0 (10.5)	-
Snout Length (SnL)	2.5–3.3 (2.67)		3.0	1.8–3.9 (2.4)	
HL/SnL ratio	3.2–3.5 (3.35)	2.8–3.9 (3.0)	3.33	3.59–5.75 (4.5)	3.1
Snout Depth	1.5–2.1 (1.95)	-	2.5	-	-
Head Depth	5.5–7.2 (6.68)	-	7.0	-	-
TD4	3.1–4.2 (3.83)	-	5.5	-	-
TD9	4.7–6.1 (5.63)	-	8.0	-	-
**Meristic counts**					
Trunk Rings (TrR)	11	11	11	11	11
Tail Rings (TaR)	3737–39 (38)	37–40 (38)	38	37–40 (38)	38
TrR supporting dorsal fin	2	2	-	-	-
TaR supporting dorsal fin	1	1	-	-	-
Cheek Spines	2	2	2		2
Rounded spines below eye	Double	Double	Single	-	-
Dorsal fin rays (DF)	15–16	15–16	14	16	15
Pectoral fin rays	12–14	12–14 (13)	12	12–14 (13)	12
Anal fin rays (AF)	4	-	-	4	-

Phylogenetic analysis and genetic distance

PCR for the mitochondrial COI and Cyt *b* genes of Goa seahorse specimens resulted in 609 bp and 639 bp amplicons, respectively. Maximum likelihood (ML) phylogenetic analysis were performed using K2P distances with sequences of COI and Cyt *b* genes of seahorse specimens from Goa (MK1, MK2, MK3 and MK4) with all downloaded sequences of vouchered *H. mohnikei* from GenBank (S2 Table). ML trees of Goa seahorse specimens (GenBank accession numbers, MK330041.1, MN595216.1, MN595217.1 and MN595218.1 for COI gene; GenBank accession numbers, MK112274.2, MN595213.1, MN595214.1 and MN595215.1 for Cyt *b* gene) showed that all four individuals clustered together suggesting a monophyletic lineage (Fig. 6 and Fig. 7). Furthermore, sequences of COI and Cyt *b* genes of Goa specimens, respectively showed maximum phylogenetic similarities with *H. mohnikei* (GenBank accession number: GQ502157.1) ~~and with *H. mohnikei*~~ (GenBank accession numbers: EU179923.1 and EU179924.1) strongly supported by high bootstrap values of >95% (Fig. 6 and Fig. 7).

Fig. 6. Phylogenetic relationships of Goa specimens with other seahorse species based on COI gene sequences. ML phylogenetic tree of Goa specimens and all vouchered *Hippocampus mohnikei* based on mitochondrial cytochrome oxidase subunit I gene (COI) sequences using K2P distances with 1000 bootstrap iterations. Numbers at nodes indicate bootstrap support values and nodes with >50% bootstrap support values are shown. The alligator pipefish, *Syngnathoides biaculeatus* (KY066152.1) served as an out-group species. Scale bar = genetic distance of 0.05. ML = Maximum likelihood

Fig. 7. Phylogenetic relationships of Goa specimens with other seahorse species based on Cyt *b* gene sequences. ML phylogenetic tree of Goa specimens and all vouchered *Hippocampus mohnikei* based on mitochondrial cytochrome *b* gene (Cyt *b*) sequences using K2P distances with 1000 bootstrap iterations. Numbers at nodes indicate bootstrap support values and nodes with >50% bootstrap support values are shown. The alligator pipefish, *Syngnathoides biaculeatus* (EF189167.1) served as an out-group species. Scale bar = genetic distance of 0.05. ML = Maximum likelihood

Genetic distance describes the kinship distance ~~between intraspecies and interspecies~~. Calculated pair-wise genetic distance (K2P) values based on COI and Cyt *b* sequences of *H. mohnikei* from Goa and other localities (Japan, Korea, China, Vietnam and Thailand) revealed that *H. mohnikei* from Goa the show least genetic distances of 0.7% for both COI and Cyt *b* genes with populations of *H. mohnikei* from Vietnam (COI) and Thailand (Cyt *b*), respectively (Table 4 and Table 5). In contrast, Goa specimens showed highest genetic distances of 1% for COI and 7% for Cyt *b* genes with populations of *H. mohnikei* from China and Japan, respectively.

Table 4. Pair-wise genetic distance based on COI gene sequences. Pair-wise genetic distances (Kimura 2-parameter model) between *Hippocampus mohnikei* populations of Goa and the Indo-Pacific region based on COI gene sequences. Values in parentheses indicate percentage.

**Localities of *H. mohnikei* with GenBank accession numbers**	**Goa**	**Japan**	**Vietnam**	**China**
Goa Specimens (MN595216−MN595218; MK330041.1)	0.00			
Japan (GQ502158.1−GQ502159)	0.008 (0.8)	0.00		
Vietnam (GQ502157.1)	0.007 (0.7)	0.009 (0.9)	0.00	
China (KP140138−KP140181)	0.010 (1.0)	0.003 (0.3)	0.010 (1.0)	0.00

Table 5. Pair-wise genetic distance based on Cyt *b* gene sequences. Pair-wise genetic distances (Kimura 2-parameter model) between *Hippocampus mohnikei* populations of Goa and the Indo-Pacific region based on Cyt *b* gene sequences. Genetic divergence of > 2% within same locality separated out. Values in parentheses indicate percentage.

**Localities of *H. mohnikei* with GenBank Accession numbers**	**Goa**	**Japan1**	**Japan2**	**Korea1**	**Korea2**	**China**	**Thailand**
Goa Specimens (MN595213.1, MN595214.1, MN595215.1 and MK112274.2)	0.00						
Japan1 (AF192688.1–AF192689.1; KT731898.1–KT731909.1)	0.067 (6.7)	0.00					
Japan2 (KX017612.1–KX017625.1)	0.070 (7.0)	0.071 (7.1)	0.00				
Korea1 (KT731897.1–KT731920.1; KT731924.1–KT731926.1; KT731929.1; KT731932.1–KT731935.1; KT731940.1–KT731941.1; KT731944.1–KT731952.1 and KT731955.1)	0.066 (6.6)	0.040 (4.0)	0.044 (4.4)	0.00			
Korea2 (KT731921.1–KT731923.1; KT731928.1; KT731930.1–KT731931.1; KT731936.1–KT731937.1, KT731943.1 and KT731955.1)	0.067 (6.7)	0.004 (0.4)	0.012 (1.2)	0.066 (6.6)	0.00		
China (KT946929.1–KT946937.1; KC527556.1 and KC527584.1)	0.066 (6.6)	0.037 (3.7)	0.042 (4.2)	0.057 (5.7)	0.004 (0.4)	0.00	
Thailand (EU179923.1–EU179924.1)	0.007 (0.7)	0.001 (0.1)	0.001 (0.1)	0.001 (0.1)	0.001 (0.1)	0.001 (0.1)	0.00

Ocean circulation

As seen from Fig. 4 that the circulation in the Indo-Pacific region shows a large-scale flow moving from the East China Sea to the South China Sea. From the South China Sea, the major part of the flow turns southeastward entering into the Java Sea and a small part turns northwestward to eastern Bay of Bengal (BoB) and the Andaman Sea through Malacca Strait. From eastern BoB, the surface waters flow westward as a part of the north equatorial current (NEC). On encountering the coast of Sri Lanka, a part of the NEC move northward into the northern BoB, while the rest moves westward and into the southern Arabian Sea, south of Sri Lanka. The NEC feeds into the wintertime west India coastal current (WICC) and moves waters from NEC towards north into the Arabian Sea along its eastern boundary.

Discussion

The geographic range of *Hippocampus mohnikei* is still unresolved as Lourie et al [15,16] confirmed the nativity of this species to Japan and Vietnam and likely extending throughout the South China Sea and the Gulf of Thailand [16,17,25]. New records on the occurrence of *H. mohnikei* from many other non-native locations in north- and south-eastern coastal waters of Asia [11,37,41,44,57,62] suggest its significant extended distribution in the Indo-Pacific region (Fig. 8). ~~The sighting~~ of *H. mohnikei* in the Palk Bay, southeast coast of India [11] is ~~till~~ date the known range limit towards ~~northwestern Asian coastal waters~~. In southeastern Asian coastal waters, *H. mohnikei* has been reported from Singapore [41] as well as from Cambodia, Malaysia, Thailand and Vietnam [37]. In northeast Asia, *H. mohnikei* is reported from China [44,57] and South Korea [62,63].

Fig. 8. Reported sightings of *H. mohnikei* in Indo-Pacific region. Map showing native range, recent sightings and all reported sightings for *Hippocampus mohnikei* in the Indo-Pacific region. Sightings are classified as new (this study, ★), recently published (2004 – 2016, (▲) and original records [15] (*). Map was modified from Aylesworth et al. [37] and re-created in the World Geodetic System (WGS) 84 datum.

The present report on the occurrence of *H. mohnikei* in a bay-estuarine system of Goa, CWCI as confirmed by both morphological and molecular analysis represents a significant westward expansion compared to the previously known geographic range of this species [37]. Since the reporting of *H. mohnikei* solely based on morphological characters from the Palk Bay, southeast coast of India [11], no further sightings of this species have been reported from coastal marine waters of India. Therefore, a comparison of genetically distinct populations of *H. mohnikei* inhabiting native (Japan and Vietnam) and extended ranges in the Indo-Pacific region (Fig. 8) with Goa specimens gains significance. Lourie et al. reported that a Vietnamese specimen of *H. mohnikei* was genetically distinct from *H. mohnikei* from Japan [16,25].

Maximum likelihood (ML) tree constructed of Goa specimens with all vouchered specimens of *H. mohnikei* based on COI gene using K2P distances showed maximum phylogenetic proximity with specimen from Vietnam (GenBank accession number, GQ502157.1) (Fig. 6). On the other hand, ML tree constructed with sequences of Cyt *b* gene of Goa specimens showed maximum genetic similarities with *H. mohnikei* from Thailand (GenBank accession numbers, EU179923.1 and EU179924.1) (Fig. 7). In both ML trees of COI and Cyt *b* genes, phylogenetic proximity of all four Goa individuals with >95% bootstrap support thus confirming the taxonomic identity of Goa specimens as *H. mohnikei*. Furthermore, a comparison of pair-wise genetic distance values (K2P) based on sequence COI (Table 5) and Cyt *b* (Table 6) genes showed that Goa specimens showed least genetic

distance with populations of *H. mohnikei* from Vietnam (K2P value, 0.7%) and Thailand (K2P value, 0.7%), respectively. In contrast, Goa specimens showed highest genetic distances of 1% for COI and 7% for Cyt *b* genes with populations of *H. mohnikei* from China and Japan, respectively. Similar high pair-wise K2P values of sequences of Cyt *b* between *H. mohnikei* populations from the Gulf of Thailand and those from China (13.54−14.46%) and Japan (14.42−14.79%) have been recently reported [64].

A comparative assessment of K2P values of the present study with those from Vietnam [65] and Gulf of Thailand [64], respectively for COI and Cyt *b* suggest that *H. mohnikei* populations from Goa, Vietnam and the Gulf of Thailand might share a similar genetic identity and genetically distinct of those from China and Japan. Also, a possible genetic differentiation in populations of *H. mohnikei* inhabiting coastal marine waters of northeastern Asia (Japan, Korea and China) and southeastern Asia (Vietnam, Thailand, Cambodia, Malaysia, Singapore and India) may not be ruled out. Furthermore, least genetic divergence recorded between different populations of *H. mohnikei* (Goa vs Vietnam, COI gene) and (Goa vs. Thailand, Cyt *b* gene), the possibility of crypticity within *H. mohnikei* populations from these localities is ruled out by applying 2% threshold genetic divergence for assessing intra-specific differences [16]. In contrast, recent study by Panithanarak et al observed high genetic variance between the populations of *H. mohnikei* from the Gulf of Thailand and China (13.54–14.46%) and Japan (14.42–14.79%) suggested the possibility of cryptic species within *H. mohnikei* population range [64]. Further studies, however are necessary to assess the genetic variation within *H. mohnikei* populations in the entire Indo-Pacific region.

Recent studies have advanced our understanding of habitat associations of *H. mohnikei* compared to what has been described in the IUCN Red List of Assessment [45]. Previously, *H. mohnikei* was reported only in association with seagrass beds in Japan and in an estuary in Vietnam [16,25]. Association of *H. mohnikei* populations with seagrass beds (*Zostera* spp.) in

Korea [62,63], Japan [66] and China [44] has been reported. Whereas the specimen reported from ~~the~~ Palk Bay, southeastern India [11] was associated with two other additional genera of seagrass species (*Halophila* and *Thalassia*). Recently, published data on the habitat preferences of *H. mohnikei* from Southeast Asia [37] indicate its association with seagrass beds of *Cymodocea* spp., *Enhalus* spp., *Halodule* spp., *Halophila* spp., *Syringodium* spp. and *Thalassia* spp. Furthermore, these authors also report the association of *H. mohnikei* with mangroves, oyster beds, mussel farms and sandy beaches, whereas, Lim reported its occurrence in association with seaweeds in Singapore [41]. From these studies, it is evident that *H. mohnikei* occupies a wide variety of habitats.

All previous reports on the habitat descriptions of *H. mohnikei* were located in the coastal waters and bay-estuarine systems in the depth range of 0–10 m [11,17,25,37,44,62]. Interestingly, the occurrence of small juveniles of *H. mohnikei* in association with highly dense live prey organisms in the offshore waters of Japan has also been reported [39]. The present report of *H. mohnikei* is based on specimens caught as an incidental catch in bag nets fixed to the stakes operated in the Mandovi estuary, Goa. Although precise information on the habitat of *H. mohnikei*, could not be ascertained, it is worth describing the surrounding environs at the site of collection. Predominantly, the estuarine stretches of the River Mandovi have dense mangrove coverage and extensive oyster beds [67] and patches of seagrass, *Halophila ovalis* and *H. beccarii* [68]. The channel network of Mandovi estuary is characterized by islands (Chorao, Divar and Vanxim) and many sheltered areas interspersed with dense mangrove ecosystem. The present occurrence of *H. mohnikei* in a bay-estuarine system of Goa is not quite surprising as these are the typical habitats where this species has been previously reported. For ascertaining habitat shift by geographically disconnected populations of *H. mohnikei*, further intense studies on habitat specificities of different life stages therefore, are warranted.

Food availability has been considered as one of the factors influencing the abundance and distribution of seahorses [20]. Zooplankton such as *Oithona davisae*, Calanoida and *Penilia avirostris* have been identified as the main prey organisms for juvenile and young *H. mohnikei* in Tokyo Bay [39], whereas the adults have been reported to prey upon mainly larger organisms such as gammarid and caprellid amphipods [63]. Nutrient-rich waters of the Mandovi estuarine system sustain high primary productivity and abundance of zooplanktonic prey organisms such as copepods [69−71] which are essential for the survival and establishment of seahorse populations. Considering the prevailing environmental conditions, mangrove shelter and abundance of prey organisms, the likelihood of establishment and colonization of seahorse populations in these sheltered areas ~~is quite~~ high.

Factors such as habitat, diet, and even anthropogenic activities are the main reasons cited for the migration of seahorses [72]. Furthermore, instances of seahorses for long distance dispersal by means of rafting along with seaweeds or any other suitable holdfast under the influence of ocean currents during juvenile stages have been documented [2,22]. A southward ~~migration~~ of *H. histrix* ~1800 km under the influence of East Australian Current in the Great Barrier Reef has been reported [33]. Furthermore, ~1100 km migration from the south to the northeast coast of India by *H. fuscus* under the influence of East India Coastal Current has been also reported [35]. A Direct evidence for passive long-distance migration of *H. trimaculatus* at Malacca Strait has been documented recently [36].

Newborn juveniles of many seahorse species undergo a pelagic phase (planktonic) ranging between two and six weeks depending on the species before they settle down into sessile habitats (benthos) [2,73]. Consequently, planktonic phase juveniles are likely to facilitate in widespread gene flow across geographically disconnected populations and resulting in genetic homogeneity [74]. The occurrence of small juveniles of *H. mohnikei* (15.1−45.5 mm) in association with highly dense live prey organisms in offshore waters of

Japan [39] suggests that *H. mohnikei* might undergo a prolonged planktonic phase. Furthermore, a seasonal (warm and cold season) inshore-offshore migration by *H. mohnikei* populations inhabiting Chinese coast has been confirmed [57]. Higher dispersal capabilities of seahorse juveniles with floating debris, prey swirls and with seaweed rafts than adults has been reported [2,30,36]. It has been postulated that marine species with greater longevity, free swimming and feeding (planktotrophic) larval phases have relatively high dispersal abilities and thus permit genetic exchange between populations [75]. A relatively prolonged planktonic phase as in case of *H. mohnikei* may entail higher degree of dispersal under the influence of oceanic circulation.

The confirmed occurrence of four sub-adult specimens (Mandovi estuary) and suspected occurrence of an adult specimen of *H. mohnikei* in the proximately adjacent bay-estuarine ecosystem (Chapora estuary) of Goa, CWCI could be a consequence of either long-distance dispersal from Southeast Asian countries or stepping-stone mediated dispersal as revealed by oceanic currents circulation in the Indo-Pacific region. It is clearly seen from Fig. 4 that water mass from southeastern Asian coasts and east coast of India enters into the Arabian Sea as a result of winter circulation [76]. In the absence of geographical barriers between *H. mohnikei* populations of Goa and Southeast Asian countries, ocean currents flowing from regions where sightings of *H. mohnikei* may contribute to wide-spread dispersal. Therefore, it is plausible that oceanic currents may be aiding long-distance or stepping stone dispersal of *H. mohnikei* populations from different geographical locations such as Cambodia, Malaysia, Thailand and Vietnam [15,16,37,64], Singapore [41] and the Palk Bay, southeast coast of India [11] to the west coast of India.

Conversation with local fishermen who operate stake nets at the collection site revealed that sightings of seahorses are relatively more common during summer monsoon season than in non-monsoonal months. During the summer monsoon period, a number of rivulets and

rivers connected to the Mandovi estuarine network discharge large amounts of freshwater [77]. Consequently, it is possible that the rainwater discharge from sheltered areas—the preferred seahorse habitats may force seahorses flow downstream along with currents due to their poor swimming abilities.

It is well known that the west coast of India comes under the influence of substantial coastal cold-water upwelling event during the summer monsoon season [48,77]. It has been reported that the survival and dispersal of tropical marine species are known to be impacted by a drastic decrease in water temperature associated with upwelling [78−80]. Consequently, seahorse populations in western coastal India might undergo seasonal fluctuations [22]. A significant ~~decrement~~ in abundance and diversity of demersal fishes during the summer monsoon in a bay-estuarine system of the Mandovi river has been documented [51]. As a consequence of the reduction in salinity and drop in temperature associated with monsoonal precipitation and upwelling, respectively the seahorse populations might face limitations in growth and recruitment along the west coast of India. Interestingly, unlike most subtropical seahorse species, the greater tolerance by native populations of *H. mohnikei* to cold water in the Western Pacific has been reported [31]. However, further temperature stress simulation studies are required to ascertain whether geographically disconnected populations of *H. mohnikei* also possess a similar trait or not.

Conclusions

Based on the new information presented in this paper, there is clear evidence that *H. mohniei* has expanded its natural and reported geographic range. Recognizing the potential of seahorses as a popular flagship species in estuarine conservation and sustainable management [1,81,82], significant westward range expansion of *H. mohnikei* is of considerable biogeographic and conservation importance. Intriguingly, the possibility of *H. mohnikei*

originating in India and subsequent dispersal to the east cannot be ruled out. Although there is no published evidence to show that *H. mohnikei* originated in Japan/China, the original type specimens studied were however, from Japan and Vietnam. At the same time, it may be plausible that *H. mohnikei* might be present in other localities, but has not been reported or overlooked. Further intense sampling and rigorous molecular analysis of *H. mohnikei* populations along the Indian coast would be therefore required to confirm whether these are vagrants or a previously unreported fairly established population.

Acknowledgements

The authors are grateful to Prof. Sunil Kumar Singh, Director, CSIR-National Institute of Oceanography, Goa (India) for encouragement and facilities. Thanks are due to local fishermen Mr. Harish Halarnkar for providing seahorse specimens. Thanks are also due to Dr. Mani Murali and Mr. Devanand Kavlekar for their help in preparing the maps. We would like to deeply thank Dr. Qiang Lin for sharing the morphometric data of *Hippocampus mohnikei* for comparison. The authors are thankful to Dr. Graham Short and Riley Pollom and two anonymous reviewers for their critical and constructive comments for improving the quality and presentation of the manuscript. This represents contribution No. xxxx of the CSIR-National Institute of Oceanography, Goa (India).

Author Contributions

Conceived and designed the experiments, writing-original draft: SVS, RAS, RDSK; Performed the experiments: SVS, MSS, HTK, MG; Data curation and formal analysis: SVS, RAS, MSS, RDSK, JN; Contributed reagents/materials/analysis tools: JN, HTK, MG; Visualization, Writing - review & editing: RAS, SVS, RDSK.

References

1. Sreepada RA, Desai UM, Naik S. The plight of Indian seahorses: Need for conservation and management. Curr Sci 2002; 82: 377–378.
2. Foster SJ, Vincent ACJ. Life history and ecology of seahorses: implications for conservation and management. J Fish Biol 2004; 65: 1–61.
3. Kuiter RH. Seahorses and Their Relatives. Aquatic Photographics, Seaford, Australia; 2009.
4. Froese R, Pauly D. FishBase.www.fishbase.org. Downloaded on 10/2018.
5. Harasti D, Martin-Smith K, Gladstone W. Ontogenetic and sex-based differences in habitat preferences and site fidelity of White's seahorse *Hippocampus whitei*. J Fish Biol 2014; 85: 1413–1428.
6. Hellyer C, Harasti D, Poore A. Manipulating artificial habitats to benefit seahorses in Sydney Harbour, Australia. Aquat Conserv Mar Fresh Ecosyst 2011; 21: 582–589.
7. Short G, Harasti D, Hamilton H. *Hippocampus whitei* Bleeker, 1855, a senior synonym of the southern Queensland seahorse *H. procerus* Kuiter, 2001: molecular and morphological evidence (Teleostei, Syngnathidae). ZooKeys 2019; 824: 109–133.
8. IUCN Seahorse, Pipefish and Stickleback Specialist Group (2019). https://www.iucn-seahorse.org (downloaded on 01/11/2019).
9. Salin KR, Mohankumaran NC. Resources and biodiversity of seahorses and the need for their conservation in India. Aquacult Asia 2006; 10: 3–8.
10. Murugan A, Dhanya S, Rajagopal S, Balasubramanian T. Seahorses and pipefishes of the Tamil Nadu coast. Curr Sci 2008; 95: 253–260.
11. Thangaraj M, Lipton AP. Occurrence of the Japanese seahorse *Hippocampus mohnikei* Bleeker 1854 from the Palk Bay coast of southeastern India. J Fish Biol 2007; 70: 310–312.

12. Krishnan TS, Kuiter RH, Patterson J. Occurrence of western Indian Ocean seahorse, *Hippocampus borboniensis* Dumeril, 1870, in the Gulf of Mannar, Southeastern India. Ind J Geo-Mar Sci 2011; 40: 642–644.
13. Yogeshkumar JS, Geetha S. A new species of *Hippocampus montebelloensis* (Family: Syngnathidae) from the southeast coast of India. Int J Plant Anim Env Sci 2012; 2: 56–58.
14. Subburaman S, Murugan A, Goutham S, Kaul R, Prem Jyothi PVR, Balasubramanian T. First distributional record of giraffe seahorse, *Hippocampus camelopardalis* Bianconi 1854 (Family Syngnathidae) from Gulf of Kachchh waters, north west coast of India. Indian J Geo-Mar Sci 2014; 43: 408–411.
15. Lourie SA, Foster SJ, Cooper EWT, Vincent ACJ. A Guide to the Identification of Seahorses. Project Seahorse and TRAFFIC North America; 2004.
16. Lourie SA, ~~Pollam~~ RA, Foster SJ. A global revision of the seahorses *Hippocampus* Rafinesque 1810 (Actinopterygii: Syngnathiformes): taxonomy and biogeography with recommendations for further research. Zootaxa 2016; 4146: 1–66.
17. Lourie SA, Vincent ACJ, Hall HJ. Seahorses: an identification guide to the world's species and their conservation. Project seahorse, London, UK; 1999.
18. Silveira RB, Siccha-Ramirez R, Silva JRS, Oliveira C. Morphological and molecular evidence for the occurrence of three *Hippocampus* species (Teleostei: Syngnathidae) in Brazil. Zootaxa 2014; 3861: 317–332.
19. Casey SP, Hall HJ, Stanley HF, Vincent ACJ. The origin and evolution of seahorses (genus *Hippocampus*): a phylogenetic study using the cytochrome b gene of mitochondrial DNA. Mol Phylogenet Evol 2004; 30: 261–272.

20. Wang X, Han X, Zhang Y, Liu S, Lin Q. Phylogenetic analysis and genetic structure of the seahorse, *Hippocampus fuscus* from the Arabian and Red Sea based on mitochondrial DNA sequences. Mitochondrial DNA A 2019; 30: 165–171.
21. Lourie SA, Green DM, Vincent ACJ. Dispersal, habitat differences and comparative phylogeography of South East Asian seahorses (Syngnathidae: *Hippocampus*). Mol Ecol 2005; 14: 1073–1094.
22. Teske PR, Hamilton H, Palsboll PJ, Choo CK, Gabr H, Lourie SA. et al. Molecular evidence for long-distance colonization in an Indo-Pacific seahorse lineage. Mar Ecol Prog Ser 2005; 286: 249–260.
23. Boehm JT, Waldman J, Robinson JD, Hickerson MJ. Population genomics reveals seahorses (*Hippocampus erectus*) of the western mid-Atlantic coast to be residents rather than vagrants. PloS One 2015; 10: e0116219. http://dx.doi.org/10.1371/journal.pone.0116219.
24. Saarman NP, Louie KD, Hamilton H. Genetic differentiation across eastern Pacific oceanographic barriers in the threatened seahorse *Hippocampus ingens*. Conserv Genet 2010; 11: 1989–2000.
25. Lourie SA, Pritchard JC, Casey SP, Ky TS, Hall HJ. et al. The taxonomy of Vietnam’s exploited seahorses (family Syngnathidae). Biol J Linn Soc 1999; 66: 231–256.
26. Woodall LC, Otero-Ferrer F, Correia M, Curtis JMR, Garrick Maidment N. et al. A synthesis of European seahorse taxonomy, population structure, and habitat use as a basis for assessment, monitoring and conservation. Mar Biol 2018; 165: 1–19.
27. Curtis JMR, Vincent ACJ. Life history of an unusual marine fish: survival, growth and movement patterns of *Hippocampus guttulatus* Cuvier 1829. J Fish Biol 2006; 68: 707–733.

28. Caldwell I, Vincent ACJ. A sedentary fish on the move: effects of displacement on long-snouted seahorse (*Hippocampus guttulatus* Cuvier) movement and habitat use. Environ Biol Fish 2013; 96: 67–75.

29. Correia M, Caldwell IR, Koldewey HJ, Andrade JP, Palma J. Seahorse (Hippocampinae) population fluctuations in the Ria Formosa Lagoon, south Portugal. J Fish Biol 2015; 87: 679– 690.

30. Luzzatto DC, Estalles ML, Diaz De Astarloa JM. Rafting seahorses: the presence of juvenile *Hippocampus patagonicus* in floating debris. J Fish Biol 2013; 83: 677–681.

31. Han SY, Kim JK, Tashiro F, Kai Y, Yoo JT. Relative importance of ocean currents and fronts in population structures of marine fish: a lesson from the cryptic lineages of the *Hippocampus mohnikei* complex. Mar Biodiv 2019; 49: 263–275.

32. Luzzatto DC, Pujol MG, Figueroa D, Diaz De Astarloa JM. The presence of the seahorse *Hippocampus patagonicus* in the deep waters: additional evidence of the dispersive capacity of the species. Mar Biodivers Rec 2014; 7: e71.

33. Harasti D. Range extension and first occurrence of the thorny seahorse *Hippocampus histrix* in New South Wales, Australia. Mar. Biodivers Rec 2015; 8: https://doi.org/10.1017/S1755267215000263.

34. Harasti D. Southward range extension of the great seahorse (*Hippocampus kelloggi* Jordan and Snyder, 1901) in Australia. J Appl Ichthyol 2017; 1–3 https://doi.org/10.1111/jai.13414.

35. Mahapatro D, Mishra RK, Panda S. Range extension of a vulnerable Sea horse *Hippocampus fuscus* (Actinopterygii: Syngnathidae) on the north-eastern Bay of Bengal coast. Mar Biodivers Rec 2017; 10:6. DOI 10.1186/s41200-017-0108-z.

36. Wong NLWS, The JC. Direct observation of drifting juvenile seahorse *Hippocampus trimaculatus* (Leach, 1814) beneath the offshore surface at Malacca Strait. Check List 2017; 13: 993–996.

37. Aylesworth L, Lawson LM, Laksanawimol P, Ferber P, Loh TL. New records of the Japanese seahorse *Hippocampus mohnikei* in Southeast Asia lead to updates in range, habitat and threats. J Fish Biol 2016; 88: 1620–1630.

38. Masuda H, Amaoka K, Araga C, Uyeno T, Yoshino T. The Fishes of the Japanese Archipelago. Tokyo: Tokai University Press; 1984.

39. Kanou K, Kohno H. Early life history of a seahorse, *Hippocampus mohnikei,* in Tokyo Bay Japan. Ichthyol Res 2001; 48: 361–368.

40. Suda Y, Inoue T, Nakamura M, Masuda N, Doi H, Murai T. Nearshore ichthyofauna in the intermediate sandy beach, Doigahama Beach, Yamaguchi Prefecture, Japan. J Nat Fish Univ 2004; 52: 11–29.

41. Lim K. New Singapore record of the seahorse, *Hippocampus mohnikei*. Singapore Biodiver Rec 2013; 2013: 68–69.

42. Laksanawimol P, Petpiroon S, Damrongphol P. Trade of seahorses, *Hippocampus* spp. (Actinopertygii: Syngnathiformes: Syngnathidae), on the East Coast of the Gulf of Thailand. Acta Ichthyol Piscata 2013; 3: 229–235.

43. Chang CH, Jang-Liaw NH, Lin YS, Fang YC, Shao KT. Authenticating the use of dried seahorses in the traditional Chinese medicine market in Taiwan using molecular forensics. J Food Drug Analy 2013; 21: 310–316.

44. Zhang Y, Pham NK, Zhang H, Lin J, Lin Q. Genetic variations in two seahorse species (*Hippocampus mohnikei* and *Hippocampus trimaculatus)*: evidence for Middle Pleistocene population expansion. PloS One 2014; 9: e105494.

45. Pollom R. *Hippocampus mohnikei*. The IUCN Red List of Threatened Species 2017: http://dx.doi.org/10.2305/IUCN.UK.20173.RLTS.T41005A54907304.en43. Downloaded on 10 November 2018.

46. Song Z, Song J, Yue B. Population genetic diversity of Prenant's schizothoracin, *Schizothorax prenanti*, inferred from the mitochondrial DNA control region. Environ Biol Fish 2008, 81: 247–252.

47. Shetye SR, *Gouveia AD*, *Singbal* SY, Naik CG, Sundar D. et al. Propagation of tides in the Mandovi–Zuari estuarine network. Proc Indian Acad Sci (Earth Planet Sci) 1995; 104: 667–682.

48. Qasim SZ, Sen Gupta R. Environmental characteristics of the Mandovi-Zuari estuarine system. Estuar Coast Shelf Sci *1*981; 13: 557–578.

49. Madhupratap M, Nair KNV, Gopalkrishnan TC, Haridas P, Nair KKC. et al. Arabian sea oceanography and fisheries of the west coast of India. *Curr Sci 2001; 81: 3*55–361.

50. Ansari ZA, Sreepada RA, Dalal SG, Ingole BS, Chatterji A. Environmental influences on the trawl catches in a bay-estuarine system of Goa, west coast of India. Estuar Coast Shelf Sci 2003; 56: 503–515.

51. Ansari ZA, Chatterji A, Ingole BS, Sreepada RA, Rivonker CU. et al. Structure of inshore demersal fish community at Goa. Estuar Coast Shelf Sci 1995; 41: 593–610.

52. Lourie S. Measuring seahorses. Project Seahorse Technical Report No.4, Version 1.0. Project Seahorse, Fisheries Centre, University of British Columbia; 2003.

53. Zhang Y, Qin G, Wang X, Qiang L. A new species of seahorse (Teleostei: Syngnathidae) from the South China Sea. Zootaxa 2016; 4170: 384–392.

54. Wibowo K, Tashiro S, Motomura H. Seahorses of the genus *Hippocampus* (Syngnathidae) from Kagoshima Prefecture, southern Japan, with a new record of *H. mohnikei* Bleeker, 1853. Nature of Kagoshima 2017; 43:71–76.

55. Underwood AJ. Experiments in Ecology: their logical and interpretation using Analysis of Variance. Cambridge University Press. Cambridge, UK; 1997.

56. Kumar R Singh PJ, Nagpure NS, Kushwaha B, Srivastava SK. et al. A non-invasive technique for rapid extraction of DNA from fish scales. Ind J Exp Biol 2007; 45: 992–997.

57. Qin G, Zhang Y, Ho ALFC, Zhang Y, Lin Q. Seasonal distribution and reproductive strategy of seahorse. ICES J Mar Sci 2017; 74: 2170–2179.

58. Lourie SA, Vincent ACJ. A marine fish follows Wallace's line: the phylogeography of the three-spot seahorse (*Hippocampus trimaculatus*, Syngnathidae, Teleostei) in Southeast Asia. J Biogeogr 2004; 31: 1975–1985.

59. Altschul SF, Gish W, Miller W, Myers EW, Lipman DJ. Basic local alignment search tool. J Mol Biol 1990; 215: 403–410.

60. Kumar S, Stecher G, Tamura K. MEGA7: Molecular Evolutionary Genetics Analysis, version 7.0 for bigger datasets. Mol Biol Evol 2016; 33: 1870–1874.

61. Kimura M. A simple method for estimating evolutionary rates of base substitutions through comparative studies of nucleotide sequences. J Mol Evol 1980; 16: 111–120.

62. Choi Y, Rho S, Park H, Kang D. Population characteristics of two seahorses, *Hippocampus coronatus* and *Hippocampus mohnikei*, around seagrass beds in the southern coastal waters of Korea. Ichthyol Res 2012; 59: 235–241.

63. Kwak S, Huh S, Kim H. Feeding habits of *Hippocampus mohnikei* in an eelgrass (*Zostera marina*) bed. Korean J Ichthyol 2008; 20: 112–116.

64. Panithanarak T, Karuwanjaroen R. A preliminary study of genetic differentiation in the Japanese seahorse (*Hippocampus mohnikei*) populations: a possibility of cryptic species. Burapha Sci J 2019; 24: 124–137.
65. Hou F., Wen L, Peng C, Guo J. Identification of marine traditional Chinese medicine dried seahorses in the traditional Chinese medicine market using DNA barcoding. Mitochondrial DNA A 2018; 29(1):107–112.
66. Otsuka Y, Suzuki H, Akagawa I. Occurrence, gonad morphology and maturity of Japanese seahorse *Hippocampus mohnikei* in Matsushima Bay, Japan. J School Mar Sci Tech Tokai Univ 2009; 7: 11–22.
67. Wafar S. Ecology of mangroves along the estuaries of Goa; Ph. D. Thesis, Karnataka University, Dharward, India; 1987.
68. Jagtap TG. Distribution of seagrasses along Indian coast. Aquac Bot 1991; 40: 379–386.
69. Padmavati G, Goswami SC. Zooplankton ecology in the Mandovi-Zuari estuarine system of Goa, west coast of India. Ind J Mar Sci 1996; 25: 268–273.
70. Dalal SG, Goswami SC. Temporal and ephemeral variations in copepod community in the estuaries of Mandovi and Zuari– west coast of India. J Plankton Res 2001; 23: 19–26.
71. Das PB. Zooplankton assemblages in spatial stretches of a tropical estuary. Int J Res Appl Sci Engg Technol 2017; 5: 1789–1799.
72. Qin G, Johnson C, Zhang Y, Zhang H, Yin J, Miller G. et al. Temperature-induced physiological stress and reproductive characteristics of the migratory seahorse *Hippocampus erectus* during a thermal stress simulation. Biol Open 2018; 25: 7(6).bio032888.

73. Scales H. Advances in the ecology, biogeography and conservation of seahorses (genus *Hippocampus*). Prog Phys Geog 2010; 34: 443–458.

74. Bohonak AJ. Dispersal, gene flow, and population structure. Q Rev Biol 1999; 74: 21–45.

75. Palumbi SR. Genetic divergence, reproductive isolation, and marine speciation. Annu. Rev Ecol Evol S 1994; 25: 547–572.

76. Wyrtki K. Physical oceanography of the southeast Asian waters. Scientific results of maritime investigations of the South China Sea and the Gulf of Thailand 1959–1961. Naga Report, 2. Scripps Institution of Oceanography, La Jolla, CA; 1961.

77. Shetye SR, Gouveia AD, Shenoi SSC, Michael GS, Sundar D, Almedia AM. et al. The coastal current off western India during the northeast monsoon. Deep-Sea Res 1991; 38: 1517–1529.

78. Fleminger A. The Pleistocene equatorial barrier between the Indian and Pacific oceans and a likely cause for Wallace's Line. In Pierrot-Bults AC, van der Spoel S, Zahuranec BJ, Johnson RK editors, Pelagic marine science. UNESCO Tech Pap Mar Sci 1986; 49: 84–97.

79. Maree RC, Whitfield AK, Booth AJ. Effect of water temperature on the biogeography of South African estuarine fishes associated with the subtropical/warm temperate subtraction. S Afr J Sci 2000; 96: 184–188.

80. Bowen BW, Bass AL, Rocha LA, Grant WS, Robertson DR. Phylogeography of the trumpetfishes *(Aulostomus)*: ring species complex on a global scale. Evolution 2001; 55: 1029–1039.

81. Martin-Smith KM, Vincent ACJ. Seahorse declines in the Derwent estuary, Tasmania, in the absence of fishing pressure. Biol Conserv 2005; 123: 533–545.

82. Shokri MR, Gladstone W, Jelbart J. The effectiveness of seahorses and pipefish (Pisces: Syngnathidae) as a flagship group to evaluate the conservation value of estuarine seagrass beds. Aquat Conserv 2009; 19: 588–595.

Supporting Information

S1 Fig. Picture of *H. mohnikei* posted on social media. Male specimen of *Hippocampus mohnikei* caught by gill net in the Chapora estuary, Goa, India

S1 Table. The COI and Cyt *b* gene sequences. GenBank accession numbers and sources of the mitochondrial gene sequences (COI and Cyt *b*) of seahorse species used for construction of phylogenetic trees.

S2 Table. The COI and Cyt *b* gene sequences. GenBank accession numbers and sources of the mitochondrial gene sequences (COI and Cyt *b*) of seahorse species used for construction of phylogenetic trees.

Figure

Click here to access/download;Figure;Fig 1.tif

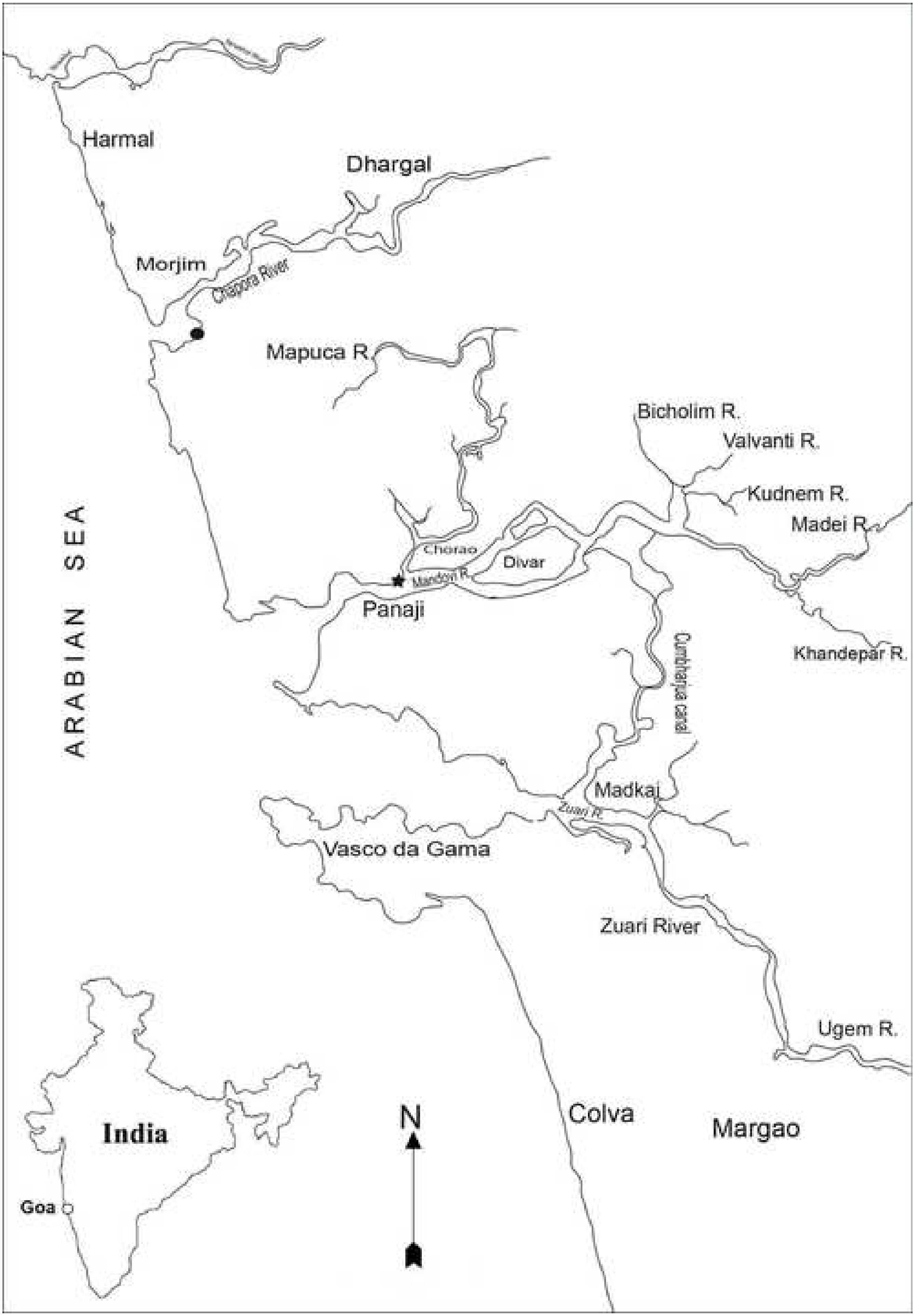

Figure

Click here to access/download;Figure;Fig 2.tif

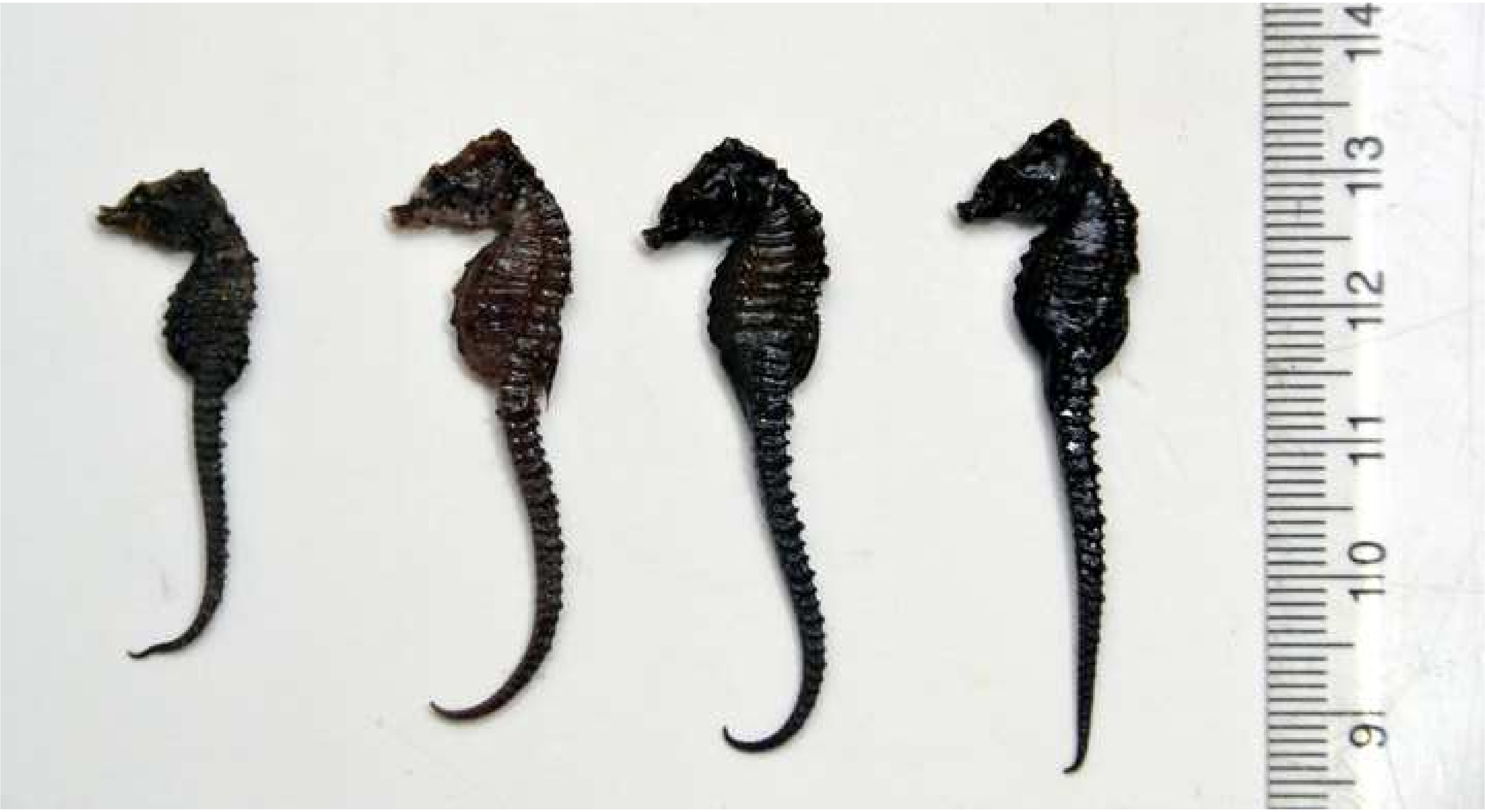

Figure

Click here to access/download;Figure;Fig 3.tif

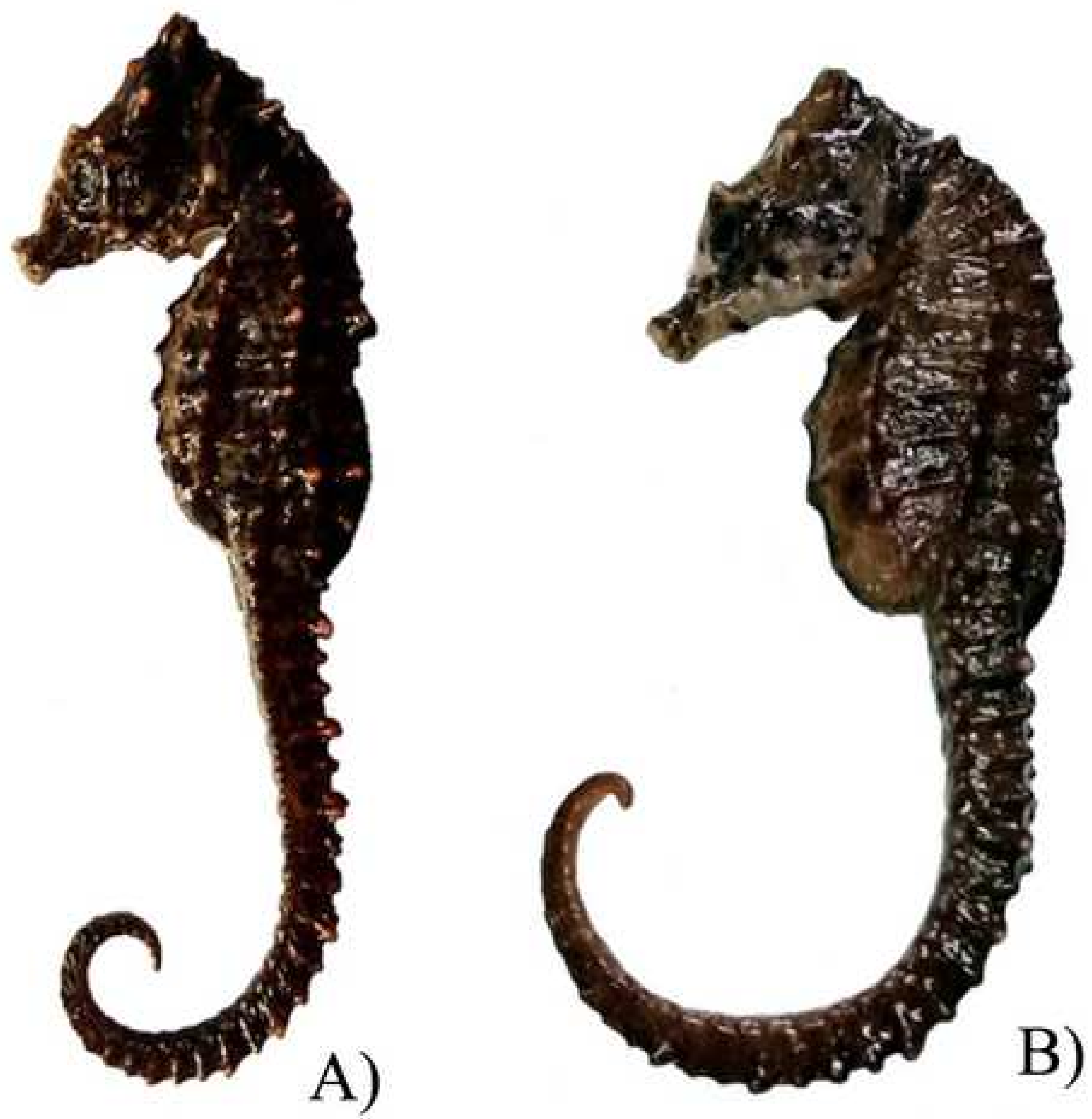

Figure

Click here to access/download;Figure;Fig 4.tif

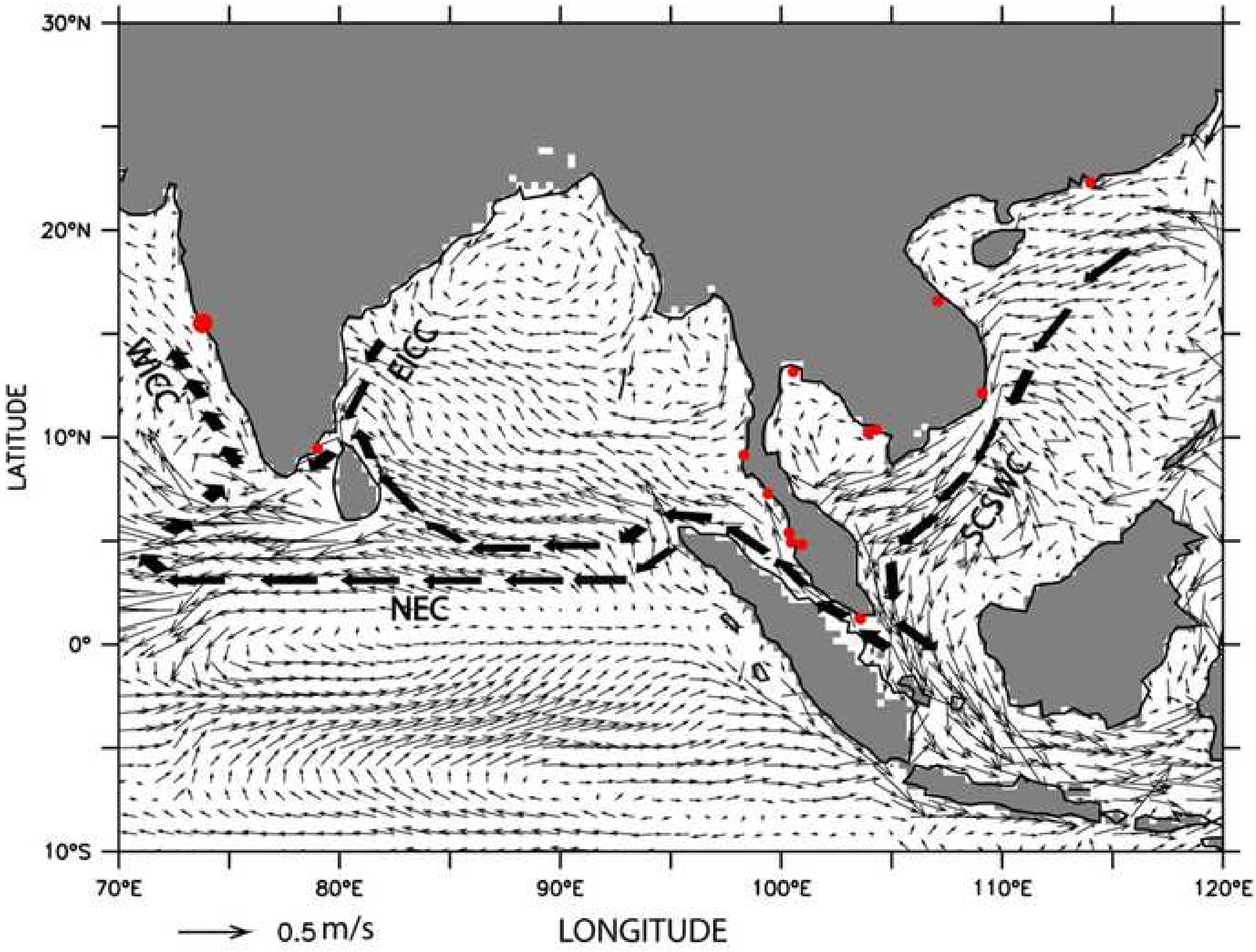

Figure

Click here to access/download;Figure;Fig. 5.tif

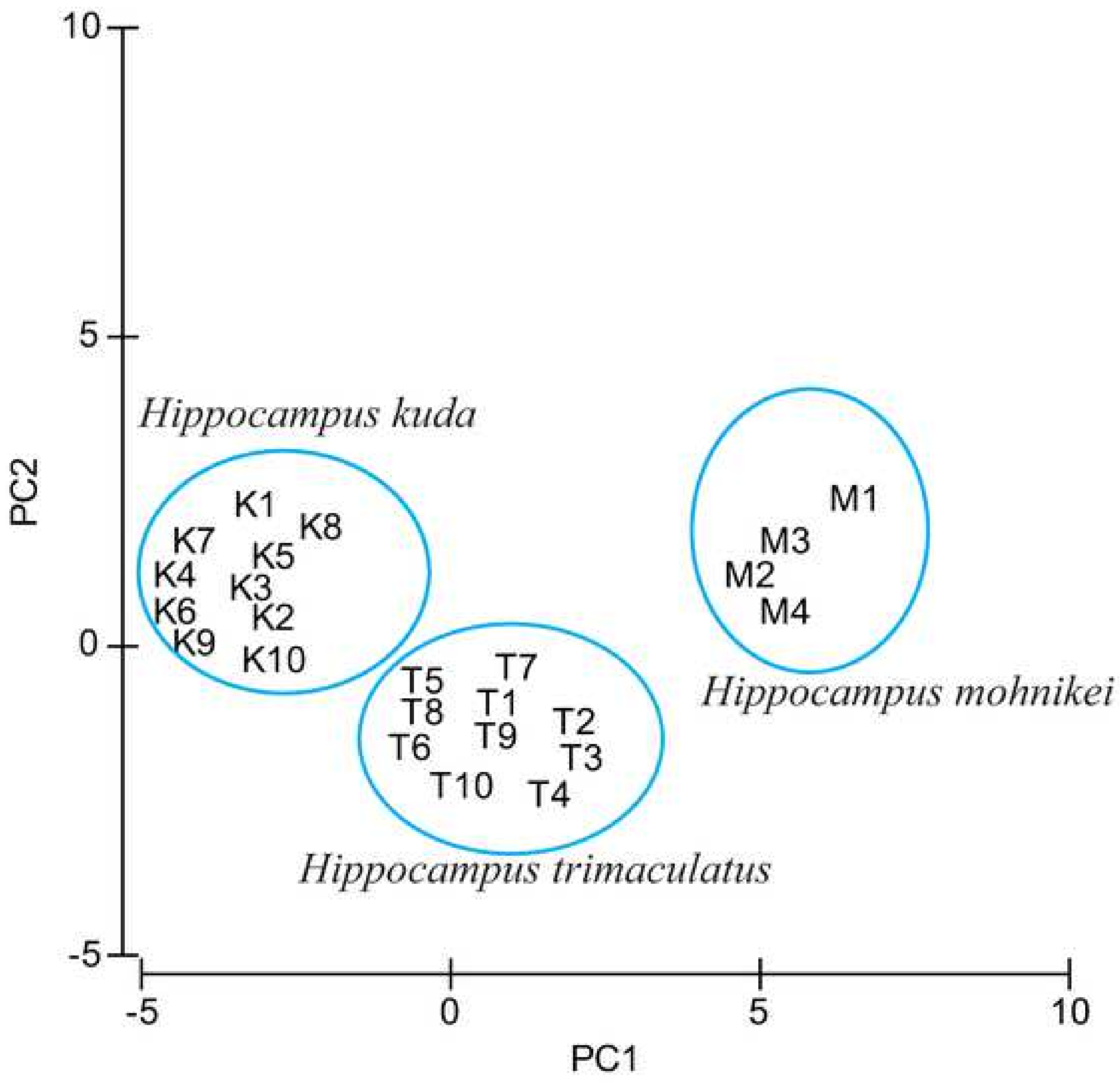

Figure

Click here to access/download;Figure;Fig 6.tif

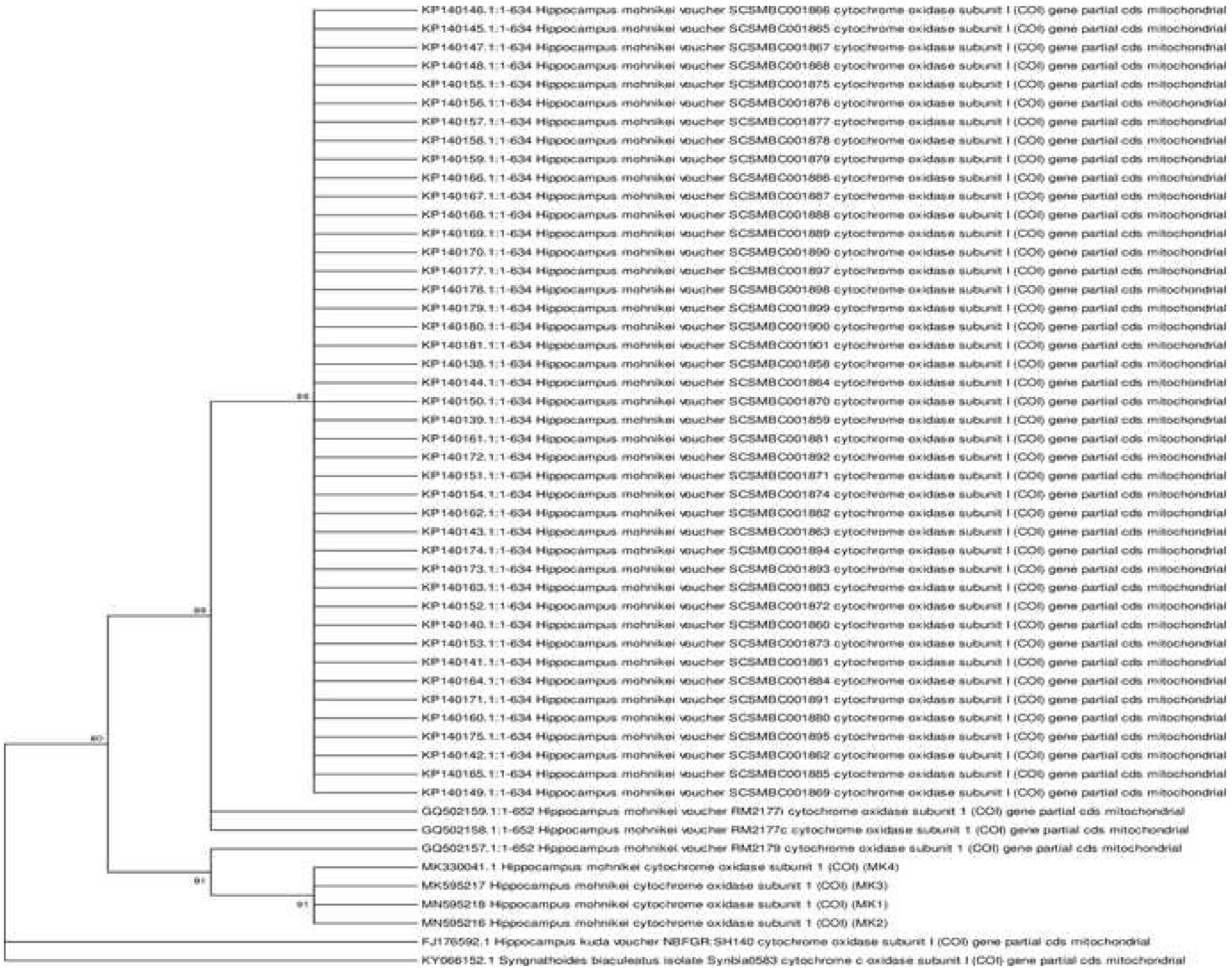

Figure

Click here to access/download;Figure;Fig 7.tif

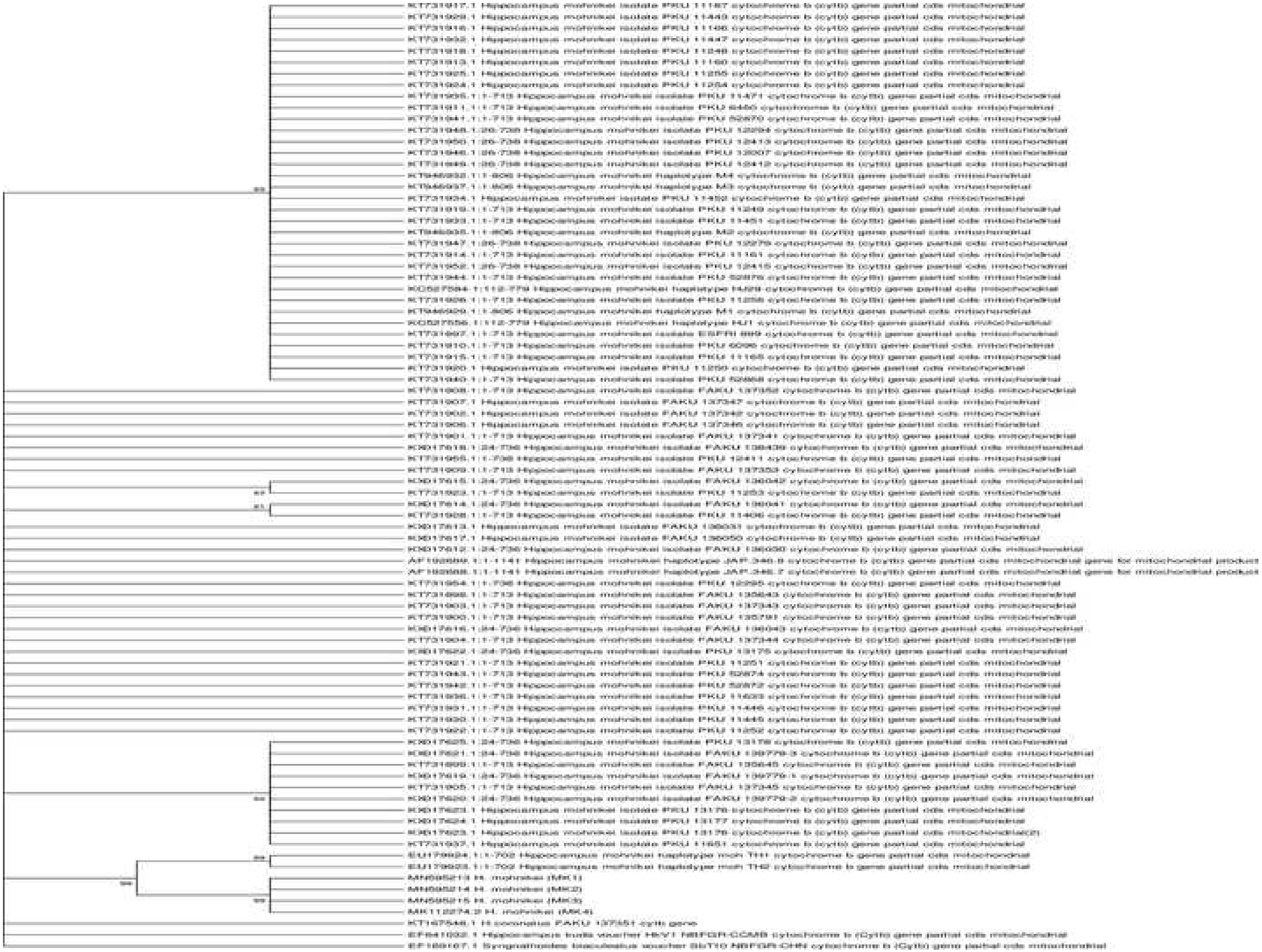

Figure

Click here to access/download;Figure;Fig 8.tif

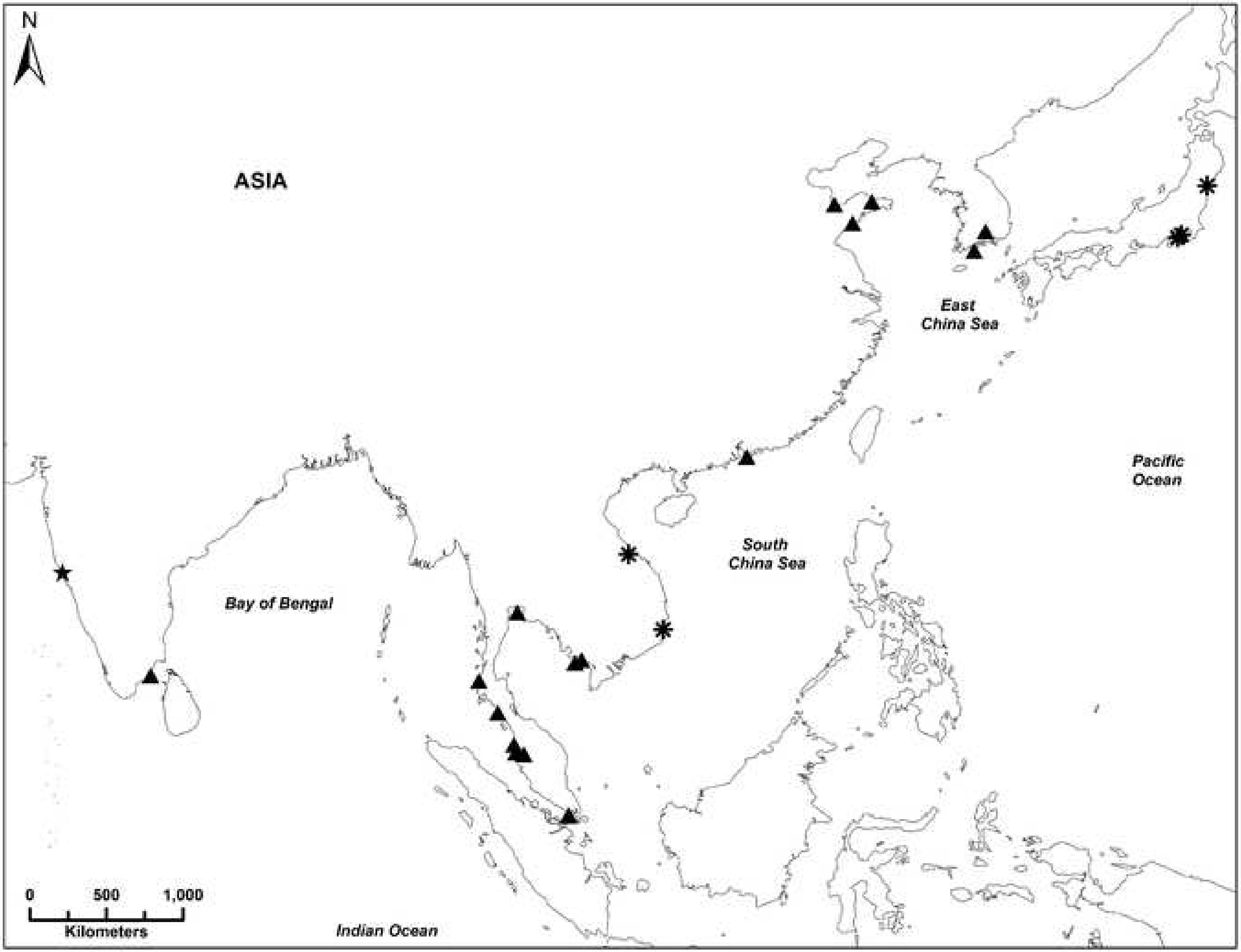

Click here to access/download
Supporting Information
S1 Fig.tif

Click here to access/download

Supporting Information

S1 Table.doc

Click here to access/download
Supporting Information
S2 Table.docx

Morphological and molecular evidence for range extension and first occurrence of the Japanese seahorse, *Hippocampus mohnikei* (Bleeker 1853) in a bay-estuarine system of Goa, central west coast of India

Sushant Sanaye[1], Rakhee Khandeparker[1], Rayadurga Anantha Sreepada[1*], Mamatha Singanahalli Shivaramu[1], Harshada Kankonkar[1], Jayu Narvekar[2], Mukund Gauthankar[1]

[1]Aquaculture Laboratory, Biological Oceanography Division, CSIR-National Institute of Oceanography, Dona Paula, Goa–403 004, India

[2]Physical Oceanography Division, CSIR-National Institute of Oceanography, Dona Paula, Goa–403 004, India

*sreepada@nio.org; sreepada63@gmail.com

Formatted: Superscript

Abstract

Accurate information of taxonomy and geographic ~~distribution~~ range of seahorse species (genus *Hippocampus*) is the first step in preparing threat assessments and designing effective conservation measures. Here, we report the range expansion and first occurrence of the Japanese seahorse, *Hippocampus mohnikei* (Bleeker, 1853) from the Mandovi estuarine ecosystem of Goa, central west coast of India (CWCI) based on morpho-molecular analyses. The morphometric and meristic traits particularly, short snout~~ length~~ (29–35% head length), double cheek spine, low coronet and long tail (51.2–57.9% of standard length), 11 trunk rings, 37–39 tail rings, 15–16 dorsal and 12–14 fin rays observed in ~~all~~ four collected specimens matched with the reported key diagnostic morphological criteria of *H. mohnikei*. The seahorse mitochondrial cytochrome oxidase subunit I and cytochrome *b* genes were partially sequenced for conclusive genetic identification of the species under study. Using the maximum similarity with GenBank database, maximum likelihood network and subsequent morphological analysis, the identity of the collected seahorse species was reconfirmed as *H. mohnikei*. With this new report, the ~~distributional~~geographic range of *H. mohnikei* extended significantly to the westward side from its hitherto known range of Japan and Vietnam. This new sighting of *H. mohnikei* could indicate a long-distance dispersal ~~T~~facilitated by ~~he possible role o~~due to ~~f~~ prevailing oceanic circulation in the Indo-Pacific region or increased habitat suitability in bay-estuarine systems of Goa, CWCI. ~~in facilitating dispersal and range extension of *H. mohnikei* has been highlighted~~. Comparison of the pair-wise genetic distances (Kimura 2-parameter) based on COI and Cyt *b* sequences revealed that the specimens examined in this study are genetically closer to *H. mohnikei* populations from Vietnam and Thailand ~~revealed that the conspecificity of Goa specimens with populations of *H. mohnikei* from Vietnam and Thailand rather~~ than they are to those in Japan. To test the hypothesis

Formatted: Font: Italic

Formatted: Font: Italic

whether *H. mohnikei* populations are vagrants or residents of a bay-estuarine system, long-term inter-annual sampling and analyses are warranted.

Introduction

Seahorses (Syngnathiformes; Syngnathidae) belonging to the single genus, *Hippocampus* (Rafinesque, 1810) are a fascinating and remarkable group of fishes with their unusual body shape and their biology, with males incubating the fertilized eggs in a brood pouch [1]. They are small, cryptic, and sedentary marine fishes that occur worldwide in shallow temperate to tropical waters in a wide variety of habitats, including seagrass beds, estuaries, coral and rocky reefs and mangroves [2−4]. Their remarkable ability to camouflage with structurally complex habitats further reduces the risk of predation [5], while providing stealth for prey capture [6]. However, such biological traits often also make them unnoticeable from coastal habitat, particularly in those regions where seahorse trade is absent. ~~Often overlooked by most fishermen, ~~Due to their cryptic nature, their ability to camouflage and the sparse distribution, seahorses become almost unnoticeable to fishermen and thus restricting~~ restric~~ the ~~t~~ biologists for their ~~to ~~identification~~ y their presence~~ in dense ~~thick~~ coastal marine habitats.

Out of 4~~1~~4 recognised ~~valid~~ seahorse species (Genus *Hippocampus*) throughout the world [7], *Hippocampus kelloggi*, *H. kuda*, *H. spinosissimus* and *H. trimaculatus* are most common in Indian coastal marine waters [8,9]. In addition, sightings based on morphological identification of single specimens, *H. mohnikei* from the Palk Bay, southeastern India [10], *H. borboniensis* [11] and *H. montebelloensis* [12] from Gulf of Mannar, southeast coast of India and *H. camelopardalis* from Gulf of Kachchh, northwest coast of India [13] have been reported. Another seahorse species, *H. histrix* is also suspected to occur in Indian coastal waters [14]. However, the validity of *H. bornoinensis* and *H. montebelloensis* as separate

Formatted: Indent: First line: 0.3"

Formatted: Font: Italic

species is under revision due to their synonimity with *H. kuda* and *H. zebra*, respectively [15].

The utility of morphological traits commonly used in diagnosis of *Hippocampus* spp. be is quite challenging as they lack certain key physical features (e.g. pelvic and caudal fins), and have high variability of ~~and~~ overlapping ~~of~~ body proportions, colour (camouflage) and skin filaments, fin-ray number and trunk-ring number may vary greatly within any given-species and between species [3,~~15]~~[16]. On the other hand, molecular methods have proved helpful in resolving morphologically challenging seahorse taxonomy [~~7~~15,~~16~~17], phylogenetic relationships within genus, *Hippocampus* [18~~17~~,1~~8~~9], natural species boundaries [20~~19~~–2~~1~~2] and genetic variability [2~~0~~1,2~~2~~3] of many seahorse species. An integrated approach of combination of morphological and genetic analyses [2~~3~~4] would aid in the management of demographically separate populations as independent units and allow international legal mechanisms and international agreements such as the convention on international trade in endangered species (CITES) to work effectively [2~~4~~5].

Generally, seahorses are sedentary fish species with small home range and patchy distribution in sheltered areas such as seagrasses and seaweeds [2,2~~5~~6,2~~6~~7]. However, instances of limited ~~small~~ migrations ~~of~~ by seahorses in search of proper habitat, food or holdfasts have been reported [2~~6~~7,2~~7~~8]. Evidence of long-distance dispersal of seahorse species via rafting (floating debris and seaweeds) [2,2~~8~~9,9] and subsequent colonisation [20] have been documented. The role of oceanic currents facilitating ~~as stepping stones~~ in long-distance dispersal and range expansion of many seahorse species has also been highlighted [30~~2~~9–3~~4~~5].

The Japanese seahorse or Lemur-tail seahorse, *Hippocampus mohnikei* (Bleeker, 1853) is a small coastal seahorse species (5–8 cm) inhabiting seagrasses, mangroves, oyster beds and mussel farms [36]. ~~along the coasts of continental Asia from southeastern India to Korea and~~

Formatted: Font: Italic

Formatted: Font: Italic

Formatted: Font: Not Italic

Formatted: Font: Not Italic

Japan [35]. Original records Previous reports [156,367−389] indicated that the distribution of *H. mohnikei* is restricted or native to the coastal waters around Japan and Vietnam [16,37−39]. However, new records of *H. mohnikei* from Cambodia, Malaysia, Thailand and Vietnam, along with recently published studies from southeastern India and Singapore, have greatly expanded the known geographical range of *H. mohnikei* within southeast Asia [36]. As a consequence of suspected reduction in population size of >30% over the past 10 years due to fisheries exploitationrecent exploitations [356,4039−412], the status of *H. mohnikei* in the IUCN Red List of Threatened Species has been listed as 'vulnerable'VULNERABLE' [423].

A probable occurrence of *H. mohnikei* along the central west coast of India (CWCI) began in February 2017 when a fisherman posted a picture of seahorse incidentally caught in gill net operated in the Chapora estuary, Goa (India) (15.6120° N, 73.7506° E) on social media [S1 Fig.] which was suspected to be an adult male of *H. mohnikei*. Sustained follow-up surveys for its occurrence in the surrounding environs of the Chapora estuary did not yield in any further reporting. However, four sub-adult specimens of *H. mohnikei*, hitherto sighted only from southeast coast of India [10], caught as an incidental catch in bag net fisheries during July, 2018 in the Mandovi estuarine system, Goa CWCI were examined. This contribution describes the range expansion and first occurrence of *H. mohnikei* from the western coastal India. Mandovi estuarine ecosystem, Goa, CWCI. To test further the hypothesis that the morphologically identified seahorse species is *H. mohnikei*, two mitochondrial DNA gene regions (loci), cytochrome oxidase subunit I (COI) and cytochrome *b* (Cyt *b*) genes) were sequenced for reconfirmation. Potential role of ocean currents on the range expansion of *H. mohnikei* to north-western Asia have also been discussed. In the wake of the vulnerability of seahorse populations to the threats such as habitat

Formatted: Indent: First line: 0.3", Add space between paragraphs of the same style

Formatted: Font: Not Italic, Not Highlight

Formatted: Font: Italic

Formatted: Font: Not Italic, Not Highlight

alteration/destruction and fishing pressure globally, the present sighting of *H. mohnikei* in a bay-estuarine system is of considerable conservation and biogeographic significance.

Materials and Methods

Collection site and seahorses

The locality (Brittona, Goa, India, 15.3059°N, 73.5073° E, Fig 1) at which the examined seahorse specimens were collected ~~collection site of seahorses (Brittona, Goa, India, 15.3059°N, 73.5073° E, Fig 1)~~ is a shallow water littoral environment ~~which is~~ in the proximity to the mangrove-dominated Chorao Island in the Mandovi estuary, CWCI. The currents are largely tide dominated, the tides being semi-diurnal with a mean amplitude of 1.3 m. Currents are also influenced by the large seasonal freshwater influx [4~~3~~4] during the summer monsoon season (June to September). Temporal variations in abiotic and biotic factors influenced by monsoonal precipitation and seasonal upwelling render the collection location as one of the most ecologically complex ecosystems [4~~4~~5,4~~5~~6]. Significant lowering of salinity occurs when the freshwater discharge is at its maximum during monsoon. During the post- and pre-monsoon periods, the salinity at the surface and bottom remains high (~25 ppt). The bottom topography at the collection site is ~~characterized by~~ muddy and comprises mainly of silt and clay with rich organic matter content [4~~6~~7]. The collection site is reported to harbour rich and diverse fishery resources and its importance in supporting the life history stages of several marine teleosts has also been well-documented [4~~7~~8].

Present report is based on four specimens of *H. mohnikei* (females, MK1 and MK2; males, ~~–~~MK3 and MK4; Fig. 2. and Fig. 3) that were incidentally caught in bag nets attached to the fixed stakes (total length, 25 m; mouth length, 5 m; cod end mesh size, 10 mm) operated at the water depth of ~6 m in the Mandovi estuary during July 2018. ~~Freshly landed~~ ~~d~~Dead seahorses landed as incidental catch in bag nets attached to the stakes operated by

Formatted: Indent: First line: 0.3"

local fishermen in the estuarine system formed the study material. No live seahorses were specifically caught for the present study. Fin clips from these dead seahorses were used for molecular analysis. The present study did not involve any experimentation with live animals and hence ethics committee's approval for the usage of live animals for experiments is inconsequential.

Fig 1. Sampling localities of *H. mohnikei*. A map showing major rivers and estuarine systems ~~es~~ of Goa (central west coast of India). Location of a suspected adult male *H. mohnikei* in Chapora estuary (●); collection site of confirmed report of *H. mohnikei* in the Mandovi estuary (★).

Fig 2. Collected seahorses from Goa. Sub-adult specimens of *Hippocampus mohnikei* (Bleeker 1853) (females, MK1 and MK2; males, MK3 and MK4; NIO1015/19) from the Mandovi estuary, Goa, central west coast of India.

~~Specimens of *Hippocampus mohnikei* (MK1 and MK2 = females; MK3 and MK4 = males) collected from the Mandovi estuary, Goa, central west coast of India.~~

Fig 3. Close-up view of *H. mohnikei*. *Hippocampus mohnikei* (Bleeker 1853) (A-male; B-female) from the Mandovi estuary, Goa, central west coast of India (NIO1015/19).

~~A closer view of *Hippocampus mohnikei* (A-male; B-Female) collected from the Mandovi estuary, Goa, central west coast of India.~~

Morphological analysis

Before subjecting them to a detailed analysis of morphological characteristics, seahorses were carefully photographed (Nikon D7200, Japan) and sexed via observation of presence or

absence of brood pouch. Morphometric measurements and meristic counts were carried out following ~~as per~~ standardized protocols [14,1~~5~~6,4~~8~~9]. Morphometric characters such as height (Ht, linear distance from the top of the head to the tip of the stretched tail), ~~standard length (SL),~~ head length (HL), trunk length (TrL), ~~and~~ tail length (TaL) and standard length (SL = HL+TrL+TaL), were measured. Measurements of snout length (SnL), ratio of head/snout lengths (HL/SnL), snout depth (SnD), head depth (HD), pectoral fin base length (PL) and dorsal fin base length (DL) were also conducted. All measurements were taken with the help of digital caliper to 0.1 mm and repeated to ensure accuracy. ~~t~~Trunk depths on 4th ~~trunk ring~~ (TD4) and 9th ~~trunk ring~~ (TD9) trunk rings were measured. The presence/absence of spines and dermal appendages was also noted. Meristic characters such as the number of trunk rings (TrR), tail rings (TaR), dorsal fin rays (DF), pectoral fin rays (PF) and anal fin rays (AF) were counted under stereo zoom microscope (Olympus SZX7, Japan) and the values were confirmed by ~~three time~~ triplicate counting. Brood pouch dimensions were measured in all male ~~also taken in case of male~~ specimens.

Morphological identification of the collected seahorse specimens from Goa was based on comparison of the ~~The~~ salient morphometric and meristic characters ~~of the collected seahorse specimens were compared~~ with the reported diagnostic features for different seahorse species [14,1~~5~~6,2~~3~~4]. The voucher specimen~~material~~ is deposited in the national marine biodiversity repository at CSIR-National Institute of Oceanography, Goa, India (deposition ID: NIO1015/19).

Statistical analysis

Morphological data of *H. mohnikei* and two other commonly occurring seahorse species (*H. kuda* and *H. trimaculatus*) in the study area was evaluated by one-way analysis of variance (ANOVA) followed by the Duncan's multiple-range tests [50~~49~~]. The level of significance

Formatted: Indent: First line: 0.3"

was tested at 5.0%, represented as $P < 0.05$. A comparison of morphological characters and meristic features of *H. mohnikei* (n = 4) with *H. kuda* (n = 10) and *H. trimaculatus* (n = 10) was made through principal component analysis (PCA). PCA was employed in which factor loadings based on components were used to determine the morphometric factors using PRIMER software (version 6) after square-root transformation.

Molecular analysis: DNA extraction, PCR amplification and sequencing

For gene-based phylogenetic analysis, fragments of dorsal fin from freshly landed dead seahorses were removed and preserved in 95% ethanol at –20°C until subjected to DNA extraction. DNA extraction was carried out as described [5~~0~~1] with a few modifications. In brief, lysis buffer and sodium dodecyl sulfate were added to the fin tissue and vortexed. Further, RNase and Proteinase K were added to the mixture and incubated for 15–20 minutes at room temperature. After the incubation period, the DNA was extracted using phenol:chloroform:isoamyl alcohol (25:24:1) mixture. Precipitated DNA was washed with 70% cold ethanol. The gel electrophoresis was performed to confirm the quality and integrity of DNA.

Seahorse mitochondrial cytochrome oxidase subunit I (COI) gene was amplified using primers, forward (5'TCAACTAATCACAAAGACATCGGCAC3′) and reverse (5'ACTTCGGGGTGCCCAAAGAATC3′) [5~~1~~2]. Similarly, mitochondrial cytochrome *b* (Cyt *b*) gene fragment was also amplified using PCR with seahorse-specific primers, forward (5'AACYAGGACYAATGRCTTGA3’) and reverse (5′GCASWAGGGAGGRKTTTAAC3′) [40]. Reactions were carried out in a thermal cycler (Veriti® 96-well Thermal Cycler, Applied Biosystems, CA, USA). The amplification of COI and Cyt *b* genes was carried out by following cycling conditions as described [5~~2~~3] with few modifications; an initial denaturation step of 95°C for 2 minutes followed by 39 cycles of 94°C for 30s, 55°C for 30s

72°C for 1 minute and a final extension at 72°C for 2 minutes. Amplified PCR products of both COI and Cyt *b* genes were verified on agarose gel (1.5%) and purified for sequencing using PCR purification kit (Sigma Aldrich, USA). Gene sequence of the PCR products was determined using a taq dye deoxy terminator cycle sequencing kit (PerkinElmer, CA, USA) following supplier's protocols. Sequencing reaction products were analyzed on 373 automated DNA Sequencer (Model 313 xl; Applied Biosystems, CA, USA). Amplification of the mitochondrial COI and Cyt *b* genes resulted in fragments of 609 bp and 639 bp, respectively.

Phylogenetic analysis

Nucleotide Ssequences generated of four Goa specimens collected from Goa (MK1, MK2, MK3 and MK4) are deposited –in (GenBank under accession numbers MN595216, MN595218, MN595217 and MK330041.1 for COI gene and MN595213, MN595214, MN595215 and MK112274.2 for COI and Cyt *b* genes, respectively). Sequence integrity and genetic affinity with known species was compared using the basic local alignment search tool (BLAST) of NCBI [534] (http://www.ncbi.nih.gov/BLAST). Sequences of Goa specimens were aligned with those of morphologically similar seahorse species (*H. coronatus* and *H. mohnikei*) and the most commonly occurring species in the Indo-Pacific region (*H. camelopardalis*, *H. comes*, *H. histrix*, *H. kelloggi*, *H. kuda*, *H. sindonis*, *H. spinosissimus* and *H. trimaculatus*) retrieved from the GenBank database. Accession number of sequences, which were downloaded, is presented given in Table 1. (Table S1. Table). The mitochondrial COI and Cyt *b* gene sequences were aligned using the ClustalW algorithm implemented in MEGA 7 [545]. To ascertain stable and reliable relationships among the seahorse species, phylogenetic trees were constructed through maximum likelihood (ML) method. The analyses were performed in MEGA 7, and confidence levels were assessed using 1000

bootstrap replications. The alligator pipefish, *Syngnathoides biaculeatus* was used as an outgroup species for phylogenetic analysis to differentiate between different seahorse species. The genetic distances of *H. mohnikei* from different geographical locations were calculated using Kimura 2-parameter (K2P) distance model [5~~5~~6] implemented in MEGA 7 [54]. Codon positions included were $1^{st}+2^{nd}+3^{rd}$+noncoding. All ambiguous positions with alignment gaps and missing data were removed from the sequences (pair-wise deletion option).

Prevailing ocean circulation

In order to understand the basin-wide circulation in the Indo-Pacific region (encompassing South China Sea, East China Sea, and southeastern Bay of Bengal), monthly mean climatology was prepared based on daily data on U (zonal) and V (meridional) components of geostrophic current velocities derived from Aviso merged product for the years, 2007–2017. Altimeter products by Ssalto/Duacs and distributed by Aviso+, with support from Cnes (https://www.aviso.altimetry.fr) were used (Fig. 4).

Fig. 4. Oceanic circulation in Indo-Pacific region. Climatological surface currents during winter season (December–February) for the Indo-Pacific region. Red dots indicates the geographic locations of distribution of *Hippocampus mohnikei.* (WICC= West Indian Coastal Current, EICC= East India Coastal Current, NEC= North Equatorial Current and SCSWC= South China Sea Warm Current).

Results

Specimens ~~Material~~ examined

Colour of the head and body of freshly collected seahorse specimens (females, MK1 and MK2; males, MK3 and MK4; NIO1015/19) was ~~almost~~ dark brown to dark black. The entire

body was covered with low spines and the coronet was observed to be short with five tiny projections. Two prominent cheek spines and double rounded two small rounded eye spines below the eye on each side of the head were discernible in all four specimens. (Fig 2. and Fig 3.).

Morphological taxonomy

~~Data on detailed morphometric measurements and meristic counts recorded on four seahorse specimens from Goa are presented in Table 1.~~ ~~Morphological measurements revealed~~

Measured height (Ht) and standard length (SL) of specimens varied between 39.2 and 54.2 mm (48.2 ± 6.5 mm) and 47.3 and 60.1 mm (54.95 ± 5.48 mm), respectively. Trunk lengths (TrL) and tail lengths (TaL) varied between 15.1 and 19.4 mm (16.45 ± 2.02 mm) and 24.2 and 33.1 mm (30.15 ± 4.04 mm), respectively. TaL was observed to be relatively long compared to the TrL of the body. The ratio of the head length (HL) to snout length (SnL) of all four specimens varied between 3.2 and 3.5. Number of trunk rings (TrR) in all specimens were 11 whereas, the number of tail rings (TaR) varied narrowly between 37 and 39. The number of TrR supporting dorsal fin was two and the number of TaR supporting dorsal fin was counted to be one in all specimens. Number of dorsal fin ~~rays,~~ pectoral fin ~~rays~~ and anal fin rays recorded were 15–16, 12–14 and 4, respectively. Slight enlargements, respectively of 1st, 4th, 7th, and 10/11thTrR and 4th, 7/8th, 9/10th, 13/14thTaR were prominently discernible.

Morphological measurements revealed that all the four Goa specimens are consistent and congruent with the salient morphological descriptions for type specimens of *H. mohnikei* reported by Lourie et al. [14,16,24] for specimens collected from Vietnam and Japan (Table 1). ~~The measured morphometric and meristic characters of Goa specimens are consistent with~~

Formatted: List Paragraph, Indent: First line: 0"

~~the morphological descriptions for *H. mohnikei* reported by Lourie et al. [14,15,23] for specimens collected from Vietnam and Japan.~~

Table 1. Detailed morphology of *H. mohnikei*. Morphological characteristics of four specimens of *Hippocampus mohnikei* collected from the Mandovi estuary, Goa, central west coast of India (MK1 and MK2 = Female; MK3 and MK4 = Male).

	**MK1**	**MK2**	**MK3**	**MK4**
**Morphometric characters (mm)**				
Height (Ht)	39.2	54.2	48.1	51.3
Standard length (SL)	47.3	60.1	55.2	57.2
Trunk length (TrL)	16.1	19.4	15.2	15.1
Tail length (TaL)	24.2	32.1	31.2	33.1
Head length (HL)	8.5	9.1	9.2	8.7
Snout length (SnL)	2.5	3.1	3.3	2.5
HL/SnL ratio	3.4	3.2	3.3	3.5
Snout depth (SnD)	1.5	2.1	2.1	2.1
Head depth (HD)	5.5	7.1	6.9	7.2
Eye orbit	1.5	2.1	2.2	2.1
Trunk depth between 4th and 5th trunk ring (TD4)	3.1	4.2	3.9	4.1
Trunk depth between 9th and 10th trunk ring (TD9)	4.7	6.1	5.8	5.9
Pectoral fin base length	2.1	2.1	2.1	2.2
Dorsal fin base length	3.5	4.1	4.1	3.9
Anal fin base length	0.5	0.5	0.5	0.5
Pouch length	-	-	6.2	6.6
Pouch depth	-	-	3.1	2.5
Pouch width	-	-	3.2	3.3
**Meristic features (numbers)**				
Trunk Rings (TrR)	11	11	11	11
Tail Rings (TaR)	37	38	37	39

TrR supporting dorsal fin	2	2	2	2
TaR supporting dorsal fin	1	1	1	1
Cheek spines	2	2	2	2
Eye spines	2	2	2	2
Enlargement of trunk Rings	1, 4, 7, 11	1, 7, 10	1, 4, 7, 11	1, 4, 7, 10
Enlargement of tail Rings	4, 7, 10, 13	4, 8, 10, 13	4, 7, 9, 13	4, 7, 10, 14
Dorsal fin rays	15	16	16	16
Pectoral fin rays	12	14	14	14
Anal fin rays	4	4	4	4

Comparative morphology

We ~~A~~ compared ~~ative~~ the morphological features of *H. mohnikei* with ~~and~~ other two commonly occurring seahorse species, ~~(~~*H. kuda* and *H. trimaculatus*~~)~~ (Table 2). Fresh specimens of *H. kuda* was orange to dark brown with reddish spots over the body with single eye and cheek spine, rounded and backward curled coronet and deeper head and rounded or less developed body spines [14,15]. *H. trimaculatus* has three prominent black spots on the dorso-lateral surface of the first, fourth and seventh trunk rings [14,15] which are clearly absent in *H. mohnikei*. Single eye and hooked like cheek spine also observed in *H. trimaculatus*. Snout length (SnL) of *H. mohnikei* was shorter compared to the *H. kuda* and *H. trimaculatus*. These morphological characters clearly distinguishe~~s~~ *H. mohnikei* from *H. kuda* and *H. trimaculatus*.

PCA used to elucidate the factors contributing to morphological differences in *H. mohnikei*, *H. kuda* and *H. trimaculatus* showed that many morphometric and meristic characters play an important role in differentiating the seahorse species. Results of PCA analysis revealed that 11 morphometric and meristic characters integrated into two principal components (PC1 and PC2) which cumulatively contributed to 97% of total variance (Table 3). The PC1 which explained 81% of total variation consisted of morphometric characters

Formatted: Space After: 0 pt, Don't add space between paragraphs of the same style, Line spacing: 1.5 lines

Formatted: Space After: 0 pt, Don't add space between paragraphs of the same style, Line spacing: 1.5 lines

Formatted: Space After: 0 pt, Don't add space between paragraphs of the same style, Line spacing: 1.5 lines

Formatted: Space After: 0 pt, Don't add space between paragraphs of the same style, Line spacing: 1.5 lines

Formatted: Space After: 0 pt, Don't add space between paragraphs of the same style, Line spacing: 1.5 lines

Formatted: Space After: 0 pt, Don't add space between paragraphs of the same style, Line spacing: 1.5 lines

Formatted: Space After: 0 pt, Don't add space between paragraphs of the same style, Line spacing: 1.5 lines

Formatted: Space After: 0 pt, Don't add space between paragraphs of the same style, Line spacing: 1.5 lines

Formatted: Space After: 0 pt, Don't add space between paragraphs of the same style, Line spacing: 1.5 lines

such as SL, TaL, TD4, HL, TD9, TrL, HD, SnL and SnD while, the PC2 which explained 16% of total variation consisted of the TaR and DF. The derived ordination diagram after

**Morphometrics (mm)**	***H. mohnikei* (*n* = 4)**	***H. kuda* (*n* = 10)**	***H. trimaculatus* (*n* = 10)**

plugging standardized morphological data of all individuals of three seahorse species into PCA is depicted in Fig. ~~4~~5. PCA ordination diagram showed that three seahorse species were clustered out distinctly in orientation space. Therefore, based on comparison of morphological traits [14,15,2~~3~~4] and the results of PCA, the identity of seahorse specimens collected from Goa was confirmed as *H. mohnikei*.

Height (Ht)	48.2 ± 6.50^{a}	157.73 ± 19.24^{b}	93.06 ± 14.71^{c}
Standard Length (SL)	54.95 ± 5.48^{a}	182.65 ± 11.64^{b}	109.87 ± 6.45^{c}
Trunk Length (TrL)	16.45 ± 2.02^{a}	47.83 ± 2.54^{b}	26.40 ± 2.12^{c}
Tail Length (TaL)	30.15 ± 4.04^{a}	104.43 ± 9.07^{b}	61.70 ± 3.40^{c}
Head Length (HL)	8.83 ± 0.41^{a}	30.39 ± 1.88^{b}	21.70 ± 1.77^{c}
Snout Length (SnL)	2.67 ± 0.21^{a}	12.06 ± 1.04^{b}	8.90 ± 0.99^{c}
HL/SnL ratio	3.33 ± 0.14^{a}	2.54 ± 0.16^{b}	2.45 ± 0.19^{bc}
Snout Depth	1.95 ± 0.30^{a}	4.25 ± 0.41^{b}	3.45 ± 0.16^{c}
Head Depth	6.68 ± 0.79^{a}	14.80 ± 1.84^{b}	9.50 ± 0.53^{c}
TD4	3.83 ± 0.50^{a}	12.16 ± 1.19^{b}	6.75± 0.86^{c}
TD9	5.63 ± 0.63^{a}	18.68 ± 1.51^{b}	9.55 ± 0.90^{c}
Pectoral fin base length	2.03 ± 0.1^{a}	7.07 ± 1.2^{b}	4.75 ± 0.6^{c}
Dorsal fin base length	3.88 ± 0.3^{a}	15.73 ± 1.8^{b}	9.80 ± 0.8^{c}
**Meristic features (numbers)**			
Trunk Rings (TrR)	11 ± 0.00	11 ± 0.00	11 ± 0.00
Tail Rings (TaR)	38 ± 1.00^{a}	36 ± 1.00^{b}	41 ± 1.00^{c}
TrR supporting Dorsal Fin	2 ± 0.00	2 ± 0.00	2 ± 0.00
TaR supporting Dorsal Fin	1 ± 0.00	1 ± 0.00	1 ± 0.00
Cheek Spines	2 ± 0.00^{a}	1 ± 0.00^{b}	1 ± 0.00^{c}
Eye spines	2 ± 0.00^{a}	1 ± 0.00^{b}	1 ± 0.00^{c}
Dorsal fin rays	16 ± 1.00^{a}	18 ± 1.00^{b}	20 ± 1.00^{c}
Pectoral fin rays	14 ± 1.00^{a}	16 ± 1.00^{b}	17 ± 1.00^{c}

Table 2. Morphological comparison of seahorses. Comparison of morphological characteristics of *H. mohnikei* with other two seahorse species, (*H. kuda* and *H. trimaculatus)*. Sample size for each species are shown in parentheses, Mean±SD denoted with different superscripts in same row significantly differ from each other ($P < 0.05$).

Table 3. PCA analysis of seahorse species. Loadings of components matrix and contributions of each axis to total variance ranked as per their coefficients (eigen values > ± 0.30 are included; values in parentheses indicate variance explained by each principal component).

**Morphological characters**	**Coefficients**
**Principal component 1 (81%)**	
SL	0.334
TaL	0.333
TD4	0.329
TD9	0.327
HL	0.327
TrL	0.325
HD	0.324
SnL	0.318
SnD	0.314
**Principal component 2 (16%)**	
TaR	0.663
DF	0.655

Fig. 5. PCA analysis scores. Scores of factor loadings plot of principal component analysis of three species, *H. mohnikei*, *H. kuda* and *H. trimaculatus* based on morphological data.

Phylogenetic analysis and genetic distance

PCR for the mitochondrial COI and Cyt *b* genes resulted in 609 bp and 639 bp amplicons, respectively. Maximum likelihood (ML) trees were constructed with sequences of COI and Cyt *b* genes of Goa specimens (MK1, MK2, MK3 and MK4) using K2P distances with those of morphologically similar and most abundant seahorse species in the Indo-Pacific region (Fig. ~~5~~6 and Fig. ~~6~~7)~~, respectively~~. Sequences of COI (GenBank accession numbers MK330041.1, MN595216.1, MN595217.1 and MN595218.1) and Cyt *b* (GenBank accession numbers MK112274.2, MN595213.1, MN595214.1 and MN595215.1) genes of Goa specimens showed maximum similarities ~~of 100%~~ with *H. mohnikei* (GenBank accession number: GQ502157.1) and ~~93%~~ with *H. mohnikei* (GenBank accession number: EU179923.1 and EU179924.1), respectively. Based on maximum similarities, the genetic identity of Goa specimens was confirmed as *H. mohnikei*. Genetic distance describes the kinship distance between intraspecies and interspecies. Calculated pair-wise genetic distance (K2P) values based on COI and Cyt *b* sequences of *H. mohnikei* from Goa and other localities (Japan, Korea, China, Vietnam and Thailand) ~~are presented in Table 4 and Table 5, respectively. Relatively, *H. mohnikei* from Goa~~ revealed ~~showed~~ that *H. mohnikei* from Goa the show least genetic distances of 0.7% (COI) and 0.7% (Cyt *b*) with populations of *H. mohnikei* from Vietnam (COI) and Thailand (Cyt *b*), respectively (Table 4 and Table 5).

Fig. ~~5~~6. Phylogenetic relationships of Goa specimens with other seahorse species based on COI gene sequences. ML tree of *Hippocampus mohnikei* and closely related seahorse species from mitochondrial cytochrome oxidase subunit I gene (COI) sequences using K2P distances with 1000 bootstrap iterations. Numbers at nodes indicate bootstrap support values and nodes with >50% bootstrap support are shown. The alligator pipefish, *Syngnathoides biaculeatus* (EF189167.1) served as an out-group species. Scale bar = genetic distance of 0.05. ML = Maximum likelihood

Formatted: Not Highlight

Fig. ~~6~~7. Phylogenetic relationships of Goa specimens with other seahorse species based on Cyt *b* gene sequences. ML tree of *Hippocampus mohnikei* and closely related seahorse species from mitochondrial cytochrome *b* gene (Cyt *b*) sequences using K2P distances with 1000 bootstrap iterations. Numbers at nodes indicate bootstrap support values and nodes with >50% bootstrap support are shown. The alligator pipefish, *Syngnathoides biaculeatus* (EF189167.1) served as an out-group species. Scale bar = genetic distance of 0.05. ML = Maximum likelihood

Formatted: Not Highlight

Table 4. Pair-wise genetic distance based on COI gene sequences. Pair-wise genetic distances (Kimura 2-parameter model) based on COI gene sequences of *Hippocampus mohnikei* populations between Goa and the Indo-Pacific region. Values in parentheses indicate percentage.

**Localities of *H. mohnikei* with GenBank Accession numbers**	**Goa**	**Japan**	**Vietnam**	**China**
Goa Specimens (MN595216–MN595218; MK330041.1)				
Japan (GQ502158.1–GQ502159)	0.008 (0.8)			
Vietnam (GQ502157.1)	0.007 (0.7)	0.009 (0.9)		
China (KP140138–KP140181)	0.010 (1.0)	0.003 (0.3)	0.010 (1.0)	

Table 5. Pair-wise genetic distance based on Cyt *b* gene sequences. Pair-wise genetic distances (Kimura 2-parameter model) based on Cyt *b* gene sequences of *Hippocampus mohnikei* populations between Goa and the Indo-Pacific region. Values in parentheses indicate percentage.

**Localities of *H. mohnikei* with GenBank Accession numbers**	**Goa**	**Japan1**	**Japan2**	**Korea1**	**Korea2**	**China**	**Thailand**
Goa Specimens (MN595213.1, MN595214.1, MN595215.1 and MK112274.2)							
Japan1 (AF192688.1−AF192689.1; KT731898.1−KT731909.1)	0.067 (6.7)						
Japan2 (KX017612.1−KX017625.1)	0.070 (7.0)	0.071 (7.1)					
Korea1 (KT731897.1–KT731920.1; KT731924.1–KT731926.1; KT731929.1; KT731932.1–KT731935.1; KT731940.1–KT731941.1; KT731944.1–KT731952.1 and KT731955.1)	0.066 (6.6)	0.040 (4.0)	0.044 (4.4)				
Korea2 (KT731921.1–KT731923.1; KT731928.1; KT731930.1–KT731931.1; KT731936.1–KT731937.1, KT731943.1 and KT731955.1)	0.067 (6.7)	0.004 (0.4)	0.012 (1.2)	0.066 (6.6)			
China (KT946929.1–KT946937.1; KC527556.1 and KC527584.1)	0.066 (6.6)	0.037 (3.7)	0.042 (4.2)	0.057 (5.7)	0.004 (0.4)		
Thailand (EU179923.1–EU179924.1)	0.007 (0.7)	0.001 (0.1)	0.001 (0.1)	0.001 (0.1)	0.001 (0.1)	0.001 (0.1)	

Ocean circulation

The climatological currents generated for the winter season (December to February) from 2007 to 2017 for the Indo-Pacific region are depicted in Fig. ~~7~~4. As seen from the figure that the circulation shows a large-scale flow moving from the East China Sea to the South China Sea. From the South China Sea, the major part of the flow turns southeastward entering into the Java Sea and a small part turns northwestward to eastern Bay of Bengal (BoB) and the Andaman Sea through Malacca Strait. From eastern BoB, the surface waters flow westward as a part of the north equatorial current (NEC). On encountering the coast of Sri Lanka, a part of the NEC move northward into the northern BoB, while the rest moves westward and into the southern Arabian Sea, south of Sri Lanka. The NEC feeds into the wintertime west India coastal current (WICC) and moves waters from NEC towards north into the Arabian Sea along its eastern boundary.

Discussion

The geographic ~~distributional~~ range of *Hippocampus mohnikei* is still unresolved as [14,~~15~~16] confirmed the nativity of this species to Japan and Vietnam and likely extending throughout the South China Sea and the Gulf of Thailand [14, ~~1~~16~~5~~,2~~3~~4]. Recent reports on the occurrence of *H. mohnikei* from many other non-native locations in north- and south-eastern coastal waters of Asia [10,3~~5~~6,4~~1~~2,5~~1~~2,5~~6~~7,5~~7~~8] suggest its significantly extended distribution in the Indo-Pacific region (Fig. 8). The sighting of *H. mohnikei* in the Palk Bay, southeast coast of India [10] is till date the known range limit towards northwestern Asian coastal waters. In southeastern Asian coastal waters, *H. mohnikei* has been reported from Singapore [5~~7~~8] as well as from Cambodia, Malaysia, Thailand and Vietnam [3~~5~~6]. In northeast Asia, *H. mohnikei* is reported from China [43,56] and South Korea [61,63].

~~Towards northeast Asian coastal waters [56,58] reported *H. mohnikei* in southern Korea, whereas, [41,51] reported *H. mohnikei* from China.~~

Fig. 8. Reported sightings of *H. mohnikei* in Indo-Pacific region. Map showing native range, recent sightings and all reported sightings for *Hippocampus mohnikei* in the Indo-Pacific region. Sightings are classified as new (this study, ★), recently published (2004–2016, (▲) and original records [14] (*). Map was modified from Aylesworth et al. [3~~5~~6] and re-created in the World Geodetic System (WGS) 84 datum.

The present report on the occurrence of *H. mohnikei* in a bay-estuarine system of Goa, CWCI as confirmed by both morphological and ~~-~~molecular analysis ~~is the most recent~~ represents a significant westward expansion compared to the previously known geographic ~~distributional~~ range of this species [3~~5~~6]. Since the reporting of *H. mohnikei* solely based on morphological characters from the Palk Bay, southeast coast of India [10], no further sightings of this species have been reported from coastal marine waters of India. Therefore, a comparison of genetically distinct populations of *H. mohnikei* inhabiting native (Japan and Vietnam) and extended ranges in the Indo-Pacific region (Fig. 8) with Goa specimens gains significance. Lourie et al. [~~7~~15,2~~3~~4] reported that a Vietnamese specimen of *H. mohnikei* was genetically distinct from *H. mohnikei* from Japan. ~~Results of p~~. ML tree constructed with sequences of COI gene of Goa specimens (GenBank accession numbers, MN595216.1, MN595217.1, MN595218.1 and MK330041.1) using K2P distances showed maximum similarity with GenBank accession number, GQ502157.1 from Vietnam. On the other hand, ML tree constructed with sequences of Cyt *b* gene of Goa specimens (GenBank accession numbers, MN595213.1, MN595214.1, MN5952115.1 and MK112274.2) using K2P distances showed maximum similarities with *H. mohnikei* from Thailand (GenBank accession numbers,

EU179923.1 and EU179924.1). Furthermore, a comparison of pair-wise genetic distance values (K2P) based on sequences COI (Table 4) and Cyt *b* (Table 5) genes showed that Goa specimens showed least genetic distance with populations of *H. mohnikei* from Vietnam (K2P value, 0.7%) and Thailand (K2P value, 0.7%), respectively. In contrast, high pair-wise K2P values of sequences of Cyt *b* between *H. mohnikei* populations from the Gulf of Thailand and those from China (13.54−14.46%) and Japan (14.42−14.79%) have been recently reported [6059]. A comparative assessment of K2P values of the present study with those from Vietnam [61] and Gulf of Thailand [5960], respectively for COI and Cyt *b* suggests that *H. mohnikei* populations from Goa, Vietnam and the Gulf of Thailand might be genetically distinct from China and Japan. Also, a possible genetic differentiation in populations of *H. mohnikei* inhabiting coastal marine waters of northeastern Asia (Japan, Korea and China) and southeastern Asia (Vietnam, Thailand, Cambodia, Malaysia, Singapore and India) may not be ruled out. Furthermore, least K2P values between different populations of *H. mohnikei* disjunct populations of *H. mohnikei* (Goa vs Vietnam, COI gene) and (Goa vs. Thailand, Cyt *b* gene) may reflect on the possibility of cryptic species within *H. mohnikei* populations [7,234,60, 6159] which needs further confirmation.

Recent studies have advanced our understanding of on habitat associations of *H. mohnikei* compared to what has been described in the IUCN Red List Assessment [424]. Previously, *H. mohnikei* was reported only in association with seagrass beds in Japan and in an estuary in Vietnam [15,234]. Association of *H. mohnikei* populations with seagrass beds (*Zostera* spp.) in Japan [5958,602], Korea [567] and China [412] has been reported. Whereas the specimen reported from the Palk Bay [10] was associated with two other additional genera of seagrass (*Halophila* and *Thalassia*). Recently, published data report on the habitat preferences of *H. mohnikei* from Southeast Asia [356] indicate its association with seagrass beds of *Cymodocea* spp., *Enhalus* spp., *Halodule* spp., *Halophila* spp., *Syringodium* spp. and *Thalassia* spp.

Furthermore, these authors also report the association of *H. mohnikei* with mangroves, oyster beds, mussel farms and sandy beaches, whereas, [5~~7~~8] has reported ~~the~~ its occurrence in association with seaweeds in Singapore. From these studies, it is evident that *H. mohnikei* occupies a wide variety of habitats.

All previous reports on the habitat descriptions of *H. mohnikei* were located in the coastal waters and bay-estuarine systems in the depth range of 0–10 m [10, ~~1~~16~~5~~,2~~3~~4,3~~5~~6,4~~1~~2,5~~6~~7]. Interestingly, the occurrence of small juveniles of *H. mohnikei* in association with highly dense live prey organisms in the offshore waters of Japan has also been reported [3~~7~~8]. The present report of *H. mohnikei* is based on specimens caught as an incidental catch in bag nets fixed to the stakes operated in the Mandovi estuary, Goa. Although precise information on the habitat of *H. mohnikei*, could not be ascertained, it is worth describing the surrounding environs at the site of collection. Predominantly, the estuarine stretches of the River Mandovi have dense mangrove coverage and extensive oyster beds [6~~1~~3] and patches of seagrass, *Halophila ovalis* and *H. beccarii* [6~~2~~4]. The channel network of Mandovi estuary is characterised by islands (Chorao, Divar and Vanxim) and many sheltered areas interspersed with dense mangrove ecosystem. The present occurrence of *H. mohnikei* in a bay-estuarine system of Goa is not quite surprising as these are the typical habitats where this species has been previously reported. For ascertaining habitat shift by geographically disconnected ~~disjunct~~ populations of *H. mohnikei*,. further intense studies on habitat specificities of different life stages therefore, are warranted.

Food availability has been considered as one of the factors influencing the abundance and distribution of seahorses [20~~19~~]. Zooplankton such as *Oithona davisae*, Calanoida and *Penilia avirostris* have been identified as the main prey organisms for juvenile and young *H. mohnikei* in Tokyo Bay [3~~7~~8], whereas the adults have been reported to prey upon mainly larger organisms such as gammarid and caprellid amphipods [5~~8~~9]. Nutrient-rich waters of

the Mandovi estuarine system sustain high primary productivity and abundance of zooplanktonic prey organisms such as copepods [653−657] which are essential for the survival and establishment of seahorse populations. Considering the prevailing environmental conditions, mangrove shelter and abundance of prey organisms, the likelihood of establishment and colonization of seahorse populations in these sheltered areas is quite high.

Factors such as habitat, diet, and even anthropogenic activities are the main reasons cited for the migration of seahorses [668]. Furthermore, instances of seahorses for long distance dispersal by means of rafting along with seaweeds or any other suitable holdfast under the influence of ocean currents during juvenile stages have been documented [2,201]. Southward migration of *H. histrix* ~1800 km under the influence of East Australian Current in the Great Barrier Reef has been reported [312]. Furthermore, ~1100 km migration from the south to the northeast coast of India by *H. fuscus* under the influence of East Indian Coastal Current has been also reported [334]. Direct evidence for passive long-distance migration of *H. trimaculatus* at Malacca Strait has been documented recently [345].

Newborn juveniles of many seahorse species undergo a pelagic phase (planktonic) ranging between two and six weeks depending on the species before they settle down into sessile habitats attain a settlement phase (benthios)c) [2,679]. Consequently, planktonic phase juveniles may facilitate in widespread gene flow across geographically disjunct disconnected populations and resulting in genetic homogeneity [70]. The occurrence of small juveniles of *H. mohnikei* (15.1−45.5 mm) in association with highly dense live prey organisms in offshore waters of Japan [378] suggests that *H. mohnikei* might undergo a prolonged planktonic phase. Furthermore, a seasonal (warm and cold season) inshore-offshore migration by *H. mohnikei* populations inhabiting Chinese coast has been confirmed [512]. Higher dispersal capabilities of seahorse juveniles with floating debris, prey swirls and with seaweed rafts than adults has been reported [2,289,345]. It has been postulated that marine species with greater longevity,

free swimming and feeding (planktotrophic) larval phases have relatively ~~a~~ high dispersal abilities and thus permit ~~romoting~~ genetic exchange between populations [70~~69~~]. A relatively prolonged planktonic phase as in case of *H. mohnikei* may facilitate the high degree of dispersal under the influence of oceanic circulation.

The confirmed occurrence of four sub-adult specimens (Mandovi estuary) and suspected occurrence of an adult specimen of *H. mohnikei* in the proximately adjacent bay-estuarine ecosystem (Chapora estuary) of Goa, CWCI could be ~~as~~ a consequence of either long-distance dispersal from Southeast Asian countries or stepping-stone mediated dispersal as revealed by oceanic currents circulation in the Indo-Pacific region. It is clearly seen from Fig. 7 that water mass from southeastern Asian coasts and east coast of India enters into the Arabian Sea as a result of winter circulation [7~~0~~1]. In the absence of geographical barriers between *H. mohnikei* populations of Goa and Southeast Asian countries, ocean currents flowing from regions where sightings of *H. mohnikei* may contribute to wide dispersal. Therefore, it is plausible that oceanic currents may be aiding long-distance or stepping stone dispersal of *H. mohnikei* populations from different geographical locations such as Cambodia, Malaysia, Thailand and Vietnam [14,15,3~~5~~6,61~~59~~], Singapore [5~~7~~8] and the Palk Bay, India [10] to the west coast of India.

Conversation with local fishermen who operate stake nets at the collection site revealed that sightings of seahorses are relatively more common during summer monsoon season than in non-monsoonal months. During the summer monsoon period, a number of rivulets and rivers connected to the Mandovi estuarine network discharge large amounts of freshwater [73]. Consequently, it may be possible that the rainwater discharge from sheltered areas (the preferred seahorse habitats) may force ~~entail~~ seahorses ~~to flow~~ downstream along with currents due to their poor swimming abilities. It is well known that the west coast of India comes under the influence of substantial coastal cold-water upwelling event during the

summer monsoon season [4~~5~~6,7~~1~~3]. It has been reported that the survival and dispersal of tropical marine species are known to be impacted by a drastic decrease in water temperature associated with upwelling [7~~2~~4–75]. Consequently, seahorse populations in western coastal India might undergo seasonal fluctuations [2~~0~~1]. A significant decrement in abundance and diversity of demersal fishes during the summer monsoon in a bay-estuarine system of the Mandovi river has been documented [4~~7~~8]. As a consequence of the reduction in salinity and drop in temperature associated with monsoonal precipitation and upwelling, respectively the seahorse populations might face limitations in growth and recruitment along the west coast of India. Interestingly, unlike most subtropical seahorse species, the greater tolerance by native populations of *H. mohnikei* to cold water ~~inhabiting~~ in the Western Pacific has been reported [30~~29~~]. However, further temperature stress simulation studies are required to ascertain whether geographically disconnected ~~translocated~~ populations of *H. mohnikei* also possess a similar trait or not.

Conclusions

Recognising the potential of seahorses as a popular flagship species in estuarine conservation and sustainable management [1,7~~5~~7,7~~6~~8], significant westward range expansion of *H. mohnikei* is of considerable biogeographic and conservation importance. Further intense sampling and analysis of *H. mohnikei* populations along the Indian coast are therefore required to confirm whether the extension of their natural range based on this new information ~~present occurrence of *H. mohnikei* in a bay-estuarine system, CWCI~~ represents ~~(i) an isolated translocation or~~ (i~~i~~) naturally occurring facilitated by oceanic circulation or (iii) the first sighting of an unreported of fairly established populations of this vulnerable seahorse species.

Formatted: Font: Not Italic

Formatted: Font: Not Italic

Formatted: Font: Not Italic

Acknowledgements

The authors are grateful to the Director, CSIR-National Institute of Oceanography, Goa (India) for encouragement and facilities. Thanks are due to local fishermen Mr. Harish Halarnkar for providing seahorse specimens. Thanks are also due to Dr. Mani Murali and Mr. Devanand Kavlekar for their help in preparing the maps. We would like to deeply thank Dr. Qiang Lin for sharing the morphometric data of *Hippocampus mohnikei* for comparison. The authors are thankful to Drs. Graham Short and Riley Pollom and two anonymous reviewers for their critical and constructive comments for improving the quality and presentation of the manuscript. This represents contribution No. xxxx of the CSIR-National Institute of Oceanography, Goa (India).

Formatted: Font: (Default) Times New Roman, 12 pt, Font color: Auto, Pattern: Clear

Formatted: Font: Italic

Formatted: Font: Not Italic

Author Contributions

Conceived and designed the experiments: SVS RAS RDSK: Performed the experiments: SVS RAS RDSK MSS HTK. Analyzed the data: SVS RAS MSS RDSK JN. Contributed reagents/materials/analysis tools: JN HTK. Wrote the paper: RAS SVS RDSK.

References

1. Sreepada RA, Desai UM, Naik S. The plight of Indian seahorses: Need for conservation and management. Curr Sci 2002; 82: 377–378.
2. Foster SJ, Vincent ACJ. Life history and ecology of seahorses: implications for conservation and management. J Fish Biol 2004; 65: 1–61.
3. Kuiter RH. Seahorses and Their Relatives. Aquatic Photographics, Seaford, Australia; 2009.
4. Froese R, Pauly D. FishBase.www.fishbase.org. Downloaded on 10/2018.

5. Harasti D, Martin-Smith K, Gladstone W. Ontogenetic and sex-based differences in habitat preferences and site fidelity of White's seahorse *Hippocampus whitei*. J Fish Biol 2014; 85: 1413–1428.

6. Hellyer C, Harasti D, Poore A. Manipulating artificial habitats to benefit seahorses in Sydney Harbour, Australia. Aquat Conserv Mar Fresh Ecosyst 2011; 21: 582–589.

7. IUCN Seahorse, Pipefish and Stickleback Specialist Group (2019). https://www.iucn-seahorse.org (downloaded on 01/11/2019).

~~7. Lourie SA, Pollam RA, Foster SJ. A global revision of the seahorses *Hippocampus* Rafinesque 1810 (Actinopterygii: Syngnathiformes): taxonomy and biogeography with recommendations for further research. Zootaxa 2016; 4146: 1–66.~~

8. Salin KR, Mohankumaran NC. Resources and biodiversity of seahorses and the need for their conservation in India. Aquacult Asia 2006; 10: 3–8.

9. Murugan A, Dhanya S, Rajagopal S, Balasubramanian T. Seahorses and pipefishes of the Tamil Nadu coast. Curr Sci 2008; 95: 253–260.

10. Thangaraj M, Lipton AP. Occurrence of the Japanese seahorse *Hippocampus mohnikei* Bleeker 1854 from the Palk Bay coast of southeastern India. J Fish Biol 2007; 70: 310–312.

11. Krishnan TS, Kuiter RH, Patterson J. Occurrence of western Indian Ocean seahorse, *Hippocampus borboniensis* Dumeril, 1870, in the Gulf of Mannar, Southeastern India. Ind J Geo-Mar Sci 2011; 40: 642–644.

12. Yogeshkumar JS, Geetha S. A new species of *Hippocampus montebelloensis* (Family: Syngnathidae) from the southeast coast of India. Int J Plant Anim Env Sci 2012; 2: 56–58.

13. Subburaman S, Murugan A, Goutham S, Kaul R, Prem Jyothi PVR, Balasubramanian T. First distributional record of giraffe seahorse, *Hippocampus camelopardalis* Bianconi

1854 (Family Syngnathidae) from Gulf of Kachchh waters, north west coast of India. Indian J Geo-Mar Sci 2014; 43: 408–411.

14. Lourie SA, Foster SJ, Cooper EWT, Vincent ACJ. A Guide to the Identification of Seahorses. Project Seahorse and TRAFFIC North America; 2004.

15. Lourie SA, Pollam RA, Foster SJ. A global revision of the seahorses *Hippocampus* Rafinesque 1810 (Actinopterygii: Syngnathiformes): taxonomy and biogeography with recommendations for further research. Zootaxa 2016; 4146: 1–66.

1~~5~~6. Lourie SA, Vincent ACJ, Hall HJ. Seahorses: an identification guide to the world's species and their conservation. Project seahorse, London, UK; 1999.

1~~6~~7. Silveira RB, Siccha-Ramirez R, Silva JRS, Oliveira C. Morphological and molecular evidence for the occurrence of three *Hippocampus* species (Teleostei: Syngnathidae) in Brazil. Zootaxa 2014; 3861: 317–332.

1~~7~~8. Casey SP, Hall HJ, Stanley HF, Vincent ACJ. The origin and evolution of seahorses (genus *Hippocampus*): a phylogenetic study using the cytochrome b gene of mitochondrial DNA. Mol Phylogenet Evol 2004; 30: 261–272.

1~~8~~9. Wang X, Han X, Zhang Y, Liu S, Lin Q. Phylogenetic analysis and genetic structure of the seahorse, *Hippocampus fuscus* from the Arabian and Red Sea based on mitochondrial DNA sequences. Mitochondrial DNA A 2019; 30: 165–171.

~~19~~20. Lourie SA, Green DM, Vincent ACJ. Dispersal, habitat differences and comparative phylogeography of South East Asian seahorses (Syngnathidae: *Hippocampus*). Mol Ecol 2005; 14: 1073–1094.

2~~0~~1. Teske PR, Hamilton H, Palsboll PJ, Choo CK, Gabr H, Lourie SA. et al. Molecular evidence for long-distance colonization in an Indo-Pacific seahorse lineage. Mar Ecol Prog Ser 2005; 286: 249–260.

2~~1~~2. Boehm JT, Waldman J, Robinson JD, Hickerson MJ. Population genomics reveals seahorses (*Hippocampus erectus*) of the western mid-Atlantic coast to be residents rather than vagrants. PloS One 2015; 10: e0116219. http://dx.doi.org/10.1371/journal.pone.0116219.

2~~2~~3. Saarman NP, Louie KD, Hamilton H. Genetic differentiation across eastern Pacific oceanographic barriers in the threatened seahorse *Hippocampus ingens*. Conserv Genet 2010; 11: 1989–2000.

2~~3~~4. Lourie SA, Pritchard JC, Casey SP, Ky TS, Hall HJ. et al. The taxonomy of Vietnam's exploited seahorses (family Syngnathidae). Biol J Linn Soc 1999; 66: 231–256.

2~~4~~5. Woodall LC, Otero-Ferrer F, Correia M, Curtis JMR, Garrick Maidment N. et al. A synthesis of European seahorse taxonomy, population structure, and habitat use as a basis for assessment, monitoring and conservation. Mar Biol 2018; 165: 1–19.

2~~5~~6. Curtis JMR, Vincent ACJ. Life history of an unusual marine fish: survival, growth and movement patterns of *Hippocampus guttulatus* Cuvier 1829. J Fish Biol 2006; 68: 707–733.

2~~6~~7. Caldwell I, Vincent ACJ. A sedentary fish on the move: effects of displacement on long-snouted seahorse (*Hippocampus guttulatus* Cuvier) movement and habitat use. Environ Biol Fish 2013; 96: 67–75.

2~~7~~8. Correia M, Caldwell IR, Koldewey HJ, Andrade JP, Palma J. Seahorse (Hippocampinae) population fluctuations in the Ria Formosa Lagoon, south Portugal. J Fish Biol 2015; 87: 679– 690.

2~~8~~9. Luzzatto DC, Estalles ML, Diaz De Astarloa JM. Rafting seahorses: the presence of juvenile *Hippocampus patagonicus* in floating debris. J Fish Biol 2013; 83: 677–681.

30~~29~~. Han SY, Kim JK, Tashiro F, Kai Y, Yoo JT. Relative importance of ocean currents and fronts in population structures of marine fish: a lesson from the cryptic lineages of the *Hippocampus mohnikei* complex. Mar Biodiv 2019; 49: 263–275.

3~~0~~1. Luzzatto DC, Pujol MG, Figueroa D, Diaz De Astarloa JM. The presence of the seahorse *Hippocampus patagonicus* in the deep waters: additional evidence of the dispersive capacity of the species. Mar Biodivers Rec 2014; 7: e71.

3~~1~~2. Harasti D. Range extension and first occurrence of the thorny seahorse *Hippocampus histrix* in New South Wales, Australia. Mar. Biodivers Rec 2015; 8: https://doi.org/10.1017/S1755267215000263.

3~~2~~3. Harasti D. Southward range extension of the great seahorse (*Hippocampus kelloggi* Jordan and Snyder, 1901) in Australia. J Appl Ichthyol 2017; 1–3 https://doi.org/10.1111/jai.13414.

3~~3~~4. Mahapatro D, Mishra RK, Panda S. Range extension of a vulnerable Sea horse *Hippocampus fuscus* (Actinopterygii: Syngnathidae) on the north-eastern Bay of Bengal coast. Mar Biodivers Rec 2017; 10:6. DOI 10.1186/s41200-017-0108-z.

3~~4~~5. Wong NLWS, The JC. Direct observation of drifting juvenile seahorse *Hippocampus trimaculatus* (Leach, 1814) beneath the offshore surface at Malacca Strait. Check List 2017; 13: 993–996.

3~~5~~6. Aylesworth L, Lawson LM, Laksanawimol P, Ferber P, Loh TL. New records of the Japanese seahorse *Hippocampus mohnikei* in Southeast Asia lead to updates in range, habitat and threats. J Fish Biol 2016; 88: 1620–1630.

3~~6~~7. Masuda H, Amaoka K, Araga C, Uyeno T, Yoshino T. The Fishes of the Japanese Archipelago. Tokyo: Tokai University Press; 1984.

3~~7~~8. Kanou K, Kohno H. Early life history of a seahorse, *Hippocampus mohnikei,* in Tokyo Bay Japan. Ichthyol Res 2001; 48: 361–368.

3~~8~~9. Suda Y, Inoue T, Nakamura M, Masuda N, Doi H, Murai T. Nearshore ichthyofauna in the intermediate sandy beach, Doigahama Beach, Yamaguchi Prefecture, Japan. J Nat Fish Univ 2004; 52: 11–29.

40~~39~~. Laksanawimol P, Petpiroon S, Damrongphol P. Trade of seahorses, *Hippocampus* spp. (Actinopertygii: Syngnathiformes: Syngnathidae), on the East Coast of the Gulf of Thailand. Acta Ichthyol Piscata 2013; 3: 229–235.

4~~0~~1. Chang CH, Jang-Liaw NH, Lin YS, Fang YC, Shao KT. Authenticating the use of dried seahorses in the traditional Chinese medicine market in Taiwan using molecular forensics. J Food Drug Analy 2013; 21: 310–316.

4~~1~~2. Zhang Y, Pham NK, Zhang H, Lin J, Lin Q. Genetic variations in two seahorse species (*Hippocampus mohnikei* and *Hippocampus trimaculatus)*: evidence for Middle Pleistocene population expansion. PloS One 2014; 9: e105494.

4~~2~~3. Pollom R. *Hippocampus mohnikei*. The IUCN Red List of Threatened Species 2017: http://dx.doi.org/10.2305/IUCN.UK.20173.RLTS.T41005A54907304.en43. Downloaded on 10 November 2018.

4~~3~~4. Shetye SR, Gouveia AD, Singbal SY, Naik CG, Sundar D. et al. Propagation of tides in the Mandovi–Zuari estuarine network. Proc Indian Acad Sci (Earth Planet Sci) 1995; 104: 667–682.

4~~4~~5. Qasim SZ, Sen Gupta R. Environmental characteristics of the Mandovi-Zuari estuarine system. Estuar Coast Shelf Sci 1981; 13: 557–578.

4~~5~~6. Madhupratap M, Nair KNV, Gopalkrishnan TC, Haridas P, Nair KKC. et al. Arabian sea oceanography and fisheries of the west coast of India. Curr Sci 2001; 81: 355–361.

4~~6~~7. Ansari ZA, Sreepada RA, Dalal SG, Ingole BS, Chatterji A. Environmental influences on the trawl catches in a bay-estuarine system of Goa, west coast of India. Estuar Coast Shelf Sci 2003; 56: 503–515.

4~~7~~8. Ansari ZA, Chatterji A, Ingole BS, Sreepada RA, Rivonker CU. et al. Structure of inshore demersal fish community at Goa. Estuar Coast Shelf Sci 1995; 41: 593–610.

4~~8~~9. Lourie S. Measuring seahorses. Project Seahorse Technical Report No.4, Version 1.0. Project Seahorse, Fisheries Centre, University of British Columbia; 2003.

50~~49~~. Underwood AJ. Experiments in Ecology: their logical and interpretation using Analysis of Variance. Cambridge University Press. Cambridge, UK; 1997.

5~~0~~1. Kumar R Singh PJ, Nagpure NS, Kushwaha B, Srivastava SK. et al. A non-invasive technique for rapid extraction of DNA from fish scales. Ind J Exp Biol 2007; 45: 992–997.

5~~1~~2. Qin G, Zhang Y, Ho ALFC, Zhang Y, Lin Q. Seasonal distribution and reproductive strategy of seahorse. ICES J Mar Sci 2017; 74: 2170–2179.

5~~2~~3. Lourie SA, Vincent ACJ. A marine fish follows Wallace’s line: the phylogeography of the three-spot seahorse (*Hippocampus trimaculatus*, Syngnathidae, Teleostei) in Southeast Asia. J Biogeogr 2004; 31: 1975–1985.

5~~3~~4. Altschul SF, Gish W, Miller W, Myers EW, Lipman DJ. Basic local alignment search tool. J Mol Biol 1990; 215: 403–410.

5~~4~~5. Kumar S, Stecher G, Tamura K. MEGA7: Molecular Evolutionary Genetics Analysis, version 7.0 for bigger datasets. Mol Biol Evol 2016; 33: 1870–1874.

5~~5~~6. Kimura M. A simple method for estimating evolutionary rates of base substitutions through comparative studies of nucleotide sequences. J Mol Evol 1980; 16: 111–120.

5~~6~~7. Choi Y, Rho S, Park H, Kang D. Population characteristics of two seahorses, *Hippocampus coronatus* and *Hippocampus mohnikei*, around seagrass beds in the southern coastal waters of Korea. Ichthyol Res 2012; 59: 235–241.

5~~7~~8. Lim K. New Singapore record of the seahorse, *Hippocampus mohnikei*. Singapore Biodiver Rec 2013; 2013: 68–69.

5~~8~~9. Kwak S, Huh S, Kim H. Feeding habits of *Hippocampus mohnikei* in an eelgrass (*Zostera marina*) bed. Korean J Ichthyol 2008; 20: 112–116.

60~~59~~. Panithanarak T, Karuwanjaroen R. A preliminary study of genetic differentiation in the Japanese seahorse (*Hippocampus mohnikei*) populations: a possibility of cryptic species. Burapha Sci J 2019; 24: 124–137.

61. Hou F., Wen L, Peng C, Guo J. Identification of marine traditional Chinese medicine dried seahorses in the traditional Chinese medicine market using DNA barcoding. Mitochondrial DNA A 2018; 29(1):107–112.

6~~0~~2. Otsuka Y, Suzuki H, Akagawa I. Occurrence, gonad morphology and maturity of Japanese seahorse *Hippocampus mohnikei* in Matsushima Bay, Japan. J School Mar Sci Tech Tokai Univ 2009; 7: 11–22.

6~~1~~3. Wafar S. Ecology of mangroves along the estuaries of Goa; Ph. D. Thesis, Karnataka University, Dharward, India; 1987.

6~~2~~4. Jagtap TG. Distribution of seagrasses along Indian coast. Aquac Bot 1991; 40: 379–386.

6~~3~~5. Padmavati G, Goswami SC. Zooplankton ecology in the Mandovi-Zuari estuarine system of Goa, west coast of India. Ind J Mar Sci 1996; 25: 268–273.

66. Dalal SG, Goswami SC. Temporal and ephemeral variations in copepod community in the estuaries of Mandovi and Zuari– west coast of India. J Plankton Res 2001; 23: 19–26.

6~~5~~7. Das PB. Zooplankton assemblages in spatial stretches of a tropical estuary. Int J Res Appl Sci Engg Technol 2017; 5: 1789–1799.

6~~6~~8. Qin G, Johnson C, Zhang Y, Zhang H, Yin J, Miller G. et al. Temperature-induced physiological stress and reproductive characteristics of the migratory seahorse *Hippocampus erectus* during a thermal stress simulation. Biol Open 2018; 25: 7(6).bio032888.

679. Scales H. Advances in the ecology, biogeography and conservation of seahorses (genus *Hippocampus*). Prog Phys Geog 2010; 34: 443–458.

70. Bohonak AJ. Dispersal, gene flow, and population structure. Q Rev Biol 1999; 74: 21–45.

71. Palumbi SR. Genetic divergence, reproductive isolation, and marine speciation. Annu. Rev Ecol Evol S 1994; 25: 547–572.

702. Wyrtki K. Physical oceanography of the southeast Asian waters. Scientific results of maritime investigations of the South China Sea and the Gulf of Thailand 1959–1961. Naga Report, 2. Scripps Institution of Oceanography, La Jolla, CA; 1961.

713. Shetye SR, Gouveia AD, Shenoi SSC, Michael GS, Sundar D, Almedia AM. et al. The coastal current off western India during the northeast monsoon. Deep-Sea Res 1991; 38: 1517–1529.

724. Fleminger A. The Pleistocene equatorial barrier between the Indian and Pacific oceans and a likely cause for Wallace's Line. In Pierrot-Bults AC, van der Spoel S, Zahuranec BJ, Johnson RK editors, Pelagic marine science. UNESCO Tech Pap Mar Sci 1986; 49: 84–97.

735. Maree RC, Whitfield AK, Booth AJ. Effect of water temperature on the biogeography of South African estuarine fishes associated with the subtropical/warm temperate subtraction. S Afr J Sci 2000; 96: 184–188.

746. Bowen BW, Bass AL, Rocha LA, Grant WS, Robertson DR. Phylogeography of the trumpetfishes *(Aulostomus)*: ring species complex on a global scale. Evolution 2001; 55: 1029–1039.

757. Martin-Smith KM, Vincent ACJ. Seahorse declines in the Derwent estuary, Tasmania, in the absence of fishing pressure. Biol Conserv 2005; 123: 533–545.

7~~6~~8. Shokri MR, Gladstone W, Jelbart J. The effectiveness of seahorses and pipefish (Pisces: Syngnathidae) as a flagship group to evaluate the conservation value of estuarine seagrass beds. Aquat Conserv 2009; 19: 588–595.

Supporting Information

S1 Fig. Picture of *H. mohnikei* posted on social media. Male specimen of *Hippocampus mohnikei* caught by gill net in the Chapora estuary, Goa, India

S1 Table. The COI and Cyt *b* gene sequences. GenBank accession numbers and sources of the mitochondrial gene sequences (COI and Cyt *b*) of seahorse species used for construction of phylogenetic trees.

kResponse to Comments/suggestions made by the Reviewers

Manuscript ID number: PONE-D-19-19415

Manuscript title: Morphological and molecular evidence for range extension and first occurrence of the Japanese seahorse, *Hippocampus mohnikei* (Bleeker 1853) in a bay-estuarine system of Goa, central west coast of India

Authors: Sushant Sanaye, Rakhee Khandeparker, Rayadurga Anantha Sreepada, Mamatha Singanahalli Shivaramu, Harshada Kankonkar, Jayu Narvekar, Mukund Gauthankar

We are thankful for the reviewers for their constructive criticisms/suggestions and their input has substantially improved the quality of the manuscript.

Below, we provide a point-by-point response as to how the feedback received from the Reviewers has been incorporated in the revised manuscript.

This manuscript is a resubmission of manuscript PONE-D-19-19415 with our response to the questions made by the reviewers. The Academic Editor handling for this manuscript is Dr. Rui Rosa

Reviewer #1:

Morphological and molecular evidence for range extension and first occurrence of the Japanese seahorse, *Hippocampus mohnikei* (Bleeker 1853) in a bay-estuarine system of Goa, central west coast of India

The authors report of a seahorse, *Hippocampus mohnikei* usually found in the east Indian and Pacific ocean, recorded for the first time on the west coast of India. Overall, the study is interesting in its findings of range expansion of a small seahorse.

Response: We thank the Reviewer for the excellent review and the positive comments on the manuscript. Constructive suggestions provided by the Reviewer are appreciated.

General comments:

Comment: I think the Authors did not read the Guidelines for submission?. Some sections are messy with Table captions missing or wrongly placed, Fig captions interrupting main text of MS. It was hard to follow and I put it away many times and nearly did not review!. Someone needs to check if all uploaded files are correct before sending to Reviewers. Regarding the manuscript I would urge the authors to separate Tables and Figures with their captions from the main text of the MS. Figure legends/captions occurred within the text and disrupted the flow. Example: Table 2 starts on page 13 without Table legend/caption followed by text (new results). The actual table was (I assume) on page 14 without a header and table legend was below the table!

Response: At the outset, we apologise for the inconvenience caused due to the formatting issues. Here we state that the all the guidelines/instructions for online submission of the manuscript were meticulously adhered to. According to journal requirement, we have again checked our revised manuscript to meet PLOS ONE’s style requirements. Following are the Instructions, under the 'Submission Guidelines' pertaining to Figure Legends (https://journals.plos.org/plosone/s/submission-guidelines) which are reproduced below"

Other elements

- *Figure captions are inserted immediately after the first paragraph in which the figure is cited. Figure files are uploaded separately.*
- *Tables are inserted immediately after the first paragraph in which they are cited.*
- *Supporting information files are uploaded separately.*

We hope that the revised version will be the error-free readable format.

Comment: Another point for the morphological measurements; how about indicating the different measurements on one of the images of the seahorse with abbreviations (explained in Fig caption) e.g. see Forsgren & Lowe 2006 (sea dragon morphology). Table 2 would be more compact.

Response: Highly appreciate constructive suggestion of the Reviewer. The measurement of morphological characters and meristic counts of seahorse specimens were carried out as per the standard protocols/procedures of measuring seahorses as outlined in Lourie et al. (1999); Lourie et al (2003) and Lourie et al (2004). These are standard protocols that have been used widely for the purpose of measurement of morphological and meristic characters of seahorse species by many authors. These references have been cited in sub-section, morphological analysis under Section, 'Materials & Methods' which we consider serve the purpose.

Comment: For the molecular work did you extract and sequence mitochondrial DNA (mtDNA) from all 4 individuals found or just from 2 individuals? I thought you caught 4? If you have 4 samples sequenced (COI and cytb) you should show all 4 in the tree not just the one. Where do the other 3 individuals occur in each tree (COI and cytb)? If all or some of the other 3 do not cluster with the voucher *H. mohnikei* then you have to consider other possibilities.

Response: We strongly appreciate the criticism provided by the Reviewer. As suggested by the reviewer, remaining 3 seahorse specimens (MK1, MK2 and MK3) were also sequenced in addition to originally sequenced seahorse specimen (MK4) for both COI and Cyt *b* genes. Sequences generated from 04 seahorse specimens with GenBank accession numbers, MN595216, MN595218, MN595217 and MK330041.1 for COI gene and MN595213, MN595214, MN595215 and MK112274.2 for Cyt *b* genes, respectively corresponding to 4 seahorse specimens (MK1, MK2, MK3 and MK4) were included in phylogenetic analysis (Fig. 6 and Fig. 7). Phylogenetic analysis revealed that all 4 seahorse individuals from Goa clustered together confirming a monophyletic lineage. Sequences generated of COI and Cyt *b* gene from 4 seahorse individuals have been used for phylogenetic analysis in the revised manuscript.

Comment: Some sections need to be deleted especially in Results! Result section is littered with sentences that end in eg '…. presented in Table x' = not in published papers (more like student assignment). Please explain your results and refer to your table/Fig in brackets! Eg. Morphological measurements revealed ….. state your results (Table 1). Check grammar, punctuation in main text.

Response: The results section has been modified to a great extent as suggested by the Reviewer. We have incorporated all grammatical and typographical edits into the revised text. The English language has been polished after incorporating the suggested grammatical and typographical edits into the text.

<u>Specific comments:</u>

<u>Introduction</u>

Query: Line 57: Sentence; Often overlooked by most fishermen, due to their cryptic nature, their ability to camouflage and the sparse distribution restrict biologists to identify their presence in thick coastal marine habitats.

Response: As suggested by the reviewer, the sentence has been corrected.

Query: Line 100: Sentence about using gene markers re-write … sequenced 2 mtDNA gene regions (loci) COI & cyt b ..

Response: The sentence corrected as suggested by the Reviewer.

<u>Materials & Methods</u>

Query: Line126: incidental catch = is that by catch - if so use correct term

Response: In the revised manuscript, the sentence corrected as Dead seahorses landed as incidental catch in bag nets attached to the stakes operated by local fishermen ....

Query: Lines 132-141: Figure captions! Should be on separate sheet!

Response**:** The MS has been prepared as per journal's prescribed format. Accordingly, the tentative probable positioning of the Figures with captions has been shown in the manuscript

Query: Line 175: '.fin from fresh seahorse ..' I thought they were dead seahorses from by catch? Did I miss something?

Response: Actually, we wanted to infer freshly dead seahorses here. In the revised manuscript, this has been corrected as freshly landed dead seahorses' to bring clarity.

Query: Line 203: Seq of Goa specimens GB MK330041.1 and MK112274.2 = 2 seq's submitted - did you not seq 4 individuals? If the other 2 did not provide DNA- seq (were, they degraded?) you need to say this in your M&M! add a Table S1 (suppl) of the sequences (species name, GB accession # Ref) you downloaded for your phylogenetic tree from GB and cite their work.

Response: We are extremely grateful to the Reviewer for pointing out this mistake. As explained above, remaining 3 individual seahorses (MK1, MK2, MK3) were sequenced subsequently following the same protocols as that was followed for initially sequenced seahorse individual i.e. MK4. Sequences generated from all 4 seahorse individuals, MK1, MK2, MK3 and MK4 were submitted at NCBI vide GenBank accession numbers, MN595216, MN595218, MN595217 and MK330041.1 for COI gene and MN595213, MN595214, MN595215 and MK112274.2 for Cyt *b* genes, respectively were included in phylogenetic analysis (Fig. 6 and Fig. 7).

As advised by the reviewer, all the downloaded sequences of *H. mohnikei* for COI and Cyt *b* genes available at GenBank along with their voucher number and references used for constructing phylogenetic trees has been provided Supplementary S1 Table.

Results

Query: Line 236-237: delete sentence! Start explaining your main result and refer to your (Table/Fig) in brackets. ‘Morphological measurements revealed …Goa specimens are consistent with … (Table1). I would move Table 1 into Suppl material (raw data). Then describe some of the more important measurements that support your work. Please don’t repeat results in your text if they are already in Table 1! People can read tables. So explain the measurements eg. on average size of … (mean #) comparable to size of *mohnikei* samples from eg. east India or Thailand/Vietnam? Line 258-259: re-write sentence … we compared … commonly occurring seahorses, *H. kuda* … (Table 2).

Response: As advised by the reviewer, Line, 236-237 has been deleted. Now, the sub section on 'morphological analysis' under Results section has been modified to include only the salient findings. We have modified the Results section to a great extent and hope to have improved it by doing so.

Firstly, we have re-arranged the few paragraphs to better mirror the presentation sequence of the results. Additionally, we have introduced Table 3 "Comparison of diagnostic morphological characters of all vouchered specimens of *Hippocampus mohnikei*” for comprehensiveness of the morphological data. Since, Table 1 describes all the morphological characters of four seahorse specimens collected from Goa coast. Considering its significance to the manuscript, Table 1 has retained as Table 1 in the revised manuscript. All other typos and corrections suggested by the Reviewer have been incorporated in the revised manuscript.

Query: Pages 11-12, and 13-14 totally messy with tables throughout text making it difficult to follow should have been on separate sheet.

Response: We apologise for the inconvenience caused due to formatting issues. The revised manuscript has been checked to meet PLOS ONE’s style requirements.

Query : Page 13-14 = messy table is split I assume? No captions above table header - I assume Table 2? AND Table caption go ABOVE a table not below please read Plos One Author Guidelines!

Response: We apologise for the inconvenience caused due to formatting issues. Now, the revised manuscript has been checked to meet PLOS ONE’s style requirements and hope the formatting issue is resolved.

Query : Table 3 - PCA – move to Suppl material it is already represented in Fig 4 – don’t repeat results.

Response: As suggested by the Reviewer, Table 3 on PCA output has moved to Supplementary material (S2 Table)

Query: Phylogenetic analysis – needs overhaul. Both trees (Fig. 5 & 6) only have 1 goa individual in tree. Where are the other 3 sequences? You need to add all 4 indiv seq to each gene region (COI, cyt b) tree. If by chance other individuals did not fall into same clade as *H mohnikei* – this needs to be discussed! There are probably other reasons for it …(eg. hybrids?). When you aligned the seq to the other GB downloaded seq in MEGA it should be obvious especially COI (Barcode of Life – check out *H mohnikei* on BOLD systems http://www.boldsystems.org/index.php/TaxBrowser_TaxonPage? taxid =66243)

Response: The subsection of 'Phylogenetic analysis' has been modified to a great extent by incorporating the sequence data of all 4 individual seahorses from Goa. Maximum likelihood (ML) trees for both COI

and Cyt *b* genes have been constructed by aligning them with the downloaded sequences for all vouchered *H. mohnikei* downloaded from the GenBank in the revised manuscript. Phylogenetic analysis revealed that all 4 seahorse individuals from Goa clustered together and formed a single clade thus confirming a monophyletic lineage. This has been now mentioned in the revised manuscript. As advised by the reviewer, availability of sequences of COI gene for *H. mohnikei* were also checked in Barcode of Life (BOLD Systems).

Query: Line 317 – delete 'depicted in Fig. 5 and Fig. 6… see above comments

Response: Deleted

Query: Line 325 – delete presented in Table

Response: Deleted

Query: Lines 330-338 = remove Fig captions from main MS text – refer to guidelines.

Response: Figure captions removed from the main MS text.

Query: Table 4 (don't just copy table from program) = Table Header replace 1 2 3 4 with actual locations: Goa Japan Same with Table 5.

Response: As suggested by the reviewer, the Tables of genetic distance (Tables 4 & 5) have been reformatted in the revised manuscript.

Query: Line 317-372 Fig caption see guidelines.

Response: Done

<u>Discussion</u>

Query: Line 390-394 Fig caption – see guidelines

Response: Done

Query: There are repeated results in the discussion – delete them – you already have these in your Result section. Discuss your results.

Response: As suggested by the Reviewer, results have been deleted from the Discussion Section.

Query: Line 405-415 remove results repeated – already in Result section Fig. 2 and Fig 3 = combine or place one in suppl material Figure 1 & 8 combine = Fig 1 could be inset to the bigger Map Fig 8.

Response: Following the comments made by the Reviewer, we have modified the discussion comprehensively. in the revised manuscript. Now, it is hoped the Discussion has improved by removing the repetition of Results. We tried to combine the Fig. 2 and Fig. 3, the quality of the Figure was lost. Similarly, we tried to integrate Fig. 1 to bigger map of Figure 8, but it did not work properly in terms of picture quality and alignment.

<u>Reviewer #2:</u>

Comment: Really nice description of morphological and molecular characters for the range expansion of *H. mohnikei* in India. This is a really interesting seahorse that requires more studies on its distributional range within the Indo-Pacific region.

Response: We thank the Reviewer for the excellent review with positive comments and appreciation of our work. We agree with the Reviewer that further studies on the distributional range of *H. mohnikei* within Indo-Pacific region in collaboration with other researchers in Southeast Asia would help in better understanding its evolutionary and biogeography.

Reviewer #3:

General Comments

Comment: This paper represents a significant contribution to the global knowledge of seahorse diversity in the Arabian Sea, and represents novel information that is interesting from both evolutionary and conservation perspectives. I suggest that the authors revise the manuscript to have more of a focus on the biogeography of seahorses in Southeast Asia, which is outlined nicely by Lourie and Vincent (2004). [Lourie, S.A. and Vincent, A.C., 2004]. A marine fish follows Wallace's Line: the phylogeography of the three- spot seahorse (*Hippocampus trimaculatus*, Syngnathidae, Teleostei) in Southeast Asia. Journal of Biogeography, 31(12), pp.1975-1985]. The manuscript is important and should be published, but requires a thorough re-write to correct the grammar (see specific comments below). The discussion needs to more clearly outline that this is likely to be an established population, but with the possibility that these specimens are vagrants.

Response: We gratefully thank the Reviewer for excellent review with positive comments/suggestions on the manuscript. As suggested by the Reviewer, the biogeography of seahorse populations in southeast Asia has been focussed in the revised manuscript. The paper by Lourie & Vincent (2004) dealing with the phylogeography of three-spotted seahorse, *H. trimaculatus* has been referred in the manuscript. Accordingly, the discussion on biogeography of seahorse populations has now been included in the discussion. Additional minor modifications have been made to improve overall readability and to clarify certain statements. The grammatical errors have also been fixed as per the specific comments/edits provided by the Reviewer.

The reviewer may agree that more samples of *H. mohnikei* would be needed to arrive at a definite conclusion for ascertaining whether the present sighting is a previously unreported established populations or vagrants. Based on this present reporting, our efforts for more specimens of *H. mohnikei* are still being pursued. With more number of specimens from Goa waters, the biogeography of this small seahorse species in southeast Asia could be addressed comprehensively. Of course this would help in better understanding from both evolutionary and conservation view points.

Comment: The information regarding ocean circulation should be provided in the methods section, and figures should be added to display other seasons as well. This should then be linked in the discussion to how the species got to Goa. An effort should be made to determine where this species originates biogeographically and to clarify in which direction it dispersed over time.

Response: As suggested by the reviewer, now the Figure describing the ocean circulation has been moved to Materials & Methods section (Fig. 4). The role of the ocean circulation in the Indo-Pacific region in facilitating the dispersal of *H. mohnikei* from its previously reported geographical range into the coastal waters of Goa has been now discussed in the revised manuscript.

Comment: Overall, this manuscript requires a substantial amount of work to be made publishable, but I do not consider this a 'major revision', as the overall structure and content need to change very little. I suggest publishing this manuscript in PLoS ONE after these extensive minor revisions have been made.

Response: The authors are thankful to the Reviewer for positive feedback on the manuscript. Constructive suggestions provided by the Reviewer are appreciated. Now the manuscript has been revised following the minor revisions suggested by the Reviewer.

Specific comments

Query: Line 27: use 'geographic range' rather than 'distribution range'; do this throughout the manuscript

Response: As suggested, ‘distributional range’ changed to ‘geographical range’ in the revised manuscript.

Query: Line 40: "hitherto known range of Japan and Vietnam" is incorrect. Aylesworth et al (2016) have already reported this species from the Gulf of Thailand and western Thailand.

Response: We honestly agree with the Reviewer’s comment. Now, the statement has been modified to accurately represent the fact in the revised manuscript.

Query: Line 43: 'conspecificity' refers to individuals within the same species; it appears here that the authors are suggesting that the specimens from Japan are of a different species. I suggest rewording to state that the specimens examined in this study are closer genetically to those in Vietnam and Thailand than they are to those in Japan. Unless there is evidence to the contrary, they are all conspecifics.

Response: Yes, Agreed. As suggested by the Reviewer, the statement has been reworded to genetically closer in the revised manuscript.

Query: Lines 56-57: 'unnoticeable from coastal habitat' is unwieldy. I suggest deleting this sentence.

Response: Sentence deleted as suggested.

Query: Lines 57-59: This is an incomplete sentence that lacks a subject; 'thick coastal marine habitats' is ambiguous. I suggest deleting this sentence as well.

Response: As suggested by the Reviewer, the sentence as been modified to convey the intended meaning.

Query: Line 60: at least three species have been described since Lourie et al. (2016): *Hippocampus casscio, H. haema*, and *H. japapigu*

Response: We totally agree with the Reviewer and we are sorry for missing out this. With the reporting of additional 3 seahorse species since Lourie et al (2016), the statement has been modified to include this. i.e. 44 instead of 41. Appropriate reference (IUCN Seahorse, Pipefish and Stickleback Specialist Group (2019). https://www.iucn-seahorse.org (downloaded on 01/11/2019) has also been cited.

Query: Lines 63-64: the validity of *H. montebelloensis* and *H. borboniensis* is disputued by Lourie et al. (2016). Although it is not necessary to agree with this, it is necessary to make note in the manuscript.

Response*:* We have noted the point raised by the Reviewer. Accordingly, the sentence has been modified by including a caveat. Added a sentence 'The validity of *H. bornoinensis* and *H. montebelloensis* as separate species however, is currently under revision due to their synonymity with *H. kuda* and *H. zebra*, respectively' (Lourie et al., 2016).

Query: Lines 69-70: "(e.g. pelvic and caudal fins), high variability and overlapping of body proportions, colour (camouflage) and skin filaments" should read "(e.g. pelvic and caudal fins), and have high variability of and overlapping body proportions, colour (camouflage) and skin filaments.

Response: As suggested by the Reviewer, the sentence has been modified.

Query: Line 73: 'relationships' rather than 'relationship'

Response: As suggested, 'relationship' changed to 'relationships

Query: Lines 80-81: currently reads "However, instances of small migration of seahorses in search of proper habitat", should read "However, instances of limited migrations by seahorses in search of proper habitat

Response: As suggested, the statement has been modified.

Query: Lines 83-84: I would not consider oceanic current to be 'stepping stones'. Although currents facilitate dispersal, the real 'stepping stones' would be patches of habitat along the way.

Response: We agree with the Reviewer to a certain extent. Oceanic currents may facilitate long distance dispersal but indirectly facilitate stepping stone mode for short distance dispersal. Accordingly, the sentence has been altered.

Query: Lines 88-89: reference [35] (Aylesworth et al. 2016) report this species from western Thailand, not "from southeastern India to Korea and Japan"

Response: We are thankful to the Reviewer for pointing out this mistake. The statement has been now corrected in the revised manuscript**.**

Query: Lines 89-91: 'Previous reports' should include the Aylesworth et al (2016) observations from western Thailand. As stated currently, it appears the authors of this manuscript have extended the range from Vietnam to western India, when in fact the manuscript is only extending it from eastern India to western India. (see Figure 27 in Lourie et al. 2016).

Response: Agreed. As suggested by the Reviewer, the sentence has been corrected in the revised manuscript. Yes it is true that the reported new range in the study is from eastern India to western India.

Query: Lines 90-92: this species is not listed as Vulnerable because of 'recent exploitations'; it is listed as Vulnerable because of a suspected reduction in population size of >30% over the past 10 years due to fisheries exploitation.

Response: We totally agree with the Reviewer's comment. As suggested, now the statement has been modified to convey the correct rationale for listing *H. mohnikei* as 'VULNERABLE'

Query: Line 92: the word 'vulnerable' should be capitalized, as it is a formal IUCN Red List Category.

Response: In order to comply with the format of IUCN, the word 'Vulnerable' changed to 'VULNERABLE'

Query: Line 98: there needs to be a bridge sentence: the current text indicates that surveys found no new specimens and then goes straight into the fact that more specimens were found. The fact that the specimens examined were caught as bycatch in bag fisheries needs to be explained here, prior to stating the expansion.

Response: We thank the Reviewer in pointing out this error. In order to have a smooth flow, a bridge sentence indicating the specimens examined were based on the incidental catch has been inserted in the revised manuscript (Lines, 111-114).

Query: Lines 101-102: how do we know that this is a recent expansion of the range? Presumably this species could have been present here all along without being detected.

Response: While it is true that this species could have been here without being detected all these years, but we are the first ones to report its occurrence along the west coast of India evidenced with both morphological and molecular data. Previous report of its occurrence from Indian waters by Thangaraj and Lipton (2007) is from Southeastern coast of India and is solely based on the morphological data of a single specimen. As mentioned in the manuscript, intense sampling from coastal waters of west coast of India and comparison of *H. mohnikei* populations from Southeast Asian regions are required for comprehensive biogeography of this small seahorse species in the Indo-Pacific Region.

Query: Line 109: "The collection site of seahorses" - suggest rewording is "The locality at which the examined seahorse specimens were collected from"

Response: Corrected as suggested.

Query: Line 119: suggest deleting 'characterized by'

Response: Deleted

Query: Line 157: replace 'three time counting' with 'triplicate counting'

Response**:** As suggested**,** 'three time counting' changed to 'triplicate counting'

Query: Line 158: change 'were also taken in case of male specimens' to 'were measured in all male specimens'.

Response: Corrected as suggested by the Reviewer.

Query: Line 230: remove 'almost'

Response: Removed

Query: Lines 232-233: Are there two cheek spines and two eye spines in total, or on each side of the head (so four)?

Response: Two prominent cheek spines and double rounded spines below the eye (each on the either side of the head) were observed

Query: Table 1: header needs to be repeated at top of second page

Response: We are sorry for the formatting issue. The size of the Table 1 has now been adjusted so that all the data of the Table to appear on the singe page.

Query: Line 258: replace 'A comparative' with 'A comparison of' Line 260: change 'was' to 'were'

Query: Line 265: delete 'the'

Reply: Deleted.

Query: Line 266: it appears the heading for Table 2 became separated from the rest of the table.

Response: We are sorry for this. Table 2 has been reformatted and positioned to so that the heading and the data to appear on the single page.

Query: Line 266: replace 'distinguishes' with 'distinguish'

Query*:* Line 337: replace 'seahorses' with 'seahorse'

Response: ‘seahorses’ replaced with ‘seahorse

Query: Lines 359-370: Unless the authors actually collected field data regarding ocean circulation, this section should remain entirely in the methods section where the study site is described.

Response: As suggested by the Reviewer, the Figure (Fig. 8) describing the ocean circulation has been moved to Materials & Methods section (now, Fig. 4). As mentioned in the manuscript, the basin-wide prevailing winter circulation (December–February) in the Indo-Pacific region based on the daily data for the 10 years (2007 to 2017) has been prepared and presented.

Query: Lines 386-388: should read "In northeast Asia, *H. mohnikei* is reported from China and South Korea".

Response: As suggested, the sentence has been corrected.

Query: Line 397: change 'morpho-molecular' to 'morphological and molecular'; delete 'is the most recent'

Response: As suggested, ‘morpho-molecular’ changed to ‘morphological and molecular’. Deleted ‘is the most recent’ in the revised manuscript.

Query: Line 398: delete 'distributional'

Response: Deleted as suggested

Query: Line 405: delete 'of' after 'constructed'

Query: Line 410: replace 'sequences' with 'sequence'

Response: Replaced ‘sequence’ with ‘sequences’

Query: Line 421: the term 'disjunct' is misused here. I don't believe there is any evidence to suggest any disjunctions in the population.

Response: We totally agree to the Reviewer’s view. In the revised manuscript, the term ‘disjunct’ has been removed.

Query: Lines 421-423: this statement needs some nuance. Genetic differences within a species do not necessarily mean that cryptic species exist. These difference can mean that there are cryptic species, but the authors should state what a defensible threshold for species delineation might be (i.e. at what % difference would we likely consider them separate species?).

Response**:** Agreed. The sentence has been modified. a 2% threshold genetic divergence was adopted for assessing intra-specific differences (Lourie et al., 2016). The statement regarding 'cryptic species' has been modified, accordingly.

Query: Line 425: replace 'on' with 'of' Line 431: replace 'report' with 'data'

Response: As suggested by the Reviewer, necessary edits have been incorporated in the revised manuscript

Query: Line 451: again I am not aware of any evidence to say that the population is disjunct

Response: We agree with the Reviewer and thank for pointing out this mistake. Now, the term ‘disjunct’ replaced with ‘geographically disconnected’

Query: Lines 474-475: replace 'attain a settlement phase (benthic)' with 'settle on the benthos'

Response: Replaced 'attain a settlement phase (bethic)' with ' 'settle down into sessile habitats (benthos)'

Query: Line 476: the authors need to provide evidence that the population is disjunct

Response: Now, the term ‘disjunct’ replaced with ‘geographically disconnected’

Query: Line 484: delete 'a'; replace 'promoting' with 'permit'

Response: Necessary edits have been done**.**

Query: Line 490: delete 'as'

Response: Deleted

Query: Lines 488-500: Is there evidence that this species originated in China/Japan? This section reads as though the species dispersed here recently. How can we be sure it didn't originate in India and disperse to the east? The prevailing currents are only shown for one season.

Response: We thank the Reviewer for this interesting suggestion. Although there are no published evidences to show that this species originated in China/Japan but original type specimens were studied from Japan and Vietnam. At the same time, *H. mohnikei* might be present in other localities, but not reported or overlooked.

The possibility of *H. mohnikei* originating in India and its dispersal to east cannot be ruled out. However, this require supportive genetic data from the coastal waters of India for conclusive results. Towards this, we are planning to collect more specimens of *H. mohnikei* and sequencing of mitochondrial DNA (mtDNA) gene. Furthermore, genetic data of *H. mohnikei* from Southeast Asia region is also required for comparison. The need for such a study has been now included in the Conclusions Section.

Query: Line 506: change 'entail seahorses to flow downstream' to 'force seahorses downstream'

Response: As suggested, necessary corrections have been incorporated in the revised manuscript

Query: Line 518: delete 'inhabiting'

Response: Deleted ‘ínhabiting'

Query: Line 520: it's not clear what the authors mean by 'translocated populations'.

Response: We are sorry for the usage of incorrect term here. Now, 'translocated' replaced with ‘geographically disconnected’

Query: Line 528: Translocation suggests that humans collected the seahorses elsewhere and relocated them to Goa. I don't think that is what this paper suggests - the evidence is clear that this is probably an extension of their natural range based on new information. I suggest deleting point (i). This sentence should state that these are either vagrants or a previously unreported established population.

Response: We are grateful to the Reviewer for pointing out this mistake and incorrect usage of the term, ‘translocation’ here. Concurring to the Reviewer’s suggestion, the point (i) has been deleted in the revised manuscript.

Reviewer #4:

General Comments

Comment: Mr. Rayadurga and colleagues are to be commended for their efforts to understand the taxonomic identity and biogeography of a rare and previously poorly documented seahorse species occurring on the central west coast of India. As the authors state, seahorses globally are in decline, driven by their demand as an ingredient in traditional Chinese medicine as well as by degradation of their coastal marine habitat by human activities. The taxonomy and distribution of seahorses must be further clarified if seahorse populations are to persist in the face of these pressures.

Response: We thank the Reviewer for positive feedback on the manuscript.

Comment: While indeed it is intriguing to find and identify several specimens of *H. mohnikei* in the Goa region, unfortunately the methods presented in this manuscript do not support the conclusions the authors draw, as explained below. In addition to the disconnect between the analyses and their conclusions, the findings of this paper are more appropriate for a more specialized journal, such as Marine Biodiversity Records or Zootaxa, than for PLoS ONE. The manuscript also needs a thorough editing by a native English speaker for grammar and clarity of prose. For all of these reasons, as explained in more detail, I cannot recommend this manuscript be published in PLoS ONE.

Response: We appreciate the Reviewer for providing the constructive criticisms. Here, we provide the point by point rebuttal/clarifications sought by the reviewer. While we agree that one of the shortcomings could be small sample size, nevertheless, the limited dataset was robust enough to provide interesting insights into *H. mohnikei*—small and highly migratory seahorse species. As the Reviewer agree that it is quite hard to obtain sufficient number of seahorse specimens for the study due to their cryptic nature and low population density. The revised version of the manuscript is more organised and focussed after incorporating the suggestions and corrections made by all four reviewers and performing additional analysis. We chose PLoS ONE as a medium of publishing our results, firstly because of the multidisciplinary nature of the journal and secondly the 'Open Access Policy' of the journal—which would allow wider dissemination of the results, quickly. In addition, we have seen few similar papers of this nature published

Comment: While indeed it is likely that the 4 analyzed seahorse specimens are *H. mohnikei*, neither the morphological or the genetic analyses have been conducted in a way that support the authors conclusions.

Response: We thank the reviewer for constructive feedback. Following the Reviewer's comment, we have made two major changes to the manuscript which are explained point by point below.

Comment: For the morphometric analysis, it is essential to include one or preferably more vouchered specimens of *H. mohnikei* in the analysis to conclusively determine that the morphometric cluster analysis supports the identification of the specimens in question. By examining only the 4 specimens in question against specimens from two other distinct seahorse species, the authors have only demonstrated that the unidentified seahorse specimens are NOT *H. kuda* or *H. trimaculatus*. But this analysis cannot be used to conclude these unidentified specimens ARE *H. mohnikei*. For that, you would need to also measure one or more vouchered specimens of *H. mohnikei* and show that the Goa specimens cluster together with known *H. mohnikei* morphometric data.

Response: As suggested by the Reviewer, now a rigorous morphological and morphometric analysis has been conducted A comparative assessment of diagnostic morphological features and morphometric characters of Goa specimens with all vouchered specimens of *Hippocampus mohnikei* has been conducted. Results of comparative analysis have been tabulated under Table 3. This comparison demonstrated that the unidentified specimens from Goa are *H. mohnikei* as the most of the diagnostic morphological characters of Goa specimens matched with those of vouchered specimens.

Comment: The case with the genetic data is the same. The correct analysis would be to download ALL VOUCHERED *H. mohnikei* COI and cytb sequences, align them, conduct an analysis of the correct model of molecular evolution for this dataset (there is no justification for why the authors used K2P - how was it determined that this was indeed the correct evolutionary model for these data?), and conduct a rigorous phylogenetic analysis that assesses the position of the 4 Goa specimens among all other known *H. mohnikei* sequences for both genes.

By choosing only a few sequences from other *H. mohnikei* specimens to compare to, the authors are depriving this analysis of the dataset required to more fully understand the identity and phylogeographic relationships of the Goa specimens.

Response: We thank the Reviewer for pointing out this mistake. As suggested by the Reviewer, we have downloaded all vouchered *H. mohnikei* COI and Cyt *b* sequences from GenBank (S1 Table), aligned them and conducted a rigorous phylogenetic analysis. This rigorous analysis allowed us to assess the phylogeographic analysis of 4 specimens for Goa among all other known *H. mohnikei* sequences for both COI and Cyt *b* genes from the Indo-Pacific region. K2P parameter was used to assess the degree of genetic divergence within *H. mohnikei* populations from different geographic regions.

Comment: The results of the genetic analysis do not support the conclusions the authors make, but potentially there is something quite interesting to be investigated here. The authors claim that the taxonomic identity of the Goa specimens in confirmed because the sequences of the putative *H. mohnikei* from Goa are nearly identical to just 3 other *H. mohnikei* sequences from southeast Asia. And here is where it gets even more interesting, while also pointing to the lack of support for the authors conclusions. The pairwise genetic distances reported between the Goa specimens and the 3 SE Asia sequences for both Cytb and COI are ENORMOUS, and absolutely cannot reflect intraspecific genetic variation. The authors are reporting 3-10% distance in COI, where most valid species can be separated at the 3% level. The cytb differences are so large as to reflect saturation in mutations - you basically cannot get larger genetic distances than 18% or so, and levels that high almost always reflect completely different species. These results only validate the need to include all known vouchered specimens of *H. mohnikei* in the analysis, to better understand the clustering among the 100 or so available specimens in GenBank and the Barcode of Life databases.

There are several possible reasons for these findings: an inaccurate sequence alignment, the need for a more sophisticated model of molecular evolution that can account for the possibility of multiple mutations at a given site, or that the specimens from Goa really are so genetically distinct they have diverged significantly on their evolutionary pathway. A more rigorous genetic analysis is the only way to understand what these 4 mystery specimens really represent.

Response: Post-sequencing of remaining 3 Goa specimens for COI and Cyt *b* genes (now, total four) and aligning them with all vouchered downloaded *H. mohnikei* COI and Cyt *b* sequences from GenBank, the results of phylogenetic analysis has improved greatly. The revised manuscript has addressed most of the the apprehensions, particularly on the genetic distance raised by the Reviewer. The manuscript has been now checked for English language.

Comment: I recommend the authors shorten their paper to a scientific note format and publish this work in a more specialized journal such as those listed above. By publishing the sequences and placing them in the public record, they will have done a great service to adding important information regarding the continuing mystery of seahorse taxonomy and distributions, for which they will be acknowledged.

Response: We thank the Reviewer for this suggestion. Considering the sighting of *H. mohnikei* along the west coast of India has biogeographic and conservation significance, the manuscript attempts to address the probable reasons for its occurrence and range expansion. We wanted the manuscript to be quite different from routine Scientific Notes merely reporting the occurrence of this highly migratory small seahorse species. We hope this manuscript would provide new information and deeper insights which may be helpful in addressing the continuing mystery of seahorse taxonomy and distributions.
