# Supplementary material for: Morphological and molecular evidence for first records and range extension of the Japanese seahorse, Hippocampus mohnikei (Bleeker 1853) in a bay-estuarine system of Goa, central west coast of India
Source: PLoS One. 2020 Mar 24;15(3):e0220420. doi: 10.1371/journal.pone.0220420 (PMC7092974; doi:10.1371/journal.pone.0220420)
Supplement: Attachment — Submitted filename: 2nd review.docx [file pone.0220420.s005.docx]

**Morphological and molecular evidence for range extension and first occurrence of the Japanese seahorse, Hippocampus mohnikei (Bleeker 1853) in a bay-estuarine system of Goa, central west coast of India**

**2^nd^ revision Feb. 2020**

Great improvement! So much easier to read and follow!

Only 2 minor edits:

Lines 61-64 please see reviewers (#1 & #3) comments: delete sentence line 61-62

Lines 62-64 either delete sentence or change sentence it is still awkward.

What you’re trying to say is that they are well camouflaged and rare and thus easily missed (not seen) by biologists/divers etc.

Line 150= ~~incidental catch~~ = … landed as **bycatch** … is the correct word (eg. fish caught in nets/bags that was not intended)

Line 147 use the word **bycatch** throughout … incidentally caught as bycatch in bags …

Line 150 dead seahorses landed as bycatch …
