# Supplementary material for: Morphological and molecular evidence for first records and range extension of the Japanese seahorse, Hippocampus mohnikei (Bleeker 1853) in a bay-estuarine system of Goa, central west coast of India
Source: PLoS One. 2020 Mar 24;15(3):e0220420. doi: 10.1371/journal.pone.0220420 (PMC7092974; doi:10.1371/journal.pone.0220420)
Supplement: Attachment — Submitted filename: Response to Reviewers comments.docx [file pone.0220420.s007.docx]

**Response to Comments/suggestions made by the Reviewers**

**Manuscript ID number**: **PONE-D-19-19415R1**

**Manuscript title**: Morphological and molecular evidence for range extension and first occurrence of the Japanese seahorse, *Hippocampus mohnikei* (Bleeker 1853) in a bay-estuarine system of Goa, central west coast of India

**Authors**: Sushant Sanaye, Rakhee Khandeparker, Rayadurga Anantha Sreepada, Mamatha Singanahalli Shivaramu, Harshada Kankonkar, Jayu Narvekar, Mukund Gauthankar

We are thankful for the reviewers for their constructive criticisms/suggestions on the revised version of the manuscript and their inputs have substantially improved the quality of the manuscript.

Below, we provide a point-by-point response as to how the feedback received from the Reviewers (#1 and #3) has been incorporated in the revised manuscript.

This manuscript is a resubmission of manuscript PONE-D-19-19415R1 with our response to the questions made by the reviewers. The Academic Editor handling this manuscript is Dr. Rui Rosa

**Reviewer #1:**

Reviewer #1: My previous comments have all been answered. I still have 2 minor edits. Please see attached word file.

Great improvement! So much easier to read and follow!. Only 2 minor edits:

***Response:*** We thank the Reviewer for the excellent review and the positive comments on the manuscript. Constructive suggestions provided by the Reviewer are appreciated.

***Minor Edit # 1***

Lines 61-64 please see reviewers (#1 & #3) comments: delete sentence line 61-62

Lines 62-64 either delete sentence or change sentence it is still awkward.

What you’re trying to say is that they are well camouflaged and rare and thus easily missed (not seen) by biologists/divers etc.

***Response:*** As suggested by the Reviewer #1 and also by the Reviewer #3, the sentences, 61-64 have been modified in the revised manuscript as below:

However, these biological traits often also make them challenging for scientists to research and quantify. ~~Due to their cryptic nature, their ability to camouflage and the sparse distribution, seahorses become almost unnoticeable and thus restricting the biologists for their identification in dense coastal marine habitats~~

***Minor Edit # 2***

Line 150= ~~incidental catch~~ = … landed as **bycatch** … is the correct word (eg. fish caught in nets/bags that was not intended)

Line 147 use the word **bycatch** throughout … incidentally caught as bycatch in bags …

Line 150 dead seahorses landed as bycatch …

***Response:*** As suggested by the reviewer, the sentences under line numbers, 147-150 have been corrected as bycatch. However, there is a significant differences between countries between with regard to the interpretation of terms, bycatch’’ and ‘incidental catch’ exists. FAO’s explanatory notes on these terms can be found at the below link:

<http://www.fao.org/3/y5936e/y5936e08.htm>

**Reviewer #3:**

The authors have substantially improved the manuscript based on my previous suggestions. However, there is still a substantial amount of wordsmithing that is needed to make this manuscript publishable.

In particular, there is some confusion around active and passive dispersal (a one-time event) vs migration (which usually happens seasonally). I suggest the authors have a thorough read-through to ensure these terms are differentiated appropriately throughout the text, and particularly in the discussion and conclusions.

Please see the attached pdf with my edits and comments for suggested changes.

***Comments***: Corrections to the Title of the manuscript

***Response:*** As suggested by the Reviewer, the title has been modified to 'Morphological and molecular evidence for first records and range extension of the Japanese seahorse, *Hippocampus mohnikei* (Bleeker 1853) in a bay-estuarine system of Goa, central west coast of India

***Response***: We gratefully thank the Reviewer for excellent review with positive comments/suggestions on the manuscript. As suggested by the Reviewer, all the suggested edits indicated in the PDF have been incorporated in the revised manuscript.

Here, we provide following responses to the specific queries pointed out by the Reviewer.

**Comment:** Shifting & deletion of Lines 106-114

**Response:** These lines form the background and main purpose of undertaking the present study. Therefore these lines retained in order to have to a flow and continuity in the Discussion also.

***Comment:*** Why only the winter season? It needs to be explained why this data was only collected over one season.

***Response:*** The Indo-Pacific region encompasses the East Asian countries and Indian subcontinent comes under the influence of strong seasonal monsoon wind reversal and associated reversals in the surface currents. It is only during winter monsoon season, i.e. from November until February, the surface circulation in the north Indian Ocean including the Indo-Pacific region experiences currents pattern is from east to west which is shown in Fig. 4.

During summer monsoon season (June to September), the surface current in this region reverses and at this time of the year current flows from west to east. Although the prevailing currents during summer monsoon season was also examined but did not present as the circulation pattern during the summer monsoon cannot support a passive dispersal of marine fish species from east to west.

Due to lack of supportive genetic data from the coastal waters of India and from South East Asia region, the possibility of *H. mohnikei* originating in India and its dispersal to east was not considered in the present study. With the availability of genetic data from larger sample size and sequencing of mitochondrial DNA (mtDNA) gene, it may be possible to understand the evolutionary and biogeography of *H. mohnikei* in the Indo-Pacific region. This has been mentioned in the Conclusion section of the revised manuscript.

Now, the rationale for focusing on winter circulation has been added at the beginning of the sub-section Prevailing ocean circulation’ under the lines 260–268 in the revised manuscript.

***Comment***: were genetic comparisons also made with these species from that area?

***Response*:** Phylogenetic trees constructed with COI and Cyt *b* genes of *H.* *mohinkei* with *H. kuda* and *H. trimaculatus* separated out. Genetic sequence data of *H. kuda* and *H. trimaculatus* in fact were included in the pre-revised manuscript. But were replaced with a new set of phylogenetic trees (comparing the vouchered specimens) as suggested by one of the Reviewers. Since the morphological features and meristic characters of Goa specimens matched with that of vouchered specimens of *H. mohnikei,* a detailed genetic analysis of Goa specimens was performed for species reconfirmation and genetic placement of Goa specimens.

***Comment:*** Line Nos. 351-353: combine all accession numbers in a single set of brackets.

***Response*:** GenBank accession Numbers refer to sequences of COI and Cyt *b* genes. Combining all the sequences into a single bracket would be confusing. Hence the status quo retained.

***Comment*:** Figures 6 and 7 are too low-resolution and are not discernible

***Response***: The probable reason for reduction in the resolution may be due to reformatting issues. Converting PACE generated figures to PDF. New high resolution figures have been now uploaded along with the revised manuscript.

***Comment:*** Lines, 509-511: It's not clear to me why the high density of prey items would lead to a prolonged planktonic phase. Please elaborate.

Lines, 517-519- again, why the prolonged planktonic phase?

***Response:*** The sentence has been modified/restructured to convey the intended meaning. Further, the reasoning for extension of planktonic phase by juveniles of *H. mohnikei* has been provided in the revised manuscript.
